# An imidazoacridine-based TADF material as an effective organic photosensitizer for visible-light-promoted [2 + 2] cycloaddition†

**DOI:** 10.1039/d1sc05098b

**Published:** 2022-01-27

**Authors:** Ethan R. Sauvé, Don M. Mayder, Saeid Kamal, Martins S. Oderinde, Zachary M. Hudson

**Affiliations:** Department of Chemistry, The University of British Columbia 2036 Main Mall, Vancouver British Columbia V6T 1Z1 Canada zhudson@chem.ubc.ca +1-604-822-2691; Department of Discovery Synthesis, Bristol Myers Squibb Research and Early Development 3551 Lawrenceville Road, Princeton New Jersey 08540 USA martins.oderinde@bms.com +1-609-252-5237

## Abstract

Energy transfer (EnT) is a fundamental activation process in visible-light-promoted photocycloaddition reactions. This work describes the performance of imidazoacridine-based TADF materials for visible-light mediated triplet–triplet EnT photocatalysis. The TADF material ACR-IMAC has been discovered as an inexpensive, high-performance organic alternative to the commonly used metal-based photosensitizers for visible-light EnT photocatalysis. The efficiency of ACR-IMAC as a photosensitizer is comparable with Ir-based photosensitizers in both intra- and intermolecular [2 + 2] cycloadditions. ACR-IMAC mediated both dearomative and non-dearomative [2 + 2] cycloadditions in good yields, with high regio- and diastereocontrol. Cyclobutane-containing bi- tri- and tetracylic scaffolds were successfully prepared, with broad tolerance toward functional groups relevant to drug discovery campaigns. Fluorescence quenching experiments, time-correlated single-photon counting, and transient absorption spectroscopy were also conducted to provide insight into the reaction and evidence for an EnT mechanism.

## Abstract 2

This work describes the performance of imidazoacridine-based TADF materials for visible-light mediated triplet–triplet EnT photocatalysis.

## Graphical abstract

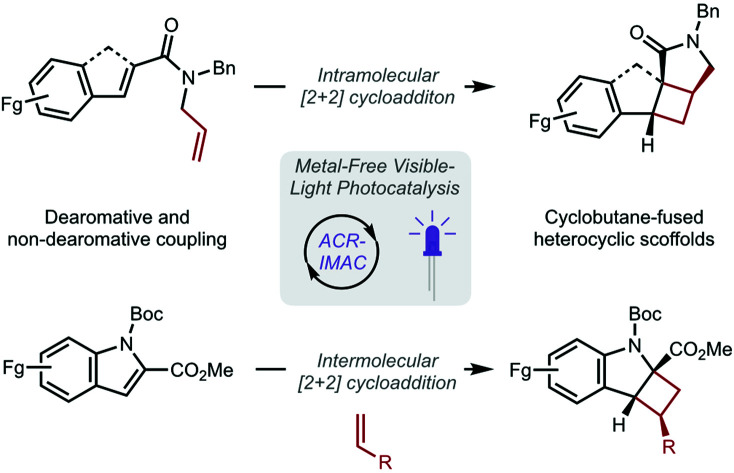

## Introduction

Photocatalysis is an increasingly important tool in organic synthesis, facilitating the rapid preparation of complex molecular scaffolds.^1–3^ Driven by the growing demand for new pharmaceutical building blocks, photocatalytic approaches have provided access to elusive areas of chemical space from structurally simple and readily available precursors. Visible-light photocatalysis can proceed *via* a single electron transfer (SET) mechanism in which reduction or oxidation of a substrate is promoted by an excited photoredox catalyst.^4^ An alternative strategy employs energy transfer (EnT) from a photocatalyst (PC) to the substrate, promoting its sensitization.^5^ The latter approach does not rely on the redox potentials of the substrate, but rather on the triplet excited state energies (*E*_T_) of the substrate and the PC. Photocatalysis by EnT is gaining increasing attention and has been successfully applied to isomerization, cross-coupling, and cycloaddition reactions, among others.^5–8^

Photocatalysts based on heavy transition metals such as Ru or Ir have been studied extensively, due to their high *E*_T_, fast rates of intersystem crossing (ISC) and long-lived triplet excited states.^1,9,10^ However, these catalysts suffer from several drawbacks, including elevated costs, terrestrial scarcity and difficulty in removing the metal from the products. PCs based on more earth-abundant, first-row transition metals have been recently explored, addressing some of these concerns.^11–13^ Metal-free organic PCs, however, have become increasingly attractive alternatives, with many examples of strongly reducing or oxidizing PCs reported to date.^14–20^ Several approaches have been taken to increase the rate of ISC in these materials, including functionalization with heavy halogens, or the use of carbonyl-based PCs such as π-extended benzophenones.^8^

More recently, it has been recognized that materials exhibiting thermally activated delayed fluorescence (TADF) can act as effective photocatalysts, as the design paradigm underlying TADF is intended to facilitate rapid ISC.^21–23^ TADF compounds are most commonly designed with a twisted donor–acceptor architecture, which increases the spatial separation between the HOMO and LUMO. This minimizes the electronic exchange interaction between singly occupied orbitals when the molecule is excited, reducing the energy gap (Δ*E*_ST_) between the lowest singlet (S_1_) and triplet (T_1_) excited states. If Δ*E*_ST_ is small (<0.2 eV, or 4.6 kcal mol^−1^), ambient thermal energy can be sufficient to promote reverse intersystem crossing from T_1_ to S_1_, resulting in rapid interconversion between the two excited states. This paradigm has been explored extensively in organic light-emitting diodes (OLEDs), where TADF materials can be used as emitters for efficient exciton harvesting.^24–28^ TADF materials have also been used as photocatalysts for cross-coupling,^21,29,30^ isomerization,^21^ hydroformylation,^31^ CO_2_ reduction,^32^ and atom-transfer radical polymerization.^33^ Furthermore, TADF materials that exhibit deep-blue fluorescence must have high triplet energies (*E*_T_ > 2.7 eV, or 62 kcal mol^−1^), which can promote reactions that are initiated by an EnT mechanism.

Our group recently reported a series of imidazoacridine-based donor–acceptor materials exhibiting TADF, using structural constraint to provide emitters with narrow emission bands.^34^ These materials exhibit high triplet energies (*E*_T_ = 58.2–69.9 kcal mol^−1^), long-lived triplet excited states, fast ISC, and visible-light absorption, while also behaving as poor oxidants. These properties make them attractive potential sensitizing catalysts for visible-light mediated reactions.

Cyclobutane-fused heterocyclic structures are synthetic targets that can be difficult to access due to the high strain energy and conformational rigidity imposed by the cyclobutane ring.^35^ Despite this, such scaffolds are found in a large number of pharmaceuticals and polycyclic natural products.^36,37^ Photochemical [2 + 2] cycloaddition reactions are a versatile method for accessing cyclobutanes, with recent examples reported by Meggers, You and Yoon.^35–40^ We recently deployed photocatalysis to promote intramolecular [2 + 2] cycloadditions and access cyclobutane-fused bicyclic^42^ and tetracyclic^43^ scaffolds from relatively simple, acyclic compounds. Those cycloadditions all rely on the use of metal-containing photocatalysts. Seeking to unlock the advantages of organo-photocatalysis, we sought out deep-blue TADF materials with compatible triplet energies for use as photosensitizers.

**Fig. 1:**
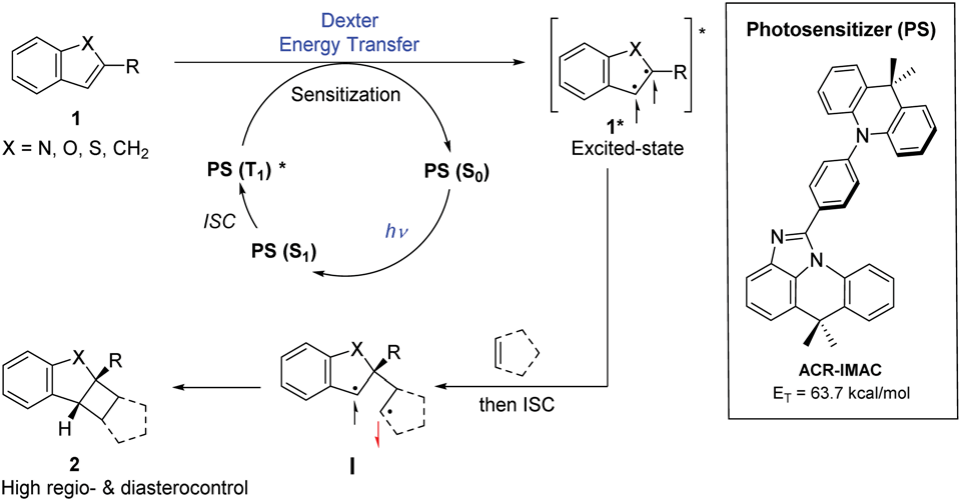
Generalized proposed mechanism for photosensitizer (PS)-promoted [2 + 2] cycloaddition by an energy transfer process (left) and structure of ACR-IMAC (right).

Herein, we show that imidazoacridine-based TADF compounds can act as effective high-energy EnT photosensitizers for [2 + 2] cycloadditions, promoting the synthesis of strained bi-, tri-, and tetracyclic cyclobutane-fused scaffolds. In particular, the high efficiency of ACR-IMAC (Fig. 1) as a triplet photosensitizer make it a suitable organic replacement for iridium-based photocatalysts in both intra- and intermolecular [2 + 2] cycloadditions. Near-quantitative yields were obtained in some cases, with high regio- and diastereoselectivity. Functional groups for derivatization and/or elaboration in drug discovery are well-tolerated, providing access to architecturally complex and versatile scaffolds by organo-photocatalysis using visible light. Finally, fluorescence quenching experiments, time-correlated single-photon counting (TCSPC), and transient absorption spectroscopy (TAS) are used to investigate the mechanism of photocatalysis in this system.

## Results and discussion

Three main criteria must be met for an EnT process to be efficient: (i) the triplet energy of the photosensitizer (PS) must be higher than that of the energy acceptor/substrate; (ii) the photosensitizer must have a high intersystem crossing (ISC) rate that induces a spin inversion; (iii) the photosensitizer must possess a long triplet excited-state lifetime.^11^ The proposed mechanism for [2 + 2] photocycloaddition through an EnT process is described in Fig. 1.^43^ Initially, light absorption excites a photosensitizer (PS) to its S_1_ state, which can undergo a rapid ISC to the T_1_ state. The key step in this mechanism is the intermolecular energy transfer from the PS to substrate 1, inducing the excitation of 1 to a 1,2-diradical-based, triplet excited-state 1*. A regioselective engagement of 1* with an olefin (intra- or intermolecular engagement) would give a 1,4-diradical intermediate, I, which can then undergo a rapid radical–radical combination to provide the cyclobutane-fused scaffold 2 with high diastereocontrol.

To date, the photosensitizing ability of imidazoacridine-based TADF compounds has not been explored in photocycloaddition and other organic reactions. We began by evaluating the competence of seven imidazoacridine-based TADF compounds that carry sufficient triplet energies (*E*_T_ = 58.9–69.9 kcal mol^−1^)^34^ to promote the intramolecular [2 + 2] cycloaddition of *N*-allyl-*N*,1-dibenzyl-1*H*-indole-2-carboxamide (3) (*E*_T_ = 58.7 kcal mol^−1^) to give the cyclobutane-fused tetracyclic scaffold 4 (Table 1).^41^PTZ-IMAC (*E*_T_ = 61.2 kcal mol^−1^) promoted the photocycloaddition of 3 to form the desired cycloadduct 4 albeit in low 9% conversion upon irradiation with violet LEDs (*λ* = 400 nm, see Fig. S7† for spectral profile) in MeCN at room temperature for 18 hours (entry 1). The conversion improved up to 22% in the presence of PXZ-IMAC (*E*_T_ = 58.9 kcal mol^−1^, entry 2) and TolCZ-IMAC (*E*_T_ = 58.2 kcal mol^−1^, entry 3), respectively. Gratifyingly, 100% conversion to the desired cycloadduct 4 was obtained when ACR-IMAC (*E*_T_ = 63.7 kcal mol^−1^, entry 4) was used as the photosensitizer. Surprisingly, CZ-IMAC (*E*_T_ = 64.1 kcal mol^−1^), and TerCZ-IMAC (*E*_T_ = 69.9 kcal mol^−1^), despite their higher *E*_T_, were both less effective sensitizers than ACR-IMAC (entries 4–8). The efficiency of CZ-IMAC and TerCZ-IMAC did not improve either by conducting the reaction with DMSO as a co-solvent or irradiating with a lower wavelength light (entries 4–8). It is noteworthy that the efficiency of ACR-IMAC diminished under blue light irradiation (450 nm, entry 9). The discovery of ACR-IMAC as an effective photosensitizer for cycloaddition prompted the design of CZ-Me-IMAC, which has improved solubility in MeCN. While the product conversion of CZ-Me-IMAC more than tripled that of CZ-IMAC (entry 5 *versus* 11), ACR-IMAC remains significantly higher-yielding. Control experiments showed that the cycloaddition reaction progressed only in the presence of a photosensitizer and light (entries 13 and 14). We also sought to benchmark the impressive performance of ACR-IMAC against TADF materials that have been previously explored as organo-photocatalysts. Although 3DPA2FBN, 4CzIPN and 3CZClIPN all possess sufficiently high triplet state energies (*E*_T_ = 61.1–62. 7 kcal mol^−1^), none have been previously explored as a triplet photosensitizer in [2 + 2] cycloaddition reactions.^11,42^ We found that 3DPA2FBN, 4CzIPN and 3CZClIPN all promoted the cycloaddition of 3 to 4 albeit with lower conversion (entries 15–17). Finally, the use of a metal-based photosensitizer [Ir(dF(Me)ppy)_2_(dtbbpy)]PF_6_ (*E*_T_ = 62.9 kcal mol^−1^)^42^ gave quantitative conversion of 3 to 4 (entry 18). These studies demonstrate that ACR-IMAC is an effective photosensitizer for promoting [2 + 2] cycloadditions and it is comparable to metal-based photosensitizers. Given that the TADF materials presented in Table 1 possess similar triplet state lifetimes,^34^ the high performance of ACR-IMAC may suggest a higher ISC rate compared to the others.^11,32^

**Table 1:** TADF compounds as photosensitizers in a [2 + 2] cycloaddition^a^

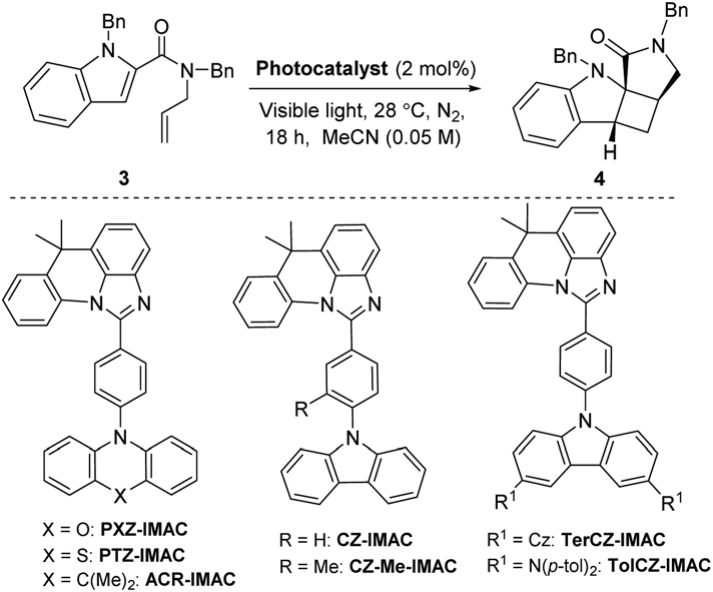				
Entry	Photocatalyst	*E*_T_ b (kcal mol^−1^)	*λ*_ex_ (nm)	Conv.^d^
1	PTZ-IMAC	61.2	400	9%
2	PXZ-IMAC	58.9	400	22%
3	TolCZ-IMAC	58.2	400	21%
4	ACR-IMAC	63.7	400	99%
5	CZ-IMAC	64.1	400	15%
6	CZ-IMAC	64.1	365	23%
7	TerCZ-IMAC	69.9^c^	400	15%
8	TerCZ-IMAC	69.9^c^	365	19%
9	ACR-IMAC	63.7	450	51%
10	CZ-Me-IMAC	63.4	450	51%
11	CZ-Me-IMAC	63.4	400	53%
12	CZ-Me-IMAC	63.4	365	58%
13	—	—	400	NR
14	ACR-IMAC	63.7	—	NR
15	3DPA2FBN	61.1	450	64%
16	4CzIPN	61.6	450	22%
17	3CZClIPN	62.7	450	29%
18	[Ir(dF(Me)ppy)_2_(dtbbpy)]PF_6_	62.9	450	99%

a Reaction conditions: a solution of 2 (0.5 mmol) and photocatalyst (2 mol%) in CH_3_CN (0.05 M) was irradiated with 34–50 W LEDs at 28 °C under an atmosphere of N_2_ for 16–18 h.

b Measured at 77 K in 2-MeTHF as the onset of the phosphorescence *E*_0–0_ band.

c T_1_ and T_2_ bands appear to overlap in the time-gated phosphorescence spectrum of TerCZ-IMAC; energy of the T_2_ band is shown.

d The % conversion was determined by ^1^H-NMR spectroscopy from single experiments and a diastereomeric ratio (dr) of >99 : 1 was observed in all cases. NR = no reaction; Cz = *N*-carbazolyl.

**Fig. 2:**
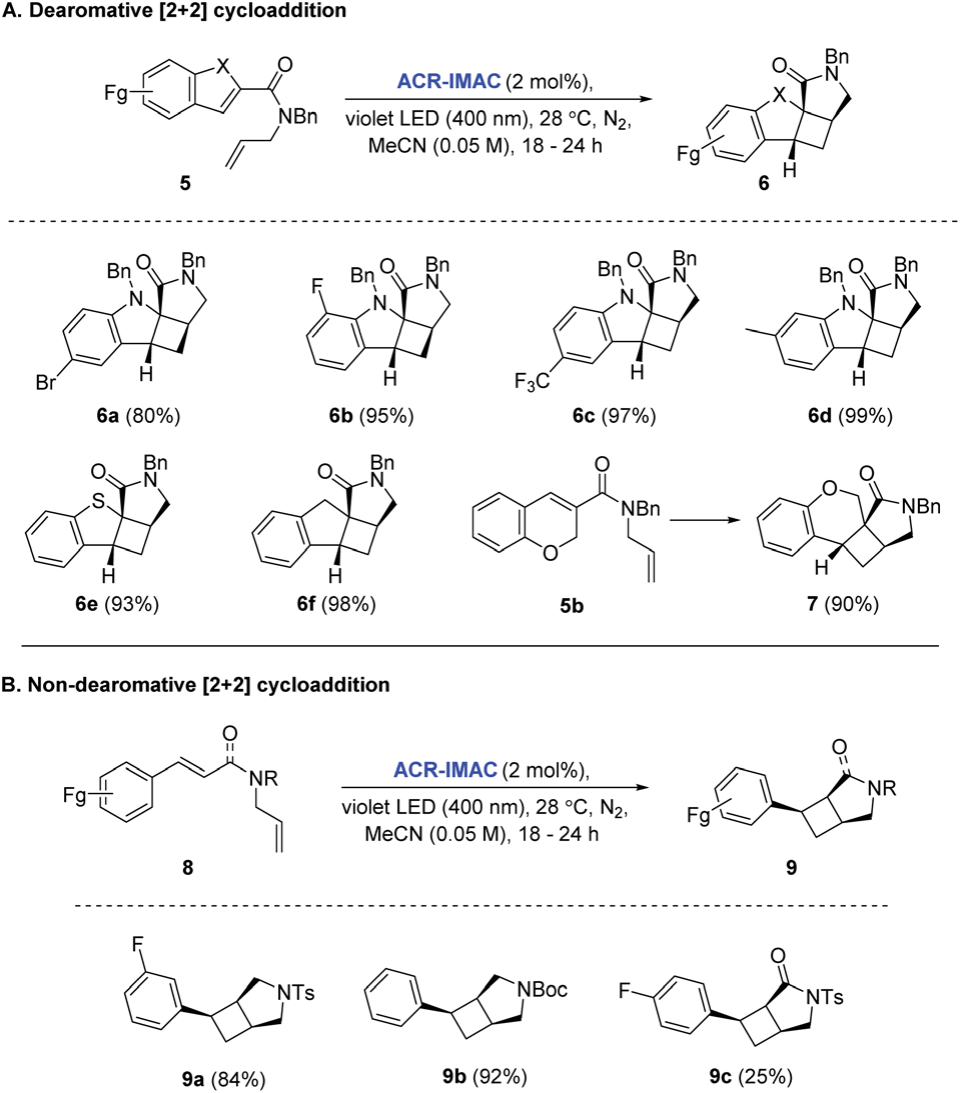
[A] Scope of the dearomative intramolecular [2 + 2] photocycloaddition reaction. [B] Scope of the non-dearomative intramolecular [2 + 2] photocycloaddition reaction. General reaction conditions: indole (0.5 mmol, 1 equiv.), ACR-IMAC (0.01 mmol, 2 mol%), MeCN (0.05 M), violet LEDs (400 nm), <30 °C, 16–18 h. All yields are isolated yields unless otherwise noted and a diastereomeric ratio (dr) of >99 : 1 was observed in all cases.

With the identification of ACR-IMAC as an effective photosensitizer, its general use for [2 + 2] cycloaddition was investigated. Dearomative and non-dearomative intramolecular [2 + 2] cycloadditions were examined in the presence of catalytic amounts of ACR-IMAC (Fig. 2). ACR-IMAC promoted the intramolecular cycloaddition in a high-yielding fashion that was comparable to Ir-based photosensitizers.^41,42^ Indole, benzothiophene, indene, and chromene derivatives tethered with an olefin all underwent a dearomative [2 + 2] cycloaddition in the presence of ACR-IMAC to give the tetracyclic cyclobutane-fused scaffolds in good yield (up to 99%) and with high diastereocontrol (dr > 99 : 1). The efficiency of ACR-IMAC was not impacted by the electronic nature of the substrates, as both electron-withdrawing and electron-donating groups are equally well tolerated (Fig. 2A). Similarly, ACR-IMAC was effective at promoting the cycloaddition of *N*-allylcinnamamines and an *N*-allylcinnamamide (8) to the corresponding aryl-3-azabicyclo[3.2.0]heptanes (9a and 9b) and aryl-3-azabicyclo[3.2.0]heptanone (9c), respectively, with high diastereoselectivity (Fig. 2B).^40^

We recently reported an Ir-based photocatalyzed methodology for the synthesis of cyclobutane-fused scaffolds *via* a dearomatizing, intermolecular [2 + 2] cycloaddition of heterocycles with a range of alkenes.^42^

The ability to promote this important but challenging intermolecular cycloaddition with an organo-photocatalyst would further broaden its application in drug discovery.

**Fig. 3:**
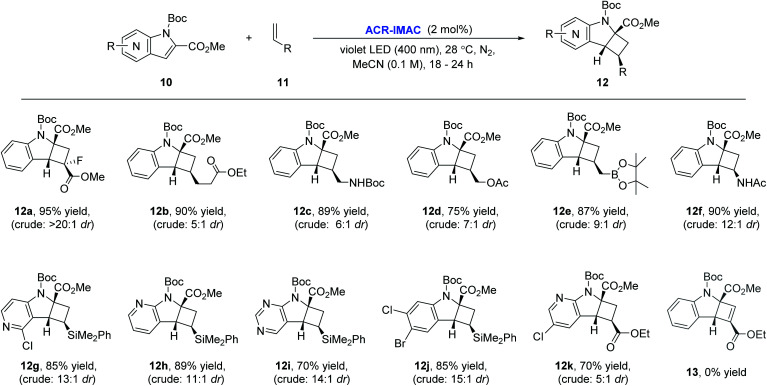
Scope of the dearomative intermolecular [2 + 2] photocycloaddition reaction. General reaction conditions: indole (0.5 mmol, 1 equiv.), alkene (1.5 mmol, 3 equiv.), ACR-IMAC (0.01 mmol, 2 mol%), MeCN (0.1 M), violet LEDs (400 nm), <30 °C, 16–18 h. All yields are isolated yields unless otherwise noted. It is noteworthy that in almost all cases, the minor diastereomers were easily separated by silica gel column chromatography, enhancing the synthetic applicability of this methodology.

To our delight, ACR-IMAC promoted the dearomative intermolecular [2 + 2] cycloaddition of functionalized indoles and azaindoles with a diverse set of alkenes (Fig. 3). The array of alkenes studied that participated in the cycloaddition reaction included synthetic handles amenable to drug discovery programs, including acrylates (12a and 12k), vinyl alkyl ester (12b), allylic derivatives (12c–12e), vinyl amide (12f), and dimethylphenylvinylsilane (12g–12j). While the cycloadditions of indoles and alkenes proceeded in good yields with high regioselectivity and good diastereocontrol, the [2 + 2] cycloaddition of indole with an alkyne (13) did not progress. Similar to the intramolecular processes, the yields and selectivities obtained with ACR-IMAC are comparable to the Ir-based photosensitizers.^41,42^

Spectroscopic investigations were carried out to validate the proposed mechanism for the [2 + 2] photocycloaddition (Fig. 1), which was previously only supported with DFT calculations.^41,42^ We recognized studying triplet-mediated photocatalysis in TADF materials would be particularly challenging since they display both prompt and long-lived fluorescence lifetimes. Unlike metal-based phosphorescent PCs, where 100% of their emission originates from long-lived triplet excited states, the proportion of long-lived emission in a TADF material can, in some cases, be very small (<1%). Indeed, the delayed fluorescence of many TADF materials cannot be observed in solution where long-lived excited states are more easily quenched by molecular motions or collisions. In these situations, careful spectroscopic analysis in the solid state is required to verify that a material does, in fact, display TADF properties.

**Fig. 4:**
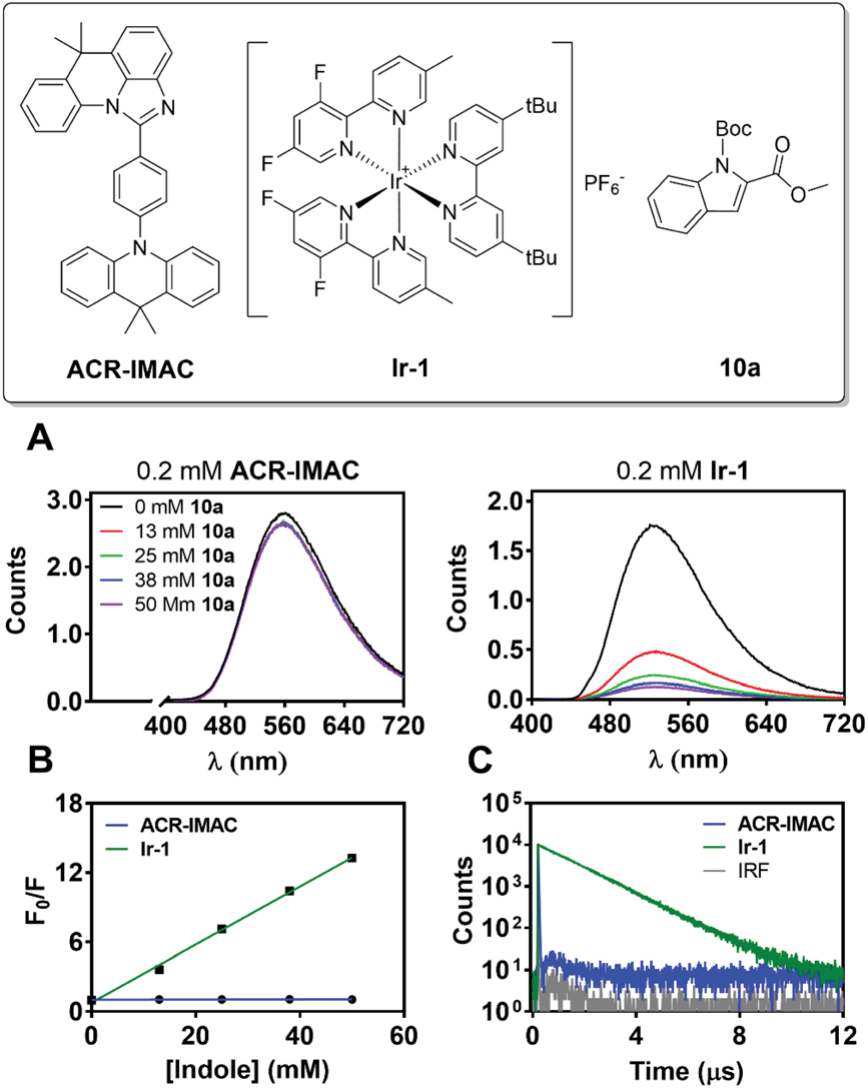
(A) Emission spectra of solutions containing ACR-IMAC (left, 0.2 mM) or Ir-1 (right, 0.2 mM) with varying concentrations of indole 10a in degassed MeCN at *λ*_ex_ = 400 nm. (B) Stern–Volmer quenching analysis comparing the integrated areas of emission spectra presented in (A). (C) Emission lifetimes measured using MCS in degassed MeCN of ACR-IMAC and Ir-1, with excitation using an EPLED at 365 nm.

This is the case for ACR-IMAC, which displays 10% delayed fluorescence in the solid state at 298 K and none observed in solution.^34^ This delayed fluorescence originates from reverse intersystem crossing from T_1_ to S_1_, with the remaining 90% of the emission originating from prompt fluorescence from S_1_. As a result, a typical fluorescence quenching experiment, in which the emission of the PC is quenched in the presence of substrate, provides limited information about the reaction when ACR-IMAC is used. This complexity can be clearly observed when the TADF photocatalyst is compared directly to [Ir(dF(Me)ppy)_2_(dtbbpy)]PF_6_ (Ir-1), a phosphorescent photocatalyst used previously for [2 + 2] cycloadditions (Fig. 4).^42^

When both photocatalysts are treated with increasing concentrations of *N*-Boc-indole-2-carboxylic acid methyl ester (10a) under a N_2_ atmosphere, the phosphorescence of Ir-1 shows clear evidence of quenching (Fig. 4A), while that of ACR-IMAC appears unaffected (Fig. 4A and B). The high oxidation potential of *N*-acetyl indole (*E*^ox^_1/2_ = 1.2 V *vs.* Ag/AgCl)^43^ precludes a SET event and the observed quenching of Ir-1 (*E*^red^_1/2_ [*Ir^III^/Ir^II^] = +0.97 V *vs.* SCE)^44^ can be attributed to EnT. While this result would suggest that EnT mechanism is not operative if ACR-IMAC were a phosphorescent emitter, more careful investigation is required due to the low proportion of available triplets. We also note the importance of correcting these quenching data for inner filter effects, as the indole substrates used here are weakly absorbing themselves (see ESI†).

**Fig. 5:**
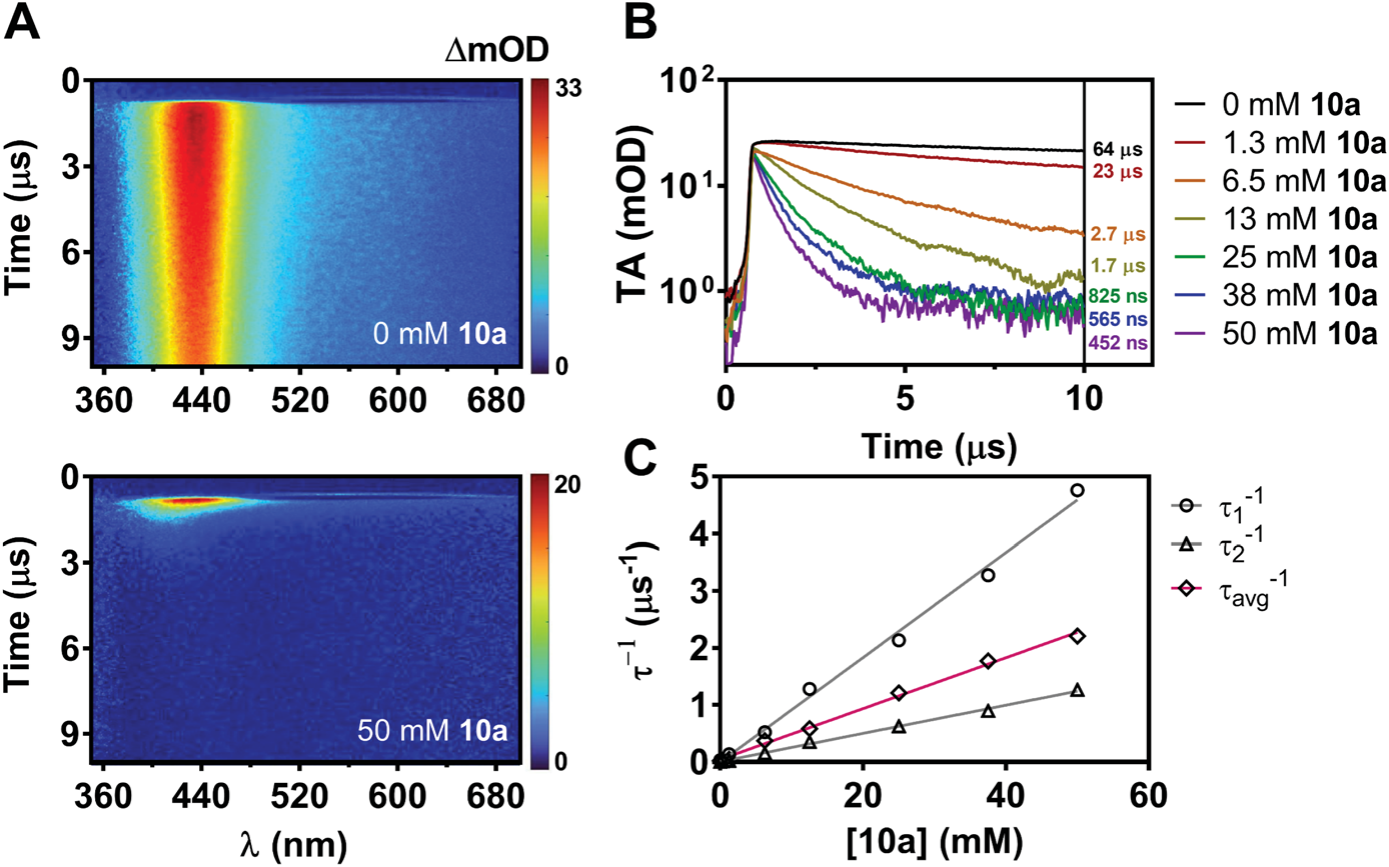
(A) Two-dimensional TAS maps auto-scaled to maximum intensity (*λ*_ex_ = 355 nm, 0.5 mJ) for a solution of ACR-IMAC (0.2 mM in degassed MeCN) without added indole 10a, and with added 50 mM indole 10a. (B) Transient absorption temporal profiles for samples of ACR-IMAC (0.2 mM) with concentrations of indole 10a ranging between 0 and 50 mM, monitored over the range of 380–480 nm, with amplitude-weighted lifetimes shown inset. (C) Individual lifetimes *τ*_1_ and *τ*_2_ from biexponential fits to temporal profiles shown in (B), and an amplitude-weighted average lifetime of *τ*_1_ and *τ*_2_ (*τ*_avg_) plotted inversely as a function indole 10a concentration.

While nonradiative decay of the excited state of ACR-IMAC dominates in solution, the behaviour of the long-lived T_1_ state can also be probed by microsecond transient absorption spectroscopy (μs-TAS).^45^ Given the similar behaviour in solution of the other IMAC-based PCs presented here, TAS was used to probe their excited state lifetimes using excitation at 355 nm in degassed MeCN (Fig. 5A and S4†). ACR-IMAC (*τ* = 64 μs), CZ-IMAC (*τ* = 20 μs), CZ-Me-IMAC (*τ* = 36 μs), and TerCZ-IMAC (*τ* = 95 μs) all had excited state lifetimes on the same order of magnitude. Conversely, POX-IMAC (*τ* = 1.3 ms) and PTZ-IMAC (*τ* = 1.2 ms) had much longer excited state lifetimes, while the lifetime of TolCZ-IMAC could not be measured due to poor signal-to-noise from its limited solubility. Although these results alone do not provide a clear relationship between transient lifetimes and PC performance, we further explored TAS as a means to characterize the excited state behaviour of ACR-IMAC.

By monitoring the transient absorption profile of the photocatalyst both in the presence and absence of indole substrate, the effect of the substrate on the triplet lifetime of ACR-IMAC could be directly observed. As shown in Fig. 5A and B, transient absorption at 434 nm of a 0.2 mM solution of ACR-IMAC is quenched upon the addition of 10a in increasing concentration ranging from 0 mM (*τ* = 64 μs) to 50 mM (*τ* = 452 ns). Furthermore, plotting the inverse of transient lifetimes as a function indole 10a concentration yields a linear relationship, similar to what one may expect from a fluorescence Stern–Volmer quenching experiment (Fig. 5C). As a control TAS experiment, a solution of ACR-IMAC and 1*H*-indole (*E*_T_ = 71.9 kcal mol^−1^, 50 mM)^46^ was measured; unlike indole 10a, the higher triplet energy 1H-indole substrate is found to not quench the excited state of ACR-IMAC (Fig. S6†). Overall, in conjunction with the high oxidation potential of 10a precluding a SET mechanism, these results collectively support the presence of an EnT mechanism for ACR-IMAC.

## Conclusions

Here we have shown that an imidazoacridine-based TADF material, ACR-IMAC, is a highly effective and general triplet sensitizer that can serve as a replacement for the expensive iridium-based photocatalysts being used for promoting visible-light mediated [2 + 2] cycloadditions. The product conversion of this organo-photocatalyst matched the iridium-based photocatalysts in dearomative and non-dearomative intra- and intermolecular [2 + 2] cycloadditions. ACR-IMAC is readily synthesized in gram quantities from inexpensive, commercially available starting materials and is a bench-stable white solid. The ability of ACR-IMAC to perform well in MeCN, a commonly used solvent for other photocatalyzed reactions, including cross-couplings, indicates that its applications could extend beyond [2 + 2] cycloadditions. Detailed studies of the reaction mechanism by Stern–Volmer analyses of luminescence quenching experiments and microsecond time-resolved transient absorption spectroscopy (μs-TAS) are further suggestive of a triplet–triplet EnT sensitization process. We believe that the presented work will encourage the broader use and development of TADF materials as organo-photocatalysts, particularly for challenging, high-energy chemical transformations mediated by visible-light. Other potential uses of ACR-IMAC and related materials as organo-photocatalysts in organic synthesis are also being actively sought in our groups.

## Author contributions

ERS, MSO and ZMH conceived and designed the project. MSO performed all of the photocatalyzed cycloaddition reactions. ERS synthesized the photocatcatalysts and performed the fluorescence measurements. DMM and SK performed the transient absorption spectroscopy. ERS, MSO and ZMH wrote the manuscript with input from all authors. All authors contributed to the interpretation of results.

## Conflicts of interest

There are no conflicts to declare.

## Supplementary Material

SC-013-D1SC05098B-s001

## References

[cit1] Prier C. K., Rankic D. A., MacMillan D. W. C. (2013). Chem. Rev..

[cit2] Romero N. A., Nicewicz D. A. (2016). Chem. Rev..

[cit3] Ravelli D., Dondi D., Fagnoni M., Albini A. (2009). Chem. Soc. Rev..

[cit4] Marzo L., Pagire S. K., Reiser O., König B. (2018). Angew. Chem., Int. Ed..

[cit5] Zhou Q., Zou Y., Lu L., Xiao W. (2019). Angew. Chem., Int. Ed..

[cit6] Strieth-Kalthoff F., Glorius F. (2020). Chem.

[cit7] Strieth-Kalthoff F., James M. J., Teders M., Pitzer L., Glorius F. (2018). Chem. Soc. Rev..

[cit8] Zhao J., Wu W., Sun J., Guo S. (2013). Chem. Soc. Rev..

[cit9] Tucker J. W., Stephenson C. R. J. (2012). J. Org. Chem..

[cit10] Monos T. M. and Stephenson C. R. J., in Iridium(III) in Optoelectronic and Photonics Applications, John Wiley & Sons, Ltd, Chichester, UK, 2017, pp. 541–581

[cit11] Oderinde M. S., Jin S., Dhar T. G. M., Meanwell N. A., Mathur A., Kempson J. (2021). Tetrahedron.

[cit12] Büldt L. A., Wenger O. S. (2017). Chem. Sci..

[cit13] Larsen C. B., Wenger O. S. (2018). Chem. –Eur. J..

[cit14] Chen J.-R., Hu X.-Q., Lu L.-Q., Xiao W.-J. (2016). Acc. Chem. Res..

[cit15] Vega-Peñaloza A., Mateos J., Companyó X., Escudero-Casao M., Dell'Amico L. (2020). Angew. Chem., Int. Ed..

[cit16] McCarthy B. G., Pearson R. M., Lim C.-H., Sartor S. M., Damrauer N. H., Miyake G. M. (2018). J. Am. Chem. Soc..

[cit17] Theriot J. C., Lim C.-H., Yang H., Ryan M. D., Musgrave C. B., Miyake G. M. (2016). Science.

[cit18] Pearson R. M., Lim C.-H., McCarthy B. G., Musgrave C. B., Miyake G. M. (2016). J. Am. Chem. Soc..

[cit19] Buss B. L., Lim C. H., Miyake G. M. (2020). Angew. Chem., Int. Ed..

[cit20] MacKenzie I. A., Wang L., Onuska N. P. R., Williams O. F., Begam K., Moran A. M., Dunietz B. D., Nicewicz D. A. (2020). Nature.

[cit21] Lu J., Pattengale B., Liu Q., Yang S., Shi W., Li S., Huang J., Zhang J. (2018). J. Am. Chem. Soc..

[cit22] Bryden M. A., Zysman-Colman E. (2021). Chem. Soc. Rev..

[cit23] Rolka A. B., Koenig B. (2020). Org. Lett..

[cit24] Uoyama H., Goushi K., Shizu K., Nomura H., Adachi C. (2012). Nature.

[cit25] Wong M. Y., Zysman-Colman E. (2017). Adv. Mater..

[cit26] Yang Z., Mao Z., Xie Z., Zhang Y., Liu S., Zhao J., Xu J., Chi Z., Aldred M. P. (2017). Chem. Soc. Rev..

[cit27] Nakanotani H., Furukawa T., Hosokai T., Hatakeyama T., Adachi C. (2017). Adv. Opt. Mater..

[cit28] Kaji H., Suzuki H., Fukushima T., Shizu K., Suzuki K., Kubo S., Komino T., Oiwa H., Suzuki F., Wakamiya A., Murata Y., Adachi C. (2015). Nat. Commun..

[cit29] Luo J., Zhang J. (2016). ACS Catal..

[cit30] Phelan J. P., Lang S. B., Sim J., Berritt S., Peat A. J., Billings K., Fan L., Molander G. A. (2019). J. Am. Chem. Soc..

[cit31] Huang H., Yu C., Zhang Y., Zhang Y., Mariano P. S., Wang W. (2017). J. Am. Chem. Soc..

[cit32] Wang Y., Gao X.-W., Li J., Chao D. (2020). Chem. Commun..

[cit33] Zhang Z., Chen W., Zhang Y., Wang Y., Tian Y., Fang L., Ba X. (2021). Macromolecules.

[cit34] Sauvé E. R., Paeng J., Yamaguchi S., Hudson Z. M. (2020). J. Org. Chem..

[cit35] Poplata S., Tröster A., Zou Y.-Q., Bach T. (2016). Chem. Rev..

[cit36] Taylor A. P., Robinson R. P., Fobian Y. M., Blakemore D. C., Jones L. H., Fadeyi O. (2016). Org. Biomol. Chem..

[cit37] Zheng C., You S.-L. (2019). Nat. Prod. Rep..

[cit38] Hurtley A. E., Lu Z., Yoon T. P. (2014). Angew. Chem., Int. Ed..

[cit39] Hu N., Jung H., Zheng Y., Lee J., Zhang L., Ullah Z., Xie X., Harms K., Baik M.-H, Meggers E. (2018). Angew. Chem., Int. Ed..

[cit40] Daub M. E., Jung H., Lee B. J., Won J., Baik M. H., Yoon T. P. (2019). J. Am. Chem. Soc..

[cit41] Zheng J., Swords W. B., Jung H., Skubi K. L., Kidd J. B., Meyer G. J., Baik M. H., Yoon T. P. (2019). J. Am. Chem. Soc..

[cit42] Oderinde M. S., Kempson J., Smith D., Meanwell N. A., Mao E., Pawluczyk J., Vetrichelvan M., Pitchai M., Karmakar A., Rampulla R., Li J., Murali Dhar T. G., Mathur A. (2020). European J. Org. Chem..

[cit43] Oderinde M. S., Mao E., Ramirez A., Pawluczyk J., Jorge C., Cornelius L. A. M., Kempson J., Vetrichelvan M., Pitchai M., Gupta A., Gupta A. K., Meanwell N. A., Mathur A., Dhar T. G. M. (2020). J. Am. Chem. Soc..

[cit44] Speckmeier E., Fischer T. G., Zeitler K. (2018). J. Am. Chem. Soc..

[cit45] Teders M., Henkel C., Anhäuser L., Strieth-Kalthoff F., Gómez-Suárez A., Kleinmans R., Kahnt A., Rentmeister A., Guldi D., Glorius F. (2018). Nat. Chem..

[cit46] Ahrens L. H. (1951). Appl. Spectrosc..

