# Supplementary material for: An imidazoacridine-based TADF material as an effective organic photosensitizer for visible-light-promoted [2 + 2] cycloaddition
Source: Chem Sci. 2022 Jan 27;13(8):2296–302. doi: 10.1039/d1sc05098b (PMC8864701; doi:10.1039/d1sc05098b)
Supplement: SC-013-D1SC05098B-s001 [file SC-013-D1SC05098B-s001.pdf]

Electronic Supplementary Material (ESI) for Chemical Science.
This journal is © The Royal Society of Chemistry 2022

Supporting Information

An Imidazoacridine-Based TADF Material as an Effective Organic Photosensitizer for Visible-Light-Promoted [2+2] Cycloaddition

Ethan R. Sauvé[a], Don M. Mayder[a], Saeid Kamal,[a] Martins S. Oderinde[b*], and Zachary M. Hudson[a]*

[a]*Department of Chemistry, University of British Columbia, 2036 Main Mall, Vancouver, British Columbia, Canada, V6T 1Z1*

bDepartment of Discovery Synthesis, Bristol Myers Squibb Research and Early Development, 3551 Lawrenceville Road, Princeton, New Jersey, United States of America, 08540

Tel: +1-609-252-5237
*Email: *martin.oderinde@bms.com;*

Tel: +1-604-822-3266; Fax: +1-604-822-2847
*Email: *zhudson@chem.ubc.ca*

Table of Contents

Transient Absorption Spectroscopy 5

Stern-Volmer Quenching 5

Experimental Details 10

General Considerations: 11

Synthesis of CZ-Me-CHO: 11

Synthesis of CZ-Me-IMAC: 12

Photocatalyzed Cycloaddition Reactions: 13

General Procedure A for [2+2] photocycloaddition: Intramolecular [2+2] cycloaddition. 13

General Procedure B for [2+2] photocycloaddition: Intermolecular [2+2] cycloaddition. 13

Compound 4 14

Compound 6a 14

Compound 6b 15

Compound 6c 15

Compound 6d 16

Compound 6e 16

Compound 6f 17

Compound 7 17

Compound 9a 18

Compound 9b 18

Compound 9c 18

Compound 12a 19

Compound 12b 19

Compound 12c 20

Compound 12d 20

Compound 12e 20

Compound 12f 21

Compound 12g 21

Compound 12h 21

Compound 12i 22

Compound 12j 22

Compound 12k 23

^{1}H NMR (400 MHz) of CZ-Me-CHO in methylene chloride-d_2 24

$^{13}C\{^{1}H\}$ NMR (101 MHz) of CZ-Me-CHO in methylene chloride-d_2 24

^{1}H NMR (400 MHz) of CZ-Me-IMAC in methylene chloride-d_2 25

$^{13}C\{^{1}H\}$ NMR (101 MHz) of CZ-Me-IMAC in methylene chloride-d_2 25

^{1}H-NMR of Spectrum Compound 4 26
^{13}C-NMR of Spectrum Compound 4 26
^{1}H-NMR of Spectrum Compound 6a 27
^{13}C-NMR of Spectrum Compound 6a 27
^{1}H-NMR of Spectrum Compound 6b 28
^{13}C-NMR of Spectrum Compound 6b 28
^{19}F NMR Spectrum of 6b 29
^{1}H-NMR of Spectrum Compound 6c 30
^{13}C-NMR of Spectrum Compound 6c 30
^{19}F-NMR of Spectrum Compound 6c 31
^{1}H-NMR of Spectrum Compound 6d 32
^{13}C-NMR of Spectrum Compound 6d 32
^{1}H-NMR of Spectrum Compound 6e 33
^{13}C-NMR of Spectrum Compound 6e 33
^{1}H-NMR of Spectrum Compound 6f 34
^{13}C-NMR of Spectrum Compound 6f 34
Compound 7 35
1D proton spectrum of 7 in $CDCl_3$ at 27 ℃ 36
1D carbon spectrum of compound 7 in $CDCl_3$ at 27 ℃ 36
2D ^{13}C-HSQC carbon spectrum of compound 7 in $CDCl_3$ at 27 ℃ 37
2D ^{1}H-^{1}H-NOESY carbon spectrum of compound 7 in $CDCl_3$ at 27 ℃ 37
Chemical Shifts* and Coupling Constants of compound 7 in $CDCl_3$ at 27 ℃ 38
NOESY Table for compound 7 in $CDCl_3$ at 27℃. 39
^{1}H-NMR of Spectrum Compound 9a 40
^{13}C-NMR of Spectrum Compound 9a 40
^{1}H-NMR of Spectrum Compound 9b 41
^{13}C-NMR of Spectrum Compound 9b 41
^{1}H-NMR of Spectrum Compound 9c 42
^{13}C-NMR of Spectrum Compound 9c 42
^{1}H-NMR Spectrum of Compound 12a 43
^{13}C-NMR Spectrum of Compound 12a 44
^{19}F-NMR Spectrum of Compound 12a 45
^{1}H-NMR spectrum of 12b 46
^{13}C-NMR spectrum of 12b 47
2D Elucidation of Compound 12c 48

1D proton spectrum of 12c in DMSO-d6 at 27 ℃ 49
1D carbon spectrum of 12c in DMSO-d6 at 27 ℃ 49
^{1}H-^{13}C DEPT-HSQC spectrum of 12c in DMSO-d6 at 27 ℃ 50
^{1}H-^{1}H NOESY spectrum of 12c in DMSO-d6 at 27 ℃ 50
Chemical Shifts and Coupling Constants of 12c in DMSO-d6 at 27 ℃* 51
NOESY table for 12c in DMSO-d6 at 27 ℃ 52
^{1}H-NMR spectrum of 12d 53
^{13}C-NMR spectrum of 12d 54
^{1}H-NMR spectrum of 12e 55
^{13}C-NMR spectrum of 12e 56
^{1}H-NMR spectrum of 12f 57
^{13}C-NMR spectrum of 12f 58
^{1}H-NMR spectrum of 12g 59
^{13}C-NMR spectrum of 12g 60
^{1}H-NMR spectrum of 12h 61
^{13}C-NMR spectrum of 12h 62
^{1}H-NMR spectrum of 12i 63
^{13}C-NMR spectrum of 12i 64
^{1}H-NMR spectrum of 12j 65
^{13}C-NMR spectrum of 12j 66
^{1}H-NMR spectrum of 12k 67
^{13}C-NMR spectrum of 12k 68

Transient Absorption Spectroscopy: Measurements were performed using a streak camera system (C7700, Hamamatsu Photonics), CCD camera (C8484, Hamamatsu Photonics), and spectrograph (SP2300i, Princeton Instruments). The excitation pulses were generated at 355 nm by a Nd:YAG picosecond laser (PL2241, EKSPLA), with 35 ps pulse duration, 0.5 to 2.0 mJ pulse energy, and operating at 10 Hz. Probe light was obtained from a CW xenon lamp (L8004, Hamamatsu Photonics).

Transient absorption quenching experiment samples were prepared in a nitrogen glovebox, using freeze-pump-thawed acetonitrile. **ACR-IMAC** was dissolved in MeCN (0.2 mM), and this **ACR-IMAC/MeCN** solution was used to prepare a stock solution of indole **10a** (0.05 M). The combined **ACR-IMAC/10a** and **ACR-IMAC** stock solutions were combined in different ratios to achieve concentrations of **10a** ranging from 0 to 50 mM. Each sample (3 mL) was transferred to a quartz cuvette, and rigorously sealed using a rubber o-ring cap. Samples were then promptly measured, using the aforementioned instrument settings.

Samples of other IMAC photocatalysts for TAS were prepared in acetonitrile, with concentrations adjusted for optimal emission intensity and absorption at 355 nm. Each sample was syringe-filtered with a 0.2 μm PTFE filter, then vigorously sparged with nitrogen gas for 10 minutes, and held under an atmosphere of nitrogen gas throughout the duration of the TA measurement.

Stern-Volmer Quenching: A stock solution of photocatalyst (0.2 mM, either **ACR-IMAC** or **Ir-1**) was prepared in acetonitrile. The photocatalyst stock solution was then used to separately dissolve indole **10a** to yield a photocatalyst/indole **10a** combined stock solution ($C_{photocatalyst}$ = 0.2 mM, C_{10a} = 0.05 M). Five solutions (3.5 mL each) were prepared with each of the two stock solutions, such that the concentration of photocatalyst was held constant (0.2 mM) while the concentration of **10a** ranged from 0 to 0.050 M. Each of the five solutions were sparged with nitrogen for five minutes prior to measuring the absorbance and emission spectra (λ_{ex} = 400 nm) of the samples. Inner filter effect corrections of the emission spectra were performed according to a standard procedure.[1]

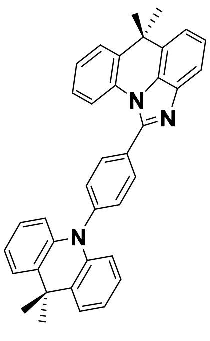

ACR-IMAC

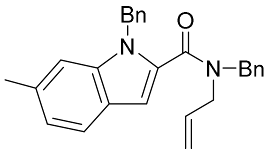

5d

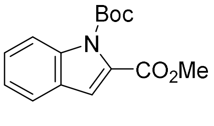

10a

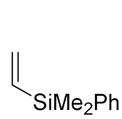

dimethylphenylvinylsilane

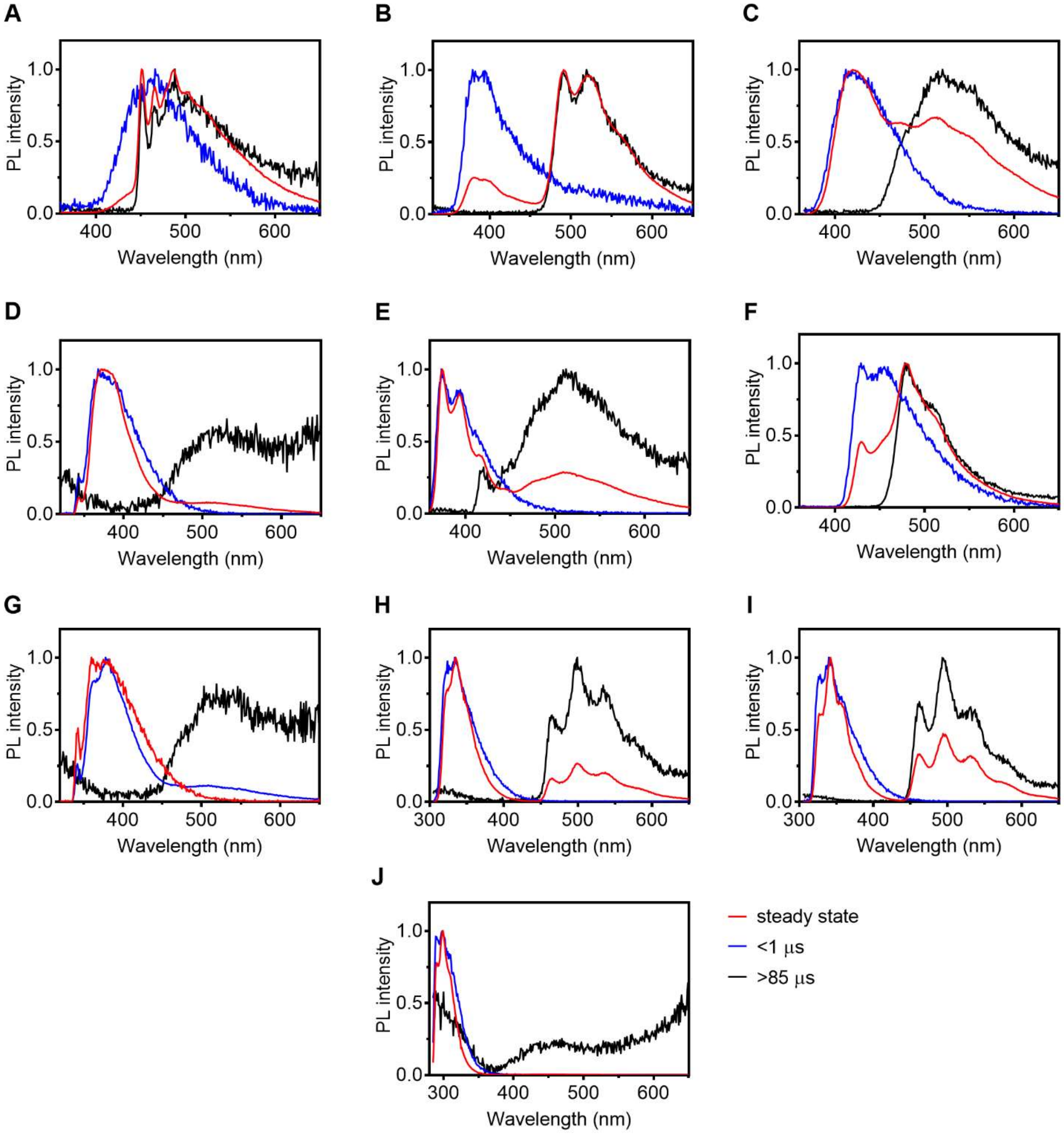

Figure S1: Time-resolved and steady state emission spectra in 2-methyltetrahydrofuran (0.01 mg mL^{-1}) at 77 K, where (A) is **PXZ-IMAC**, (B) is **PTZ-IMAC**, (C) is **ACR-IMAC**, (D) is **CZ-IMAC**, (E) is **TerCZ-IMAC**, (F) is **TolCZ-IMAC**, (G) is **CZ-Me-IMAC**, (H) is **10a**, (I) is benzyl indole **5d**, and (J) is dimethylphenylvinylsilane. Excitation wavelengths: λ_{ex} = 310 nm for A-B, D-F; λ_{ex} = 355 nm for C; λ_{ex} = 300 nm for H-I; λ_{ex} = 280 nm for J.

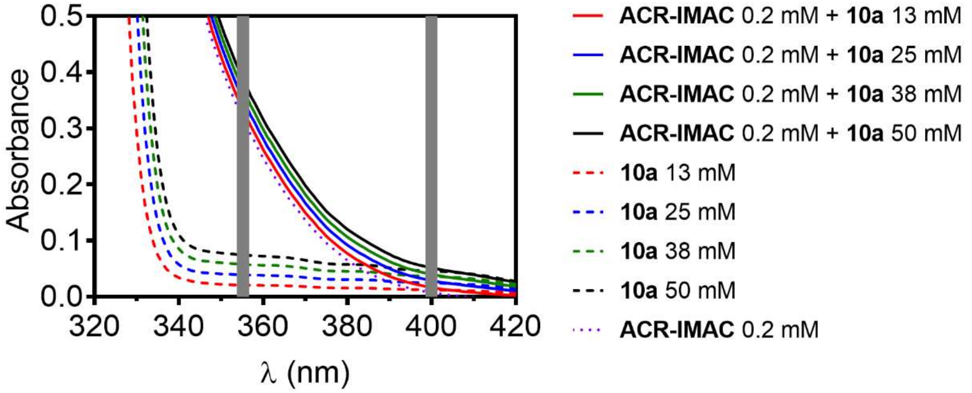

Figure S2: Absorption spectra for MeCN solutions containing combinations of **ACR-IMAC** and indole **10a** (solid lines), or solutions containing only indole **10a** or only **ACR-IMAC** (dashed lines). Vertical grey lines are shown to indicate absorption at wavelengths of 355 nm and 400 nm.

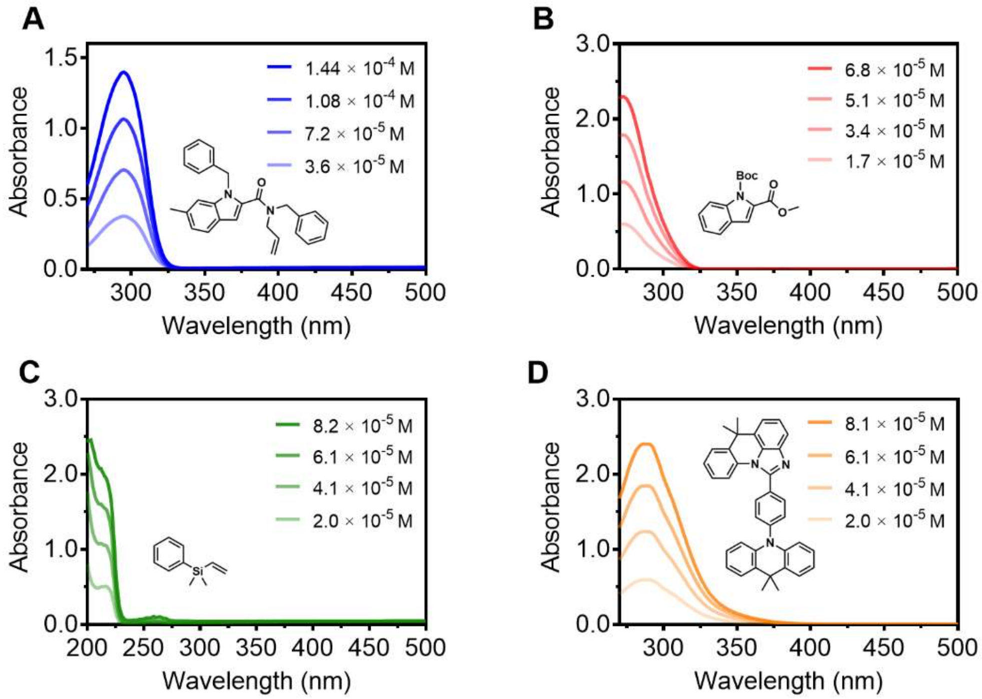

Figure S3: Absorption spectra for MeCN solutions containing (A) benzyl indole **5d**; (B) indole **10a;** (C) dimethylphenylvinylsilane and (D) **ACR-IMAC**.

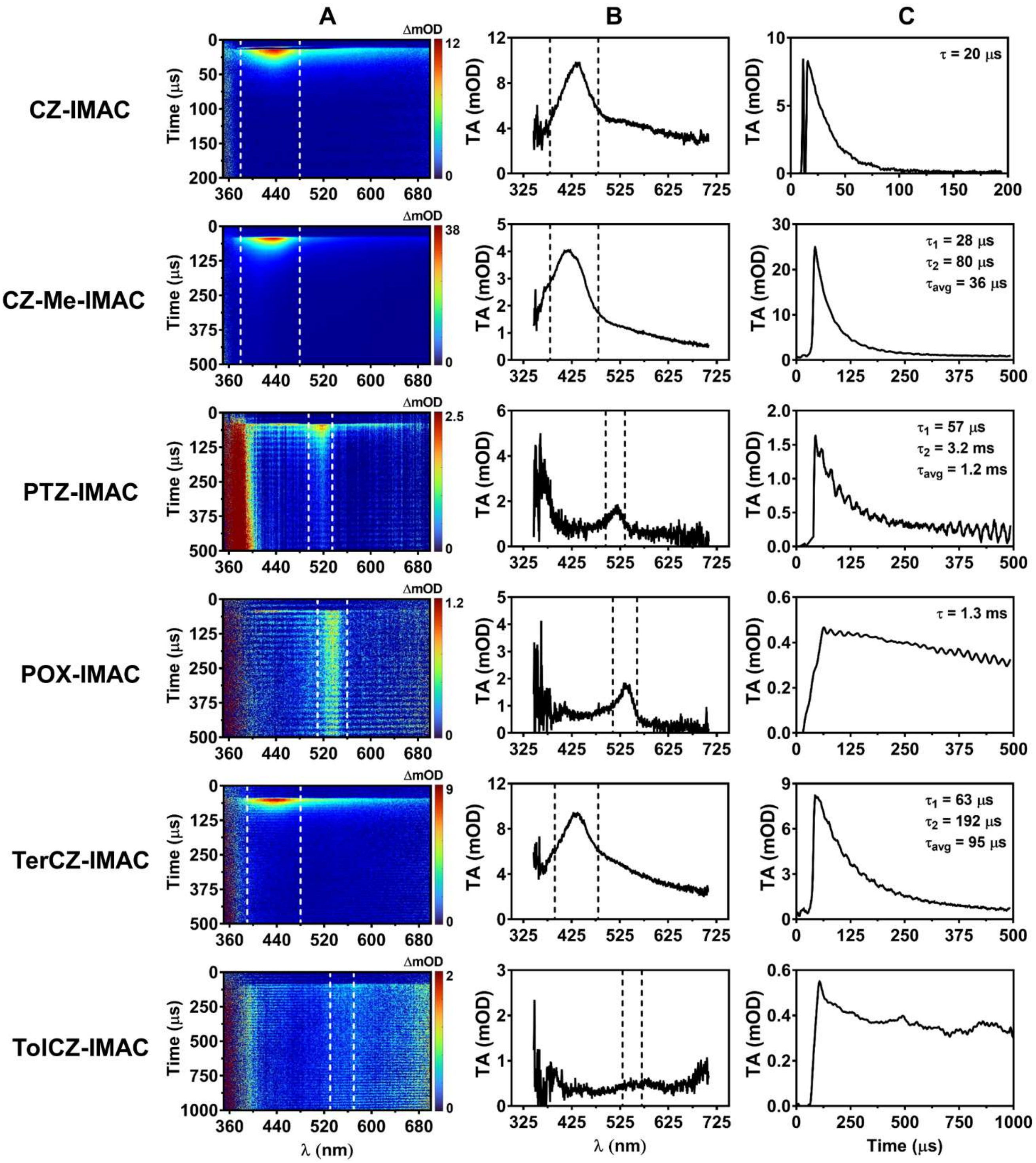

Figure S4: (A) TAS maps of various photocatalysts, with each image auto-scaled accordingly, and sampled spectral boundaries indicated by white dashed lines. (B) Corresponding spectral profiles with sampled spectral boundaries indicated by black dashed lines. (C) Temporal profiles of the sampled range with inset lifetime fitting data shown in each instance where a lifetime analysis was performed.

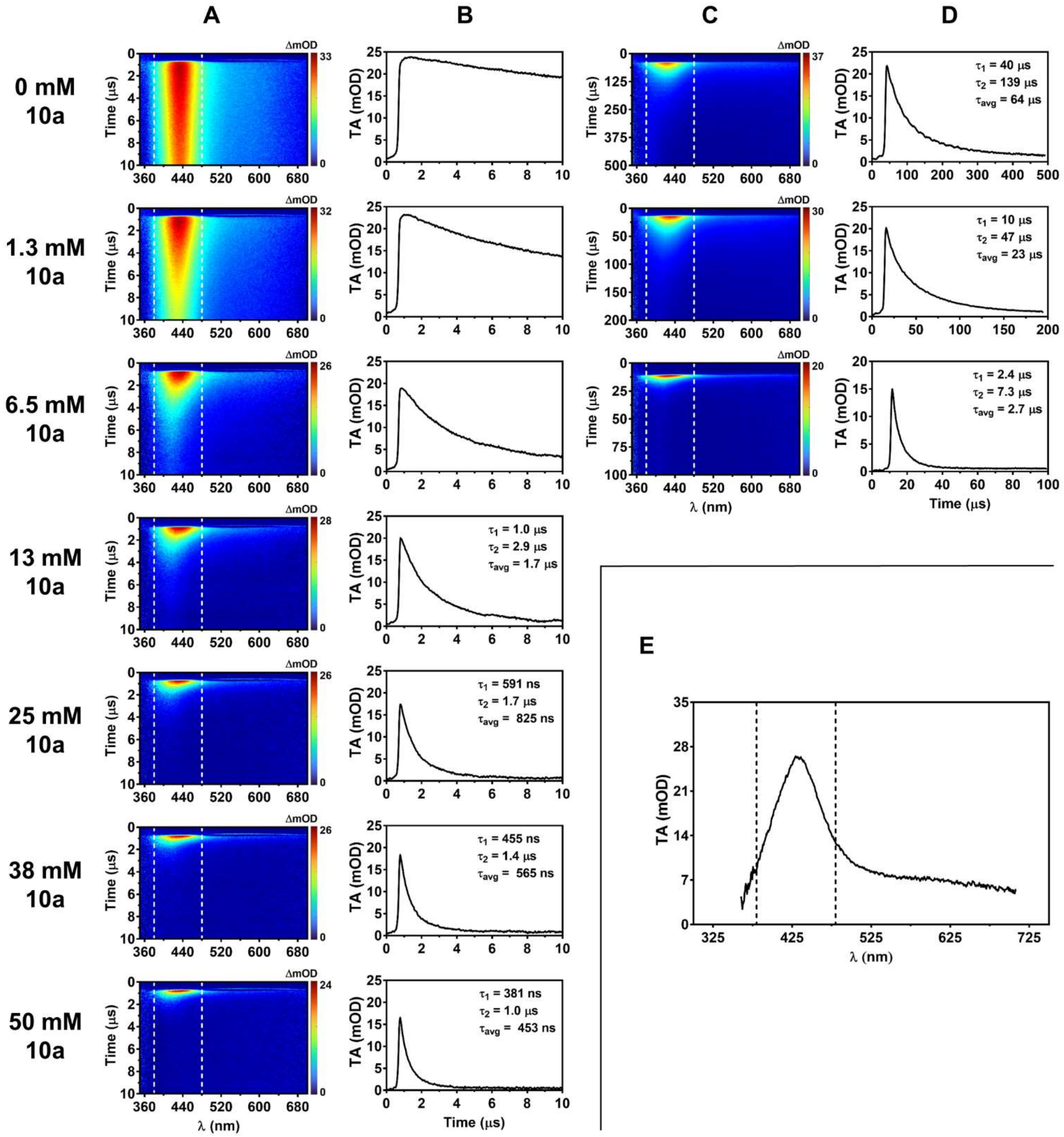

Figure S5: TAS maps of **ACR-IMAC** with varying concentrations of indole **10a** on (A) 10 μs or (C) 100 to 500 μs timescales, with each image auto-scaled accordingly. Corresponding temporal profiles for (B) 10 μs or (D) 100 to 500 μs timescales using identical spectral boundaries (380 to 480 nm) indicated with white dashed lines, with inset biexponential fitting data shown in each instance where a lifetime analysis was performed. (E) A representative spectral profile of **ACR-IMAC** with 0 mM **10a**, with the sampled spectral region shown by black dashed lines.

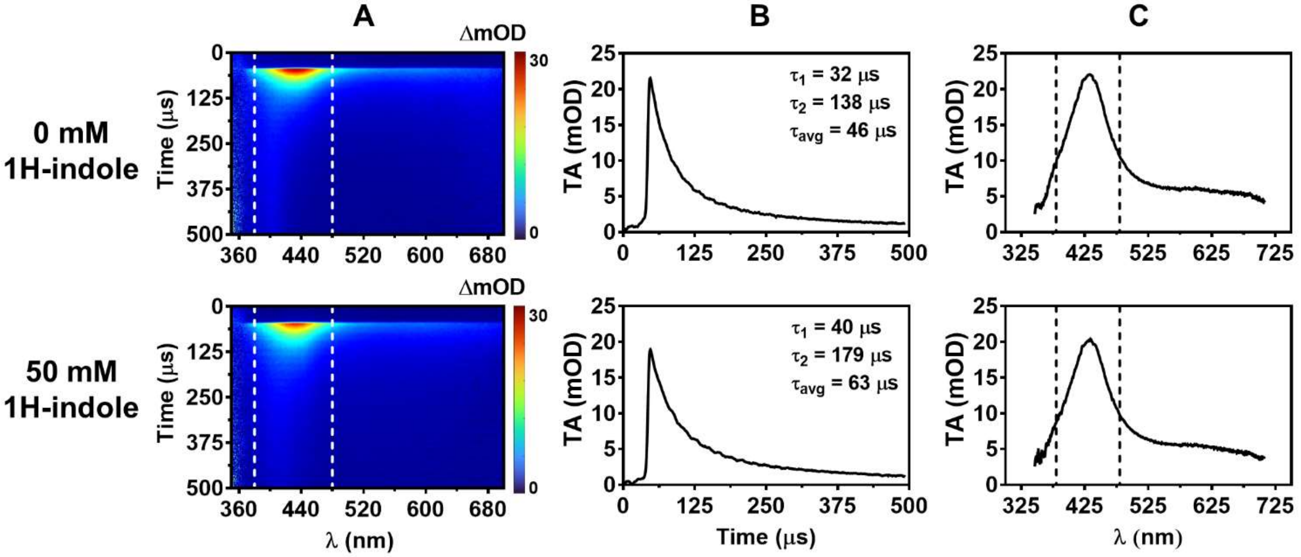

Figure S6: Auto-scaled TAS maps with sampled spectral boundaries (380 to 480 nm) indicated by white dashed lines (A), temporal profiles (B), and emission profiles (C) with spectral boundaries (380 to 480 nm) indicated by black dashed lines, of degassed MeCN solutions containing **ACR-IMAC** with 0 mM or 50 mM 1H-indole.

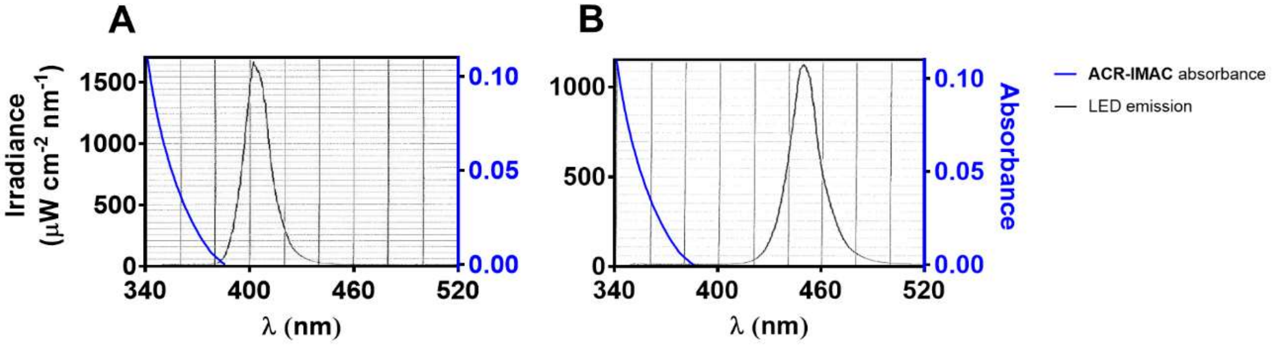

Figure S7. The LED emission profiles (black) for (A) 400 nm excitation and (B) 450 nm excitation, as measured by Hepatochem. The absorbance of **ACR-IMAC** (blue) in acetonitrile is shown overlaid with each LED emission profile.

Experimental Details

General Considerations: The TADF materials used were prepared following literature procedures.[34] All reagents were purchased from Sigma-Aldrich, Alfa Aesar, and Oakwood Chemical, and were used as received unless otherwise stated. The ^{1}H and $^{13}C\{^{1}H\}$ nuclear magnetic resonance (NMR) spectra were measured on a Bruker AV III HD 400 MHz spectrometer with methylene chloride-d_2 (CD_2Cl_2) as the solvent. Absorbance measurements were made on a Cary 60 spectrometer and fluorescence measurements were made on an Edinburgh Instruments FS5 spectrofluorometer; acetonitrile was used as the solvent. Time-correlated single-photon-counting measurements were made with a 313 nm picosecond-pulsed diode LED (Edinburgh Instruments) excitation source, and fit using either mono- or biexponential decay functions. Time-resolved and steady state emission spectra at 77 K were measured in a glassy matrix of 2-methyltetrahydrofuran (0.01 mg mL^{-1}) using virtual gating, with excitation at the absorption maximum for each sample. Triplet energies were measured using steady-state phosphorescence spectra at 77 K. Mass spectra were recorded on a Kratos Concept IIHQ instrument using field desorption (FD) ionization, or on a Bruker HCT ultra PTM Discovery System using electrospray ionization (ESI).

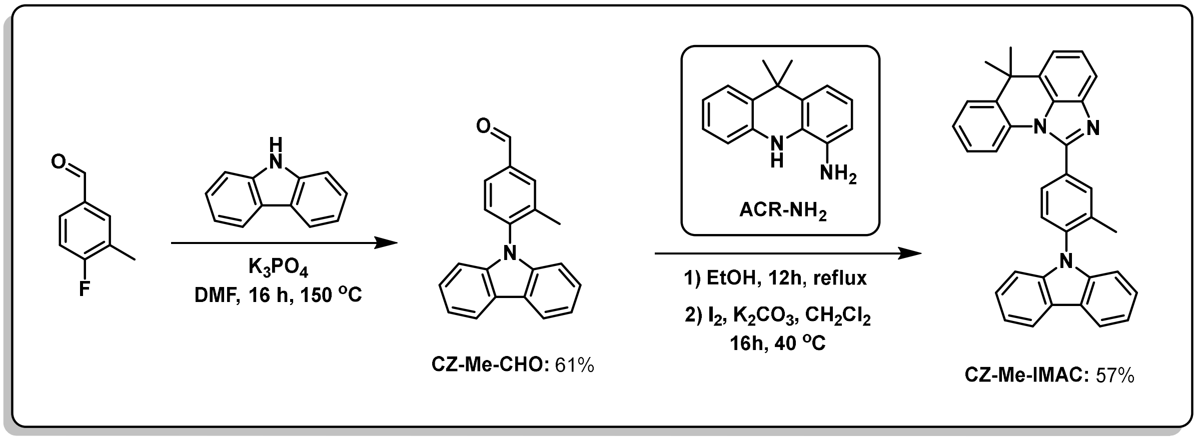

Scheme S1: Synthesis of **CZ-Me-IMAC**.

Synthesis of CZ-Me-CHO: Prepared according to a modified literature procedure.[3] A mixture of 9*H*-carbazole (1.00 g, 5.91 mmol, 1.0 eq), 4-fluoro-3-methylbenzaldehyde (1.44 mL, 11.8 mmol, 2.0 eq), K_3PO_4 (6.40 g, 29.5 mmol, 5.0 eq), and DMF (15 mL) were combined under air in a round bottom flask, and heated for 16 h to 150 °C using an oil bath. Upon completion of the reaction as monitored by TLC, the mixture was vacuum filtered, and the filtrate was concentrated *in vacuo* to afford a crude residue. The

crude product was purified over silica (first column: 1:1, hexanes:CH_2Cl_2; second column: 4:1, hexanes:CH_2Cl_2) to obtain a white powder. Yield = 1.02 g (61%).

^{1}H NMR (300 MHz, Methylene Chloride-*d*$_2$): 10.12 (s, 1H), 8.18 (d, *J* = 7.6 Hz, 2H), 8.02 (s, 1H), 7.93 (dd, *J* = 8.0, 1.6 Hz, 1H), 7.58 (d, *J* = 8.0 Hz, 1H), 7.41 (td, *J* = 8.3, 7.8, 1.2 Hz, 2H), 7.30 (td, *J* = 8.3, 7.8, 1.2 Hz, 2H), 7.05 (d, *J* = 8.1 Hz, 2H), 2.07 (s, 3H).

^{13}C{^{1}H} NMR (101 MHz, Methylene Chloride-*d*$_2$): δ 192.0, 142.1, 141.2, 138.9, 136.9, 133.4, 130.5, 129.0, 126.7, 123.8, 121.0, 120.6, 110.3, 18.1.

HRMS (FD) m/z: $[M+H]^{+\bullet}$ calcd for $[C_{20}H_{16}NO]^{+\bullet}$, 286.1232; found, 286.1226; difference: -2.03 ppm.

Synthesis of CZ-Me-IMAC: Prepared according to a modified literature procedure.[2] A mixture of 9,9-dimethyl-9,10-dihydroacridin-4-amine ("**ACR-NH$_2$**", 360 mg, 1.59 mmol, 1.0 eq) and 4-(9*H*-carbazol-9-yl)-3-methylbenzaldehyde ("**CZ-Me-CHO**", 500 mg, 1.75 mmol, 1.1 eq) in EtOH (15 mL) was refluxed for 12 h using an oil bath. The solvent was evaporated under reduced pressure to give an orange solid, which was redissolved in CH_2Cl_2 (15 mL), followed by the sequential addition of iodine (485 mg, 1.91 mmol, 1.2 eq) and K_2CO_3 (660 mg, 4.77 mmol, 3.0 eq). The reaction mixture was stirred at 40 °C in an oil bath for another 16 h. Upon completion of the reaction, it was quenched with 5% $Na_2S_2O_3$ (20 mL) and then extracted with CH_2Cl_2 (30 mL × 3). The combined organic layer was washed with brine (30 mL), dried over anhydrous $MgSO_4$, concentrated *in vacuo*, and then purified over silica (5:1, CH_2Cl_2:EtOAc) to obtain a white powder. Yield = 450 mg (57%).

^{1}H NMR (400 MHz, Methylene Chloride-*d*$_2$): δ 8.20 (d, *J* = 7.8 Hz, 2H), 7.97 (d, *J* = 1.4 Hz, 1H), 7.83 (dd, *J* = 8.0, 1.7 Hz, 1H), 7.68 (dd, *J* = 7.9, 1.4 Hz, 1H), 7.63 (dd, *J* = 7.6, 1.1 Hz, 1H), 7.58 (d, *J* = 8.0 Hz, 1H), 7.51 – 7.44 (m, 2H), 7.41 (t, *J* = 7.6 Hz, 1H), 7.39 – 7.29 (m, 4H), 7.25 (td, *J* = 7.7, 1.3 Hz, 1H), 7.20 (d, *J* = 8.1 Hz, 2H), 7.16 (ddd, *J* = 8.6, 7.4, 1.5 Hz, 1H), 2.08 (s, 3H), 1.78 (s, 6H).

^{13}C{^{1}H} NMR (101 MHz, Methylene Chloride-*d*$_2$): δ 150.9, 141.55, 141.52, 138.7, 138.2, 136.9, 134.0, 133.7, 133.4, 132.1, 130.9, 130.2, 129.2, 128.6, 127.3, 126.6, 126.1, 125.1, 123.8, 120.9, 120.4, 119.2, 117.2, 110.4, 38.2, 33.2, 30.3, 18.1.

HRMS (FD) m/z: $[M]^{+\bullet}$ calcd for $[C_{35}H_{27}N_3]^{+\bullet}$, 489.2205; found, 489.2188; difference: -3.45 ppm.

Photocatalyzed Cycloaddition Reactions: The blue and purple flexible LED strips (34 - 72 W) were purchased from Solid Apollo (SKU: SA-LS-BL-5050-300-24V and SA-LS-UVL-5050-300-24V respectively). The vial was placed appropriately 2 cm from the light. All reactions were performed using borosilicate glass vials of appropriate sizes. No light or wavelength filters were used.

General Procedure A for [2+2] photocycloaddition: Intramolecular [2+2] cycloaddition. An 8 mL borosilicate glass vial was charged with **ACR-IMAC** (2 mol%), substrate (0.5 mmol), and anhydrous CH_3CN (0.05 M, 10 mL) then sealed. The reaction mixture was then degassed by sparging with N_2 for 5 minutes. Alternatively, reactions were prepared inside a N_2-filled glove-box with oxygen-free reagents and solvents. The sealed vial containing the reaction mixture was then irradiated in a photoreactor wrapped with purple-LED strips (400 nm, 34 - 50 W), which is equipped with an internal cooling fan to maintain temperature to <28 ^oC for 18 – 24 h. After the completion of the reaction (monitored by LCMS or TLC), the solvent was removed on rotavap under high vacuum and the crude product was purified by silica gel column chromatography using 10-100% EtOAc/hexanes as eluent.

General Procedure B for [2+2] photocycloaddition: Intermolecular [2+2] cycloaddition. An 8 mL borosilicate glass vial was charged with **ACR-IMAC** (2 mol%), substrate (0.5 mmol, 1.0 equiv.), alkene (1.5 mmol, 3.0 equiv.) and anhydrous CH_3CN (0.1 M, 5 mL) then sealed. Alternatively, reactions were prepared inside a N_2-filled glove-box with oxygen-free reagents and solvents. The sealed vial containing the reaction mixture was then irradiated in a photoreactor wrapped with purple-LED strips (400 nm, 34 - 50 W), which is equipped with an internal cooling fan to maintain temperature to <28 ^oC for 18 – 24 h. After the completion of the reaction (monitored by LCMS or TLC), the solvent was removed on rotavap under high vacuum and the crude product was purified by silica gel column chromatography using 10-100% EtOAc/hexanes as eluent.

Compound 4

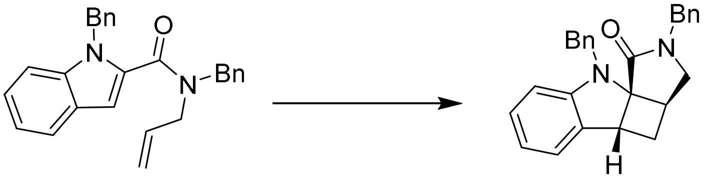

4

Obtained as a white solid (95% yield, *dr* > 99:1) according to the general procedure A.

^{1}H NMR (400 MHz, $CDCl_3$) δ 7.39 - 7.21 (m, 10H), 7.13 - 7.00 (m, 2H), 6.70 (t, *J* = 7.4 Hz, 1H), 6.55 (d, *J* = 7.9 Hz, 1H), 4.57 - 4.40 (m, 3H), 4.18 (d, *J* = 15.0 Hz, 1H), 4.07 (dd, *J* = 8.1, 3.1 Hz, 1H), 3.03 (dd, *J* = 10.3, 7.7 Hz, 1H), 2.92 - 2.85 (m, 1H), 2.78 - 2.70 (m, 1H), 2.39 (ddd, *J* = 12.5, 9.0, 3.4 Hz, 1H), 2.10 (ddd, *J* = 12.6, 8.3, 6.6 Hz, 1H) ppm; ^{13}C NMR (126 MHz, $CDCl_3$) δ 171.5, 164.9, 163.0, 154.4, 154.3, 137.6, 136.0, 128.8, 128.8, 128.6, 128.4, 128.2, 128.1, 127.8, 127.8, 127.8, 127.6, 124.9, 124.8, 104.0, 103.8, 95.6, 95.4, 75.2, 51.6, 48.7, 47.5, 45.6, 34.8, 33.1 ppm; HRMS (ESI) *m/z* $[M+H]^+$ calcd for $C_{26}H_{25}ON_2$ 381.1961, found 381.1953.

Compound 6a

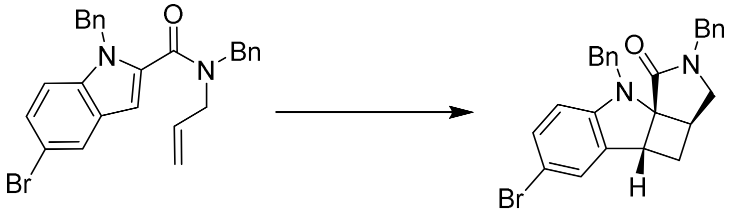

6a

Obtained as a clear oil (80% yield, *dr* > 99:1) according to the general procedure A.

^{1}H NMR (400 MHz, $CDCl_3$) δ 7.39 - 7.16 (m, 11H), 7.10 (dd, *J* = 1.9, 1.0 Hz, 1H), 6.40 (d, *J* = 8.5 Hz, 1H), 4.55 - 4.39 (m, 3H), 4.13 (d, *J* = 15.3 Hz, 1H), 4.04 (dd, *J* = 8.2, 2.9 Hz, 1H), 3.00 (dd, *J* = 10.4, 7.7 Hz, 1H), 2.87 (dd, *J* = 10.4, 1.1 Hz, 1H), 2.74 - 2.67 (m, 1H), 2.38 (ddd, *J* = 12.5, 9.0, 3.5 Hz, 1H), 2.13 - 2.00 (m, 2H) ppm; ^{13}C NMR (126 MHz, $CDCl_3$) δ 171.4, 151.7, 137.7, 136.0, 134.7, 130.9, 128.8, 128.6, 128.4, 128.2, 128.1, 127.9, 127.6, 127.5, 109.5, 108.8, 74.7, 51.6, 48.7, 47.6, 46.0, 34.7, 33.0 ppm; HRMS (ESI) *m/z* $[M+H]^+$ calcd for $C_{26}H_{24}ON_2Br$ 459.1067, found 459.1079.

Compound 6b

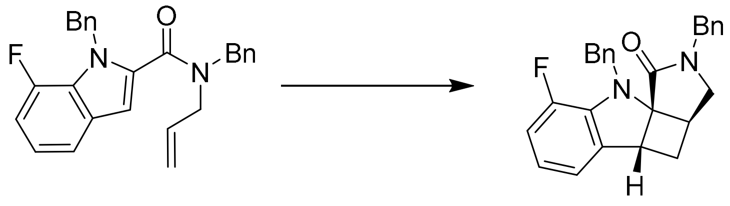

6b

Obtained as a white crystalline solid (95% yield, *dr* > 99:1) according to the general procedure A.

^{1}H NMR (400 MHz, $CDCl_3$) δ 7.40 - 7.14 (m, 10H), 6.91 (dd, *J* = 12.8, 8.1 Hz, 1H), 6.79 (dt, *J* = 7.3, 1.0 Hz, 1H), 6.68 - 6.62 (m, 1H), 4.89 (d, *J* = 15.2 Hz, 1H), 4.47 (s, 2H), 4.29 (d, *J* = 15.4 Hz, 1H), 4.04 (dd, *J* = 8.0, 2.9 Hz, 1H), 2.89 - 2.74 (m, 2H), 2.59 - 2.50 (m, 1H), 2.32 (ddd, *J* = 12.4, 9.1, 3.3 Hz, 1H), 2.03 (ddd, *J* = 12.5, 8.1, 6.4 Hz, 1H) ppm; ^{13}C NMR (126 MHz, $CDCl_3$) δ 171.4, 149.4, 147.5, 138.9, 138.8, 138.7, 136.0, 135.9, 135.9, 128.8, 128.6, 128.4, 128.4, 127.9, 127.5, 120.5, 120.5, 118.9, 118.9, 116.3, 116.2, 74.5, 51.6, 49.8, 49.8, 47.5, 46.9, 46.9, 34.8, 32.8 ppm; ^{19}F NMR (376 MHz, $CDCl_3$) δ -135.76 ppm; HRMS (ESI) *m/z* $[M+H]^+$ calcd for $C_{26}H_{24}FN_2O$ 399.1873, found 399.1890.

Compound 6c

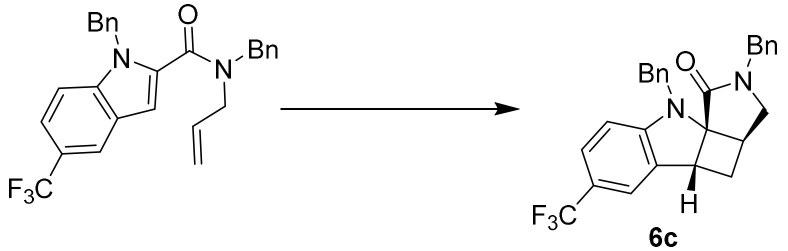

6c

Obtained as a crystalline white solid (97% yield, *dr* > 99:1) according to the general procedure A.

^{1}H NMR (400 MHz, $CDCl_3$) δ 7.41 - 7.18 (m, 12H), 6.55 (d, *J* = 8.4 Hz, 1H), 4.58 - 4.49 (m, 3H), 4.18 (d, *J* = 15.5 Hz, 1H), 4.09 (dd, *J* = 8.1, 3.4 Hz, 1H), 2.97 (dd, *J* = 10.4, 7.8 Hz, 1H), 2.88 (dd, *J* = 10.4, 1.3 Hz, 1H), 2.70 - 2.62 (m, 1H), 2.41 (ddd, *J* = 12.6, 9.0, 3.7 Hz, 1H), 2.13 (ddd, *J* = 12.6, 8.2, 6.1 Hz, 1H) ppm; ^{13}C NMR (126 MHz, $CDCl_3$) δ 171.0, 155.2, 137.4, 135.9, 132.6, 128.9, 128.6, 128.5, 128.1, 127.9, 127.7, 126.4, 126.4, 121.6, 121.6, 106.2, 74.6, 51.6, 48.2, 47.6, 45.9, 35.6, 33.1 ppm; ^{19}F NMR (376 MHz, $CDCl_3$) δ -60.76 ppm; HRMS (ESI) *m/z* $[M+H]^+$ calcd for $C_{27}H_{24}ON_2F_3$ 449.1835, found 449.1831.

Compound 6d

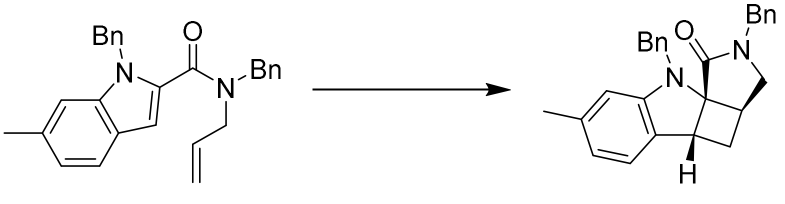

6d

Obtained as an off-white solid (99% yield, *dr* > 99:1) according to the general procedure A.

^{1}H NMR (500 MHz, $CDCl_3$) δ 7.40 - 7.23 (m, 10H), 6.93 (d, J = 7.5 Hz, 1H), 6.55 (d, J = 7.4 Hz, 1H), 6.41 (s, 1H), 4.57 - 4.42 (m, 3H), 4.21 (d, J = 15.4 Hz, 1H), 4.05 (dd, J = 8.0, 2.9 Hz, 1H), 3.03 (dd, J = 10.4, 7.7 Hz, 1H), 2.89 (dd, J = 10.4, 1.1 Hz, 1H), 2.78 - 2.71 (m, 1H), 2.40 - 2.34 (m, 1H), 2.31 (s, 3H), 2.09 (ddd, J = 12.4, 8.1, 6.2 Hz, 1H) ppm; ^{13}C NMR (126 MHz, $CDCl_3$) δ 126.9, 126.6, 126.5, 126.3, 125.9, 125.5, 122.3, 117.0, 106.6, 49.8, 47.0, 45.6, 44.2, 32.5, 31.3, 20.0 ppm; HRMS (ESI) *m/z* $[M+H]^+$ calcd for $C_{27}H_{27}N_2O$ 395.2123, found 3952137.

Compound 6e

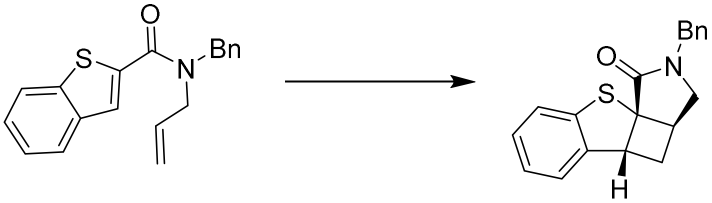

6e

Obtained as a white solid (93% yield, *dr* > 99:1) according to the general procedure A.

^{1}H NMR (400 MHz, $CDCl_3$) δ 7.41 - 7.17 (m, 7H), 7.09 (s, 1H), 7.09 (d, J = 4.8 Hz, 2H), 4.74 (d, J = 14.5 Hz, 1H), 4.51 - 4.41 (m, 2H), 3.54 (dd, J = 10.4, 7.3 Hz, 1H), 3.18 (q, J = 7.6 Hz, 1H), 3.07 (d, J = 10.4 Hz, 1H), 2.55 (ddd, J = 12.3, 8.9, 3.7 Hz, 1H), 2.39 (ddd, J = 12.2, 8.4, 6.6 Hz, 1H) ppm; ^{13}C NMR (125 MHz, $CDCl_3$) δ 172.2, 141.8, 141.7, 136.0, 128.9, 128.3, 128.3, 127.9, 125.1, 124.6, 122.4, 62.6, 54.5, 51.3, 47.7, 40.2, 35.5 ppm; HRMS (ESI) *m/z* $[M+H]^+$ calcd for $C_{19}H_{18}ONS$ 308.1104, found 308.1099.

Compound 6f

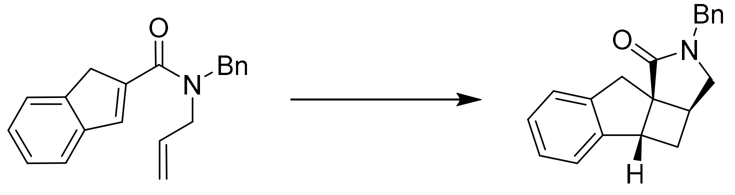

6f

Obtained as a clear oil (98% yield, *dr* > 99:1) according to the general procedure A.

^{1}H NMR (400 MHz, $CDCl_3$) δ 7.41 - 7.18 (m, 9H), 4.73 (d, *J* = 14.6 Hz, 1H), 4.46 (d, *J* = 14.6 Hz, 1H), 3.99 (dd, *J* = 7.1, 4.3 Hz, 1H), 3.60 - 3.45 (m, 2H), 3.15 - 3.08 (m, 1H), 3.02 (d, *J* = 16.4 Hz, 1H), 2.68 (q, *J* = 7.6 Hz, 1H), 2.34 - 2.22 (m, 2H) ppm; ^{13}C NMR (125 MHz, $CDCl_3$) δ 176.7, 146.0, 142.7, 136.6, 128.8, 128.1, 127.6, 127.2, 127.1, 125.1, 124.4, 54.9, 52.1, 49.8, 47.1, 39.7, 34.6, 33.8 ppm; HRMS (ESI) *m/z* $[M+H]^+$ calcd for $C_{20}H_{20}ON$ 290.1539, found 290.1535.

Compound 7

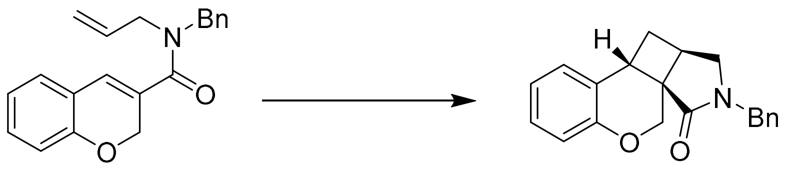

7

Obtained as a white solid (90% yield, *dr* > 99:1) according to the general procedure A.

^{1}H NMR (400 MHz, $CDCl_3$) δ 7.41 - 7.27 (m, 5H), 7.15 (ddd, *J* = 8.3, 7.0, 1.8 Hz, 1H), 7.05 - 6.93 (m, 3H), 4.70 (d, *J* = 14.5 Hz, 1H), 4.43 (d, *J* = 14.5 Hz, 1H), 4.08 - 3.96 (m, 2H), 3.61 (dd, *J* = 8.9, 3.4 Hz, 1H), 3.50 (dd, *J* = 10.3, 7.2 Hz, 1H), 3.12 (d, *J* = 10.3 Hz, 1H), 3.05 - 2.98 (m, 1H), 2.31 (ddd, *J* = 12.3, 8.9, 6.7 Hz, 1H), 1.97 (ddd, *J* = 12.3, 8.9, 3.4 Hz, 1H) ppm; ^{13}C NMR (126 MHz, $CDCl_3$) δ 174.2, 154.1, 136.3, 129.0, 128.9, 128.1, 127.8, 127.3, 122.1, 117.8, 66.1, 51.4, 51.2, 46.9, 35.6, 34.5, 30.2 ppm; HRMS (ESI) *m/z* $[M+H]^+$ calcd for $C_{20}H_{20}O_2N$ 306.1489, found 306.1486.

Compound 9a

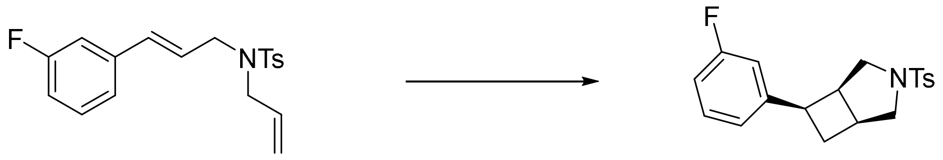

9a

Obtained as a clear oil (84% yield, *dr* > 99:1) according to the general procedure A.

^{1}H NMR (500 MHz, $CDCl_3$) δ 7.68 - 7.64 (m, 2H), 7.34 - 7.30 (m, 3H), 7.02 (d, *J* = 7.7 Hz, 1H), 6.93 (td, *J* = 8.4, 2.1 Hz, 1H), 6.87 (dd, *J* = 10.1, 1.7 Hz, 1H), 3.71 (q, *J* = 9.7 Hz, 1H), 3.47 (d, *J* = 9.5 Hz, 1H), 3.19 (dd, *J* = 10.6, 1.5 Hz, 1H), 3.16 - 3.08 (m, 1H), 2.96 (dt, *J* = 13.5, 7.0 Hz, 1H), 2.72 (dd, *J* = 9.5, 5.4 Hz, 1H), 2.61 (dd, *J* = 10.6, 8.0 Hz, 1H), 2.54 - 2.42 (m, 4H), 2.33 - 2.21 (m, 1H). ^{13}C NMR (126 MHz, $CDCl_3$) δ 164.0, 164.7, 161.8, 162.0, 143.5, 143.5, 132.4, 130.6, 128.0, 123.4, 114.7 (d, *J* = 21.8 Hz), 113.3 (d, *J* = 20.9 Hz), 54.1, 48.5, 42.9, 37.6, 34.2, 29.7, 28.7, 21.5. ^{19}F NMR (376 MHz, $CDCl_3$) δ -113.28. HRMS (ESI) m/z $[M+H]^+$ calcd for $C_{19}H_{20}FNO_2S$ 345.1199 found 346.1262.

Compound 9b

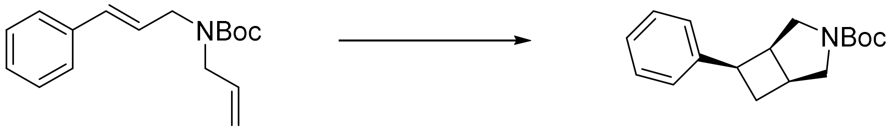

9b

Obtained as a clear oil (82% yield, *dr* > 99:1) according to the general procedure A.

^{1}H NMR (400 MHz, $CDCl_3$) δ 7.37 - 7.30 (m, 2H), 7.28 - 7.17 (m, 3H), 3.85 - 3.55 (m, 2H), 3.52 - 3.40 (m, 1H), 3.36 - 3.21 (m, 2H), 3.06 - 2.88 (m, 2H), 2.45 - 2.29 (m, 1H), 2.25 - 2.11 (m, 1H), 1.57 - 1.50 (m, 9H). ^{13}C NMR (100 MHz, $CDCl_3$) δ 155.3, 145.3, 128.5, 126.4, 126.1, 79.3, 42.7, 32.2, 28.6. HRMS (ESI) m/z $[M+H]^+$ calcd for $C_{17}H_{24}O_2N$ 274.1802, found 274.1802.

Compound 9c

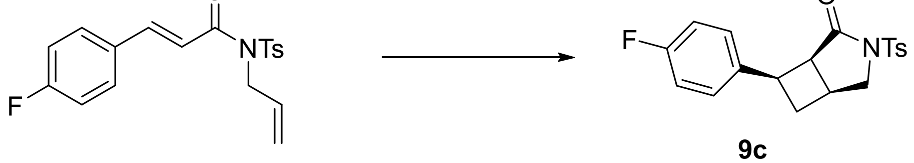

9c

Obtained as a clear oil (25% yield, *dr* > 99:1) according to the general procedure A.

^{1}H NMR (400 MHz, $CDCl_3$) δ 8.00 (d, *J* = 8.3 Hz, 2H), 7.37 (d, *J* = 8.1 Hz, 2H), 7.18 (t, *J* = 6.5 Hz, 2H), 7.01 (t, *J* = 8.3 Hz, 2H), 4.13 - 4.04 (m, 1H), 4.03 - 3.95 (m, 1H), 3.72 - 3.50 (m, 1H), 3.16 - 2.91

(m, 2H), 2.61 - 2.51 (m, 1H), 2.51 - 2.42 (m, 4H). ^{13}C NMR (100 MHz, $CDCl_3$) δ 174.6, 162.8, 160.4, 145.4, 138.8, 138.8, 135.0, 129.8, 128.2, 127.8, 127.7, 115.6, 115.4, 53.4, 49.2, 41.5, 33.2, 26.8, 21.7. ^{19}F NMR (376 MHz, $CDCl_3$) δ -116.06. HRMS (ESI) m/z $[M+H]^+$ calcd for $C_{19}H_{19}O_3NFS$, 362.0976, found 362.0965.

Compound 12a

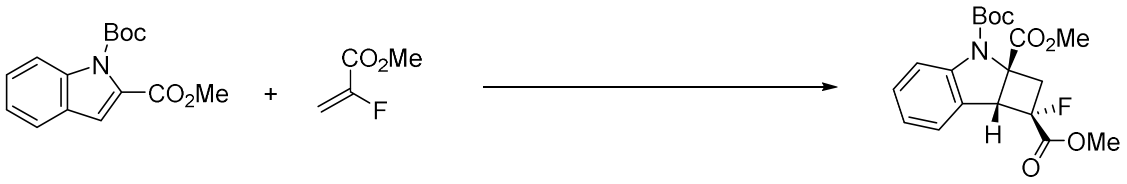

12a

Obtained as an oil (95% yield, crude: >20:1 dr) according to general procedure B.

^{1}H NMR (400 MHz, $CDCl_3$) δ 7.93 (br s, 1H), 7.31 (br t, *J* = 7.7 Hz, 1H), 7.14 (d, *J* = 7.4 Hz, 1H), 7.03 (t, *J* = 7.4 Hz, 1H), 4.61 (dd, *J* = 7.2, 2.6 Hz, 1H), 4.01 - 3.83 (m, 4H), 3.79 (s, 3H), 2.88 - 2.59 (m, 1H), 1.47 (s, 9H); ^{13}C NMR (126 MHz, $CDCl_3$) δ 170.2, 169.5 (*J* = 26.5 Hz), 150.5, 145.3, 129.7, 126.6, 123.3, 115.2, 90.8, 89.0, 82.1, 63.3, 54.5 (*J* = 26.5 Hz) , 53.1 (*J* = 37.5 Hz), 41.5 (*J* = 26.5 Hz), 28.4; ^{19}F NMR (376 MHz, $CDCl_3$) δ -162.52 (s, 1F); LCMS *m/z* $[M+H]^+$ calcd for $C_{19}H_{23}FNO_6$ 380.223, found 380.223.

Compound 12b

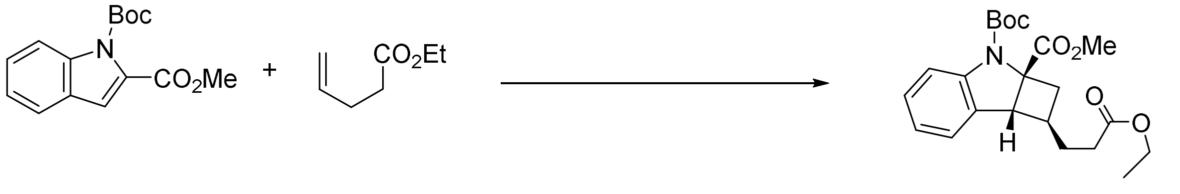

12b

Obtained as an oil (90% yield, crude: 5:1 dr) according to general procedure B.

^{1}H NMR (400 MHz, $CDCl_3$) δ 7.90 (d, *J* = 8.1 Hz, 1H), 7.27 - 7.15 (m, 1H), 7.03 (d, *J* = 7.3 Hz, 1H), 6.95 (t, *J* = 7.4 Hz, 1H), 4.17 - 4.06 (m, 2H), 3.75 - 3.70 (m, 3H), 3.53 (br d, *J* = 5.0 Hz, 1H), 2.90 (br dd, *J* = 13.1, 7.5 Hz, 1H), 2.49 (br dd, *J* = 13.1, 9.3 Hz, 1H), 2.36 - 2.23 (m, 3H), 2.06 - 1.89 (m, 2H), 1.45 (s, 9H), 1.24 (t, *J* = 7.2 Hz, 3H); ^{13}C NMR (100 MHz, $CDCl_3$) δ 172.9, 171.8, 150.9, 144.2, 131.9, 128.1, 123.4, 122.8, 114.8, 81.1, 66.5, 60.4, 52.3, 51.5, 38.7, 34.6, 31.8, 30.7, 28.2, 14.1; HRMS (ESI) *m/z* $[M+H]^+$ calcd for $C_{22}H_{30}NO_6$ 404.2067, found 404.2054.

Compound 12c

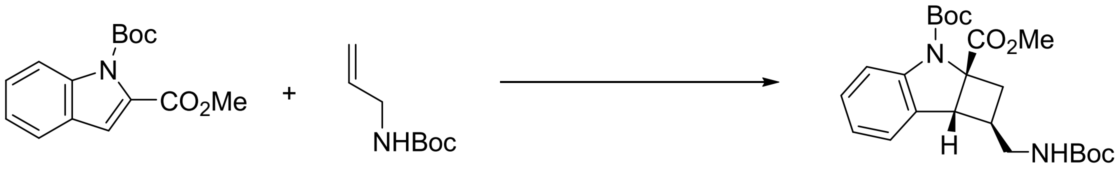

12c

Obtained as an oil (89% yield, crude: 6:1 dr) according to general procedure B.
See 2D NMR section below for full structural elucidation and characterization data.

Compound 12d

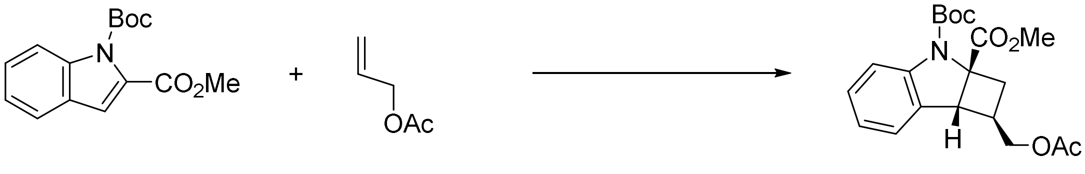

12d

Obtained as an oil (75% yield, crude: 7:1 dr) according to general procedure B.

^{1}H NMR (400 MHz, $CDCl_3$) δ 7.92 (br d, J = 8.0 Hz, 1H), 7.31 - 7.17 (m, 1H), 7.07 - 6.93 (m, 2H), 4.32 - 4.17 (m, 2H), 3.73 (s, 4H), 3.05 (dd, J = 12.8, 7.0 Hz, 1H), 2.64 - 2.53 (m, 1H), 2.53 - 2.43 (m, 1H), 2.11 (s, 3H), 1.46 (s, 9H); ^{13}C NMR (100 MHz, $CDCl_3$) δ 171.4, 170.9, 150.8, 144.2, 131.4, 128.4, 123.5, 122.9, 114.9, 81.3, 66.2, 52.4, 49.2, 37.7, 31.5, 28.2, 20.8; HRMS (ESI) *m/z* $[M+H]^+$ calcd for $C_{20}H_{26}NO_6$ 376.1700, found 376.1741.

Compound 12e

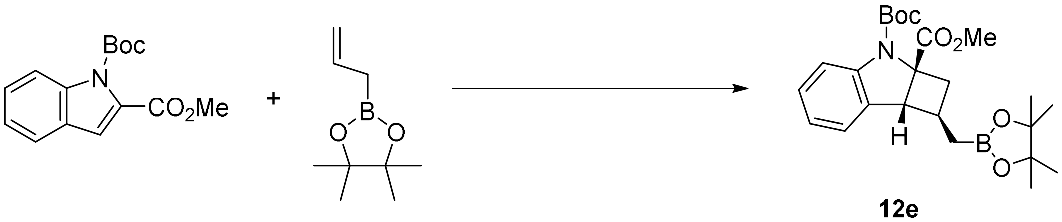

12e

Obtained as an oil (87% yield, crude: 9:1 dr) according to general procedure B.

^{1}H NMR (400 MHz, $CDCl_3$) δ 7.90 (d, J = 8.0 Hz, 1H), 7.24 - 7.12 (m, 2H), 6.94 (br t, J = 7.4 Hz, 1H), 3.75 - 3.70 (m, 3H), 3.53 (br d, J = 5.1 Hz, 1H), 2.85 (br dd, J = 12.8, 7.1 Hz, 1H), 2.54 (br dd, J = 12.8, 9.3 Hz, 1H), 2.48 - 2.27 (m, 1H), 1.45 (s, 9H), 1.31 - 1.14 (m, 14H); ^{13}C NMR (100 MHz, $CDCl_3$) δ 172.1, 151.1, 144.2, 132.7, 127.9, 123.9, 122.5, 114.6, 83.1, 80.8, 66.5, 53.8, 52.2, 37.4, 35.5, 28.2, 24.9, 24.8; HRMS (ESI) *m/z* $[M+H]^+$ calcd for $C_{24}H_{35}BNO_6$ 444.2570, found 444.2573.

Compound 12f

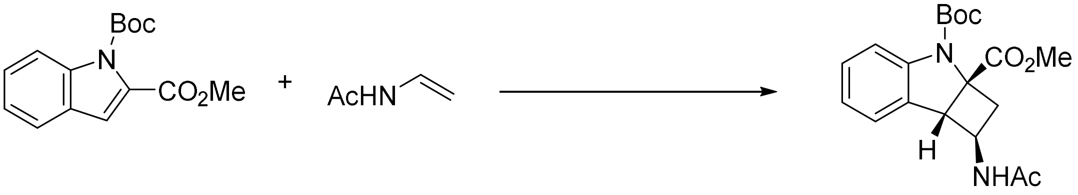

12f

Obtained as an oil (90% yield, crude: 12:1 dr) according to general procedure B.

^{1}H NMR (500 MHz, $CDCl_3$) δ 7.90 (br d, J = 8.0 Hz, 1H), 7.35 - 7.29 (m, 1H), 7.26 - 7.21 (m, 1H), 7.02 (t, J = 7.4 Hz, 1H), 6.00 (br s, 1H), 4.30 - 4.23 (m, 1H), 3.77 (s, 4H), 3.10 (br dd, J = 13.9, 5.1 Hz, 1H), 2.82 (br dd, J = 13.9, 9.2 Hz, 1H), 2.04 (s, 3H), 1.46 (s, 9H); ^{13}C NMR (126 MHz, $CDCl_3$) δ 172.3, 169.7, 150.8, 144.0, 129.3, 128.8, 125.0, 123.4, 115.4, 114.8, 81.4, 65.7, 64.8, 55.8, 52.7, 48.6, 43.3, 36.9, 28.3, 23.2, 23.0; HRMS (ESI) *m/z* $[M+H]^+$ calcd for $C_{19}H_{26}N_2O_5$ 361.4100, found 361.4105.

Compound 12g

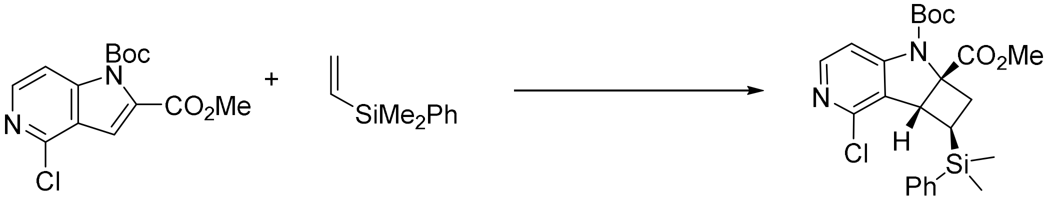

12g

Obtained as an oil (85% yield, crude: 13:1 dr) according to general procedure B.

^{1}H NMR (500 MHz, $CDCl_3$) δ 8.17 (d, J = 5.5 Hz, 1H), 7.56 - 7.53 (m, 2H), 7.41 - 7.28 (m, 3H), 3.84 (d, J = 7.2 Hz, 1H), 3.67 (s, 3H), 3.19 (br dd, J = 12.7, 10.1 Hz, 1H), 2.43 (br t, J = 11.2 Hz, 1H), 2.12 - 2.05 (m, 1H), 1.44 (s, 9H), 0.48 (s, 3H), 0.43 (s, 3H); ^{13}C NMR (100 MHz, $CDCl_3$) δ 169.9, 153.6, 150.2, 150.1, 145.7, 136.5, 133.9, 129.3, 127.8, 127.4, 109.1, 82.7, 69.4, 52.5, 45.3, 30.3, 28.0, 25.6, -4.3, -5.2; HRMS (ESI) *m/z* $[M+H]^+$ calcd for $C_{24}H_{30}ClN_2O_4Si$ 473.1657, found 473.1625.

Compound 12h

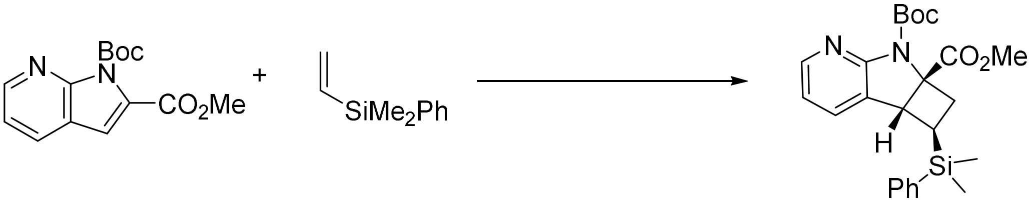

12h

Obtained as an oil (89% yield, crude: 11:1 dr) according to general procedure B.

^{1}H NMR (500 MHz, $CDCl_3$) δ 8.22 (dd, J = 5.1, 1.7 Hz, 1H), 7.50 (d, J = 6.5 Hz, 2H), 7.44 - 7.35 (m, 3H), 6.80 - 6.71 (m, 2H), 3.68 (s, 3H), 3.65 (d, J = 8 Hz, 1H), 3.26 (dd, J = 13.1, 10.1 Hz, 1H), 2.49 (ddd, J = 13.1, 10.8, 0.9 Hz, 1H), 2.01 (td, J = 10.4, 7.7 Hz, 1H), 1.47 (s, 9H), 0.45 (s, 3H), 0.37 (s, 3H); ^{13}C NMR (125 MHz, $CDCl_3$) δ 170.9, 158.2, 149.2, 147.1, 136.8, 133.7, 133.6, 131.1, 129.4, 127.9, 127.5, 117.6, 81.8, 67.7, 52.3, 43.7, 30.1, 28.1, 26.2, -5.0, -5.7; HRMS (ESI) *m/z* $[M+H]^+$ calcd for $C_{24}H_{31}N_2O4Si$ 439.2047, found 439.2037.

Compound 12i

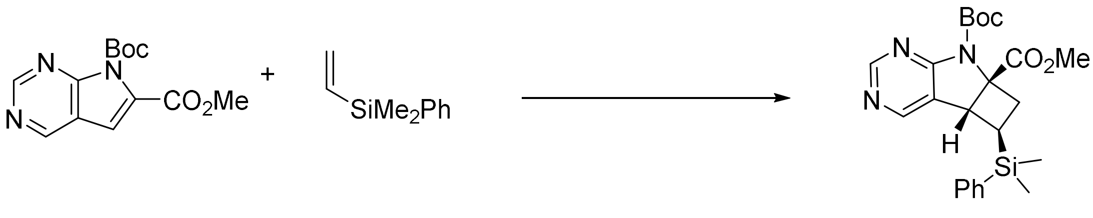

12i

Obtained as an oil (70% yield, crude: 14:1 dr) according to general procedure B.

^{1}H NMR (500 MHz, $CDCl_3$) δ 8.81 (s, 1H), 7.66 (s, 1H), 7.50 (d, J = 6.6 Hz, 2H), 7.45 - 7.38 (m, 3H), 3.70 – 3.67 (m, 4H), 3.28 (dd, J = 13.3, 10.2 Hz, 1H), 2.51 (ddd, J = 13.3, 10.8, 0.9 Hz, 1H), 2.07 - 2.00 (m, 1H), 1.48 (s, 9H), 0.47 (s, 3H), 0.37 (s, 3H); ^{13}C NMR (100 MHz, $CDCl_3$) δ 170.0, 164.5, 157.6, 149.4, 148.5, 136.2, 133.5, 129.7, 128.0, 125.3, 83.1, 68.2, 52.5, 42.1, 30.3, 27.9, 26.3, -5.0, -6.0; HRMS (ESI) *m/z* $[M+H]^+$ calcd for $C_{23}H_{30}N_3O_4Si$ 440.2000, found 440.1991.

Compound 12j

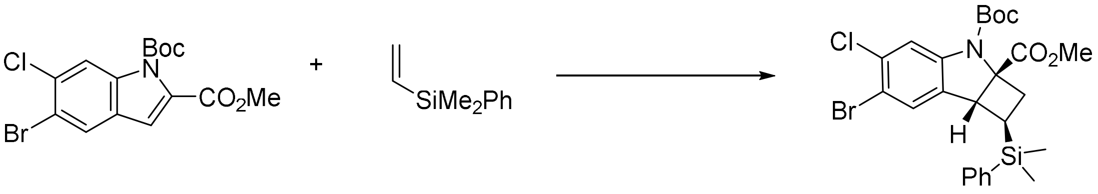

12j

Obtained as an oil (85% yield, crude: 15:1 dr) according to general procedure B.

^{1}H NMR (500 MHz, $CDCl_3$) δ 8.04 (s, 1H), 7.50 (d, J = 6.7 Hz, 2H), 7.46 - 7.37 (m, 3H), 6.59 (s, 1H), 3.68 (s, 3H), 3.63 (d, J = 6.7 Hz, 1H), 3.19 (dd, J = 13.0, 10.1 Hz, 1H), 2.54 - 2.39 (m, 1H), 2.00 (td, J = 10.3, 7.8 Hz, 1H), 1.44 (s, 9H), 0.44 (s, 3H), 0.36 (s, 3H); ^{13}C NMR (126 MHz, $CDCl_3$) δ 170.5, 150.6, 144.8, 136.5, 134.1, 133.7, 133.3, 129.5, 127.9, 127.4, 116.3, 114.5, 81.8, 69.9, 52.3, 45.8, 29.6, 28.1, 26.5, -5.0, -5.8; HRMS (ESI) *m/z* $[M+H]^+$ calcd for $C_{25}H_{30}BrClNO_4Si$ 550.0740, found 550.0752.

Compound 12k

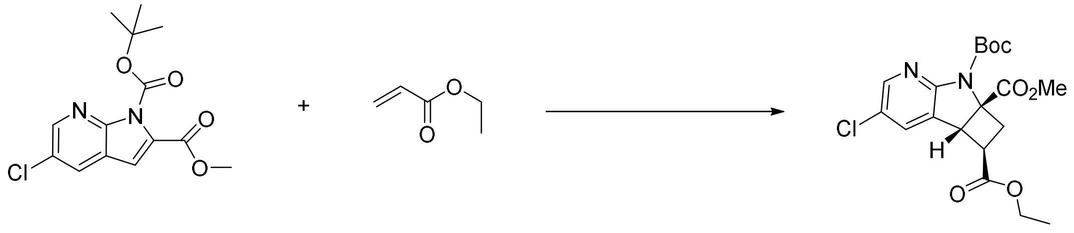

12k

Obtained as an oil (70% yield, crude: 5:1 dr) according to general procedure B.

^{1}H NMR (400 MHz, $CDCl_3$) δ 8.23 (d, *J* = 1.9 Hz, 1H), 7.38 (d, *J* = 2.3 Hz, 1H), 7.27 (s, 1H), 4.21 (q, *J* = 7.1 Hz, 2H), 4.12 (d, *J* = 5.9 Hz, 1H), 3.73 (s, 3H), 3.50 (dd, *J* = 13.6, 8.3 Hz, 1H), 3.11 (ddd, *J* = 9.9, 8.4, 6.1 Hz, 1H), 2.71 (dd, *J* = 13.6, 10.1 Hz, 1H), 1.46 (s, 9H), 1.29 (t, *J* = 7.1 Hz, 3H); ^{13}C NMR (126 MHz, $CDCl_3$) δ 171.6, 169.4, 156.0, 148.4, 145.9, 132.1, 125.6, 125.2, 82.2, 65.5, 60.9, 52.3, 44.7, 40.6, 32.6, 27.6, 13.7; LCMS *m/z* $[M+H]^+$ calcd for $C_{19}H_{24}ClN_2O_6$ 411.20, found 411.20.

^{1}H NMR (400 MHz) of CZ-Me-CHO in methylene chloride-d_2

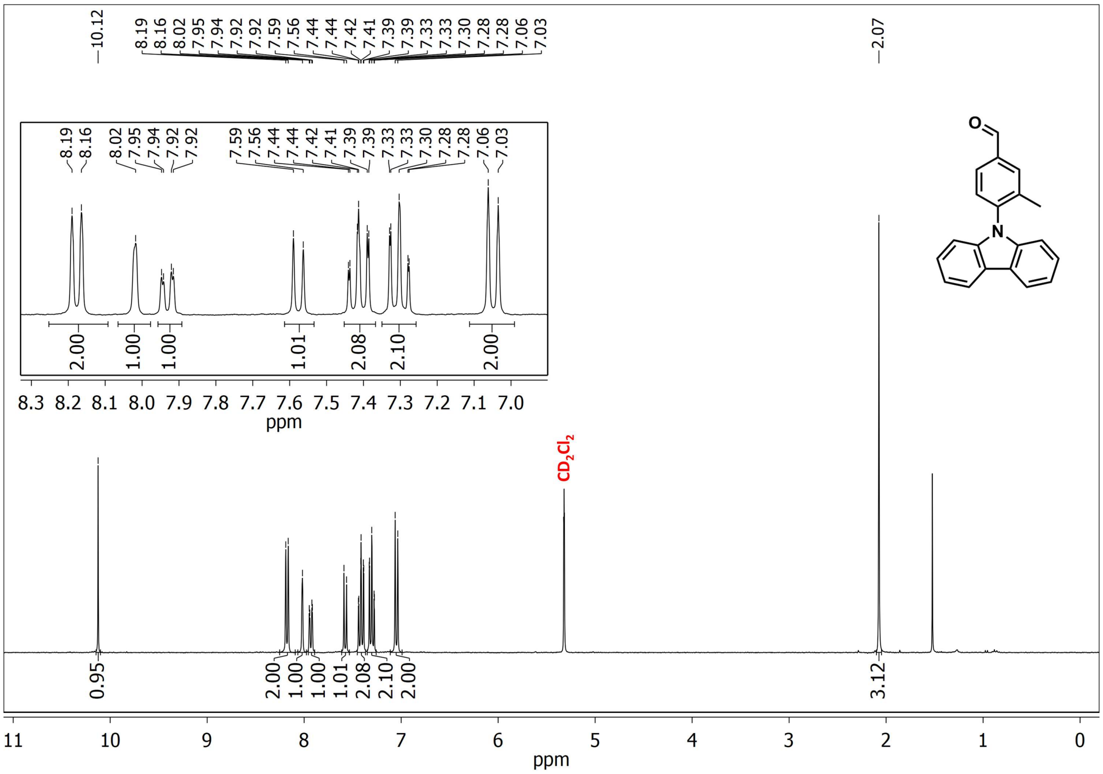

^{13}C{^{1}H} NMR (101 MHz) of CZ-Me-CHO in methylene chloride-d_2

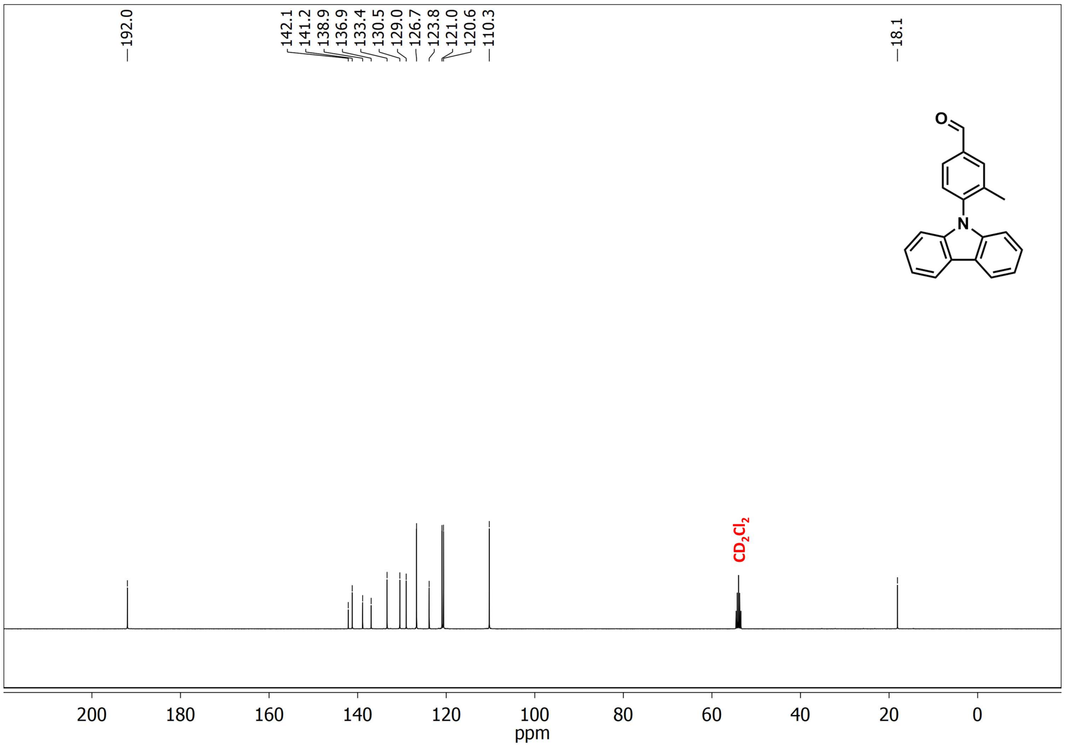

^{1}H NMR (400 MHz) of CZ-Me-IMAC in methylene chloride-d_2

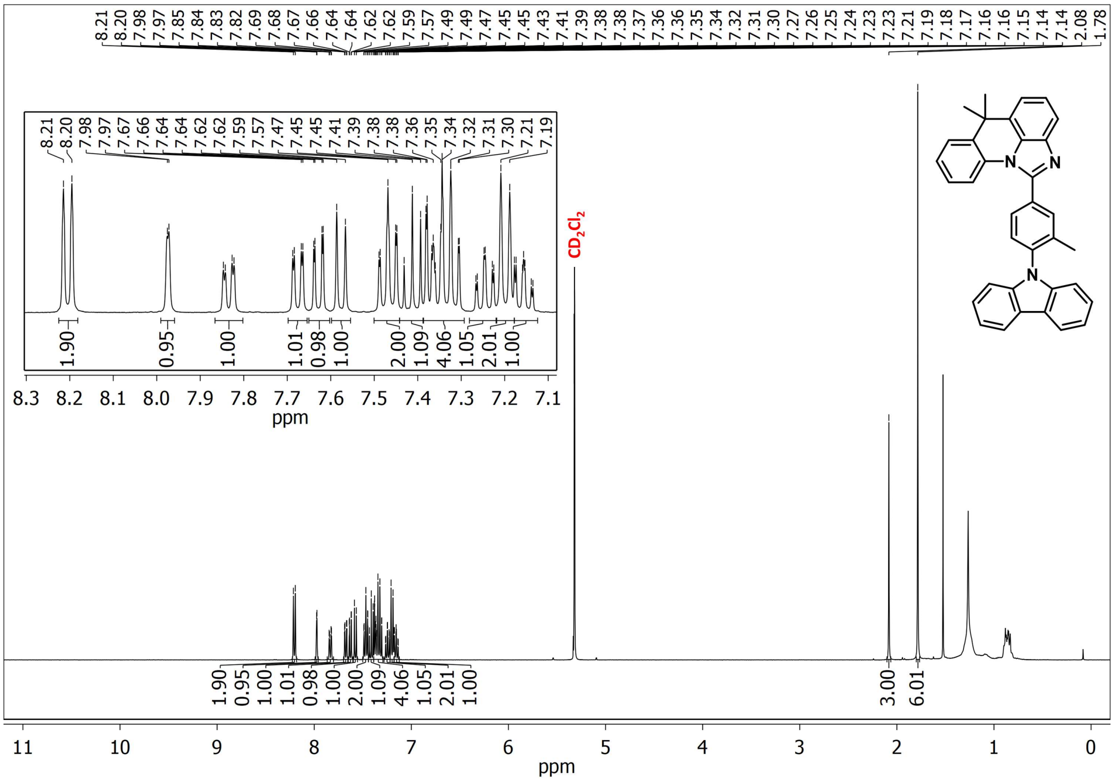

$^{13}C\{^{1}H\}$ NMR (101 MHz) of CZ-Me-IMAC in methylene chloride-d_2

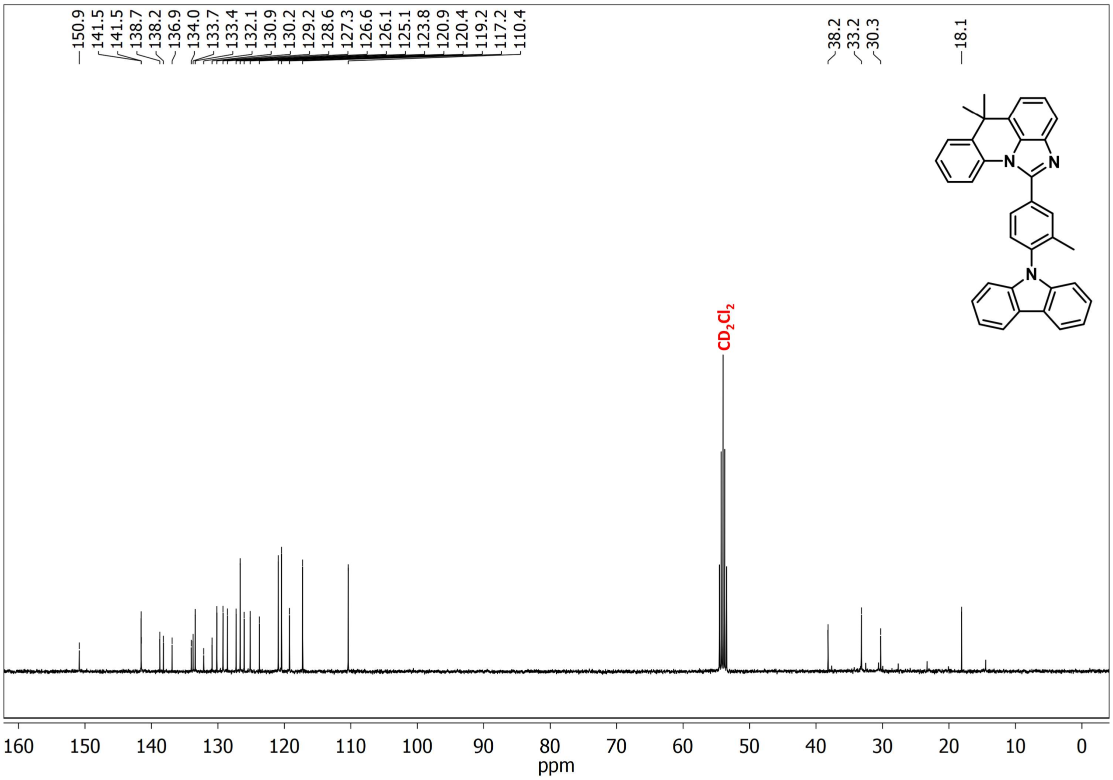

^{1}H-NMR of Spectrum Compound 4

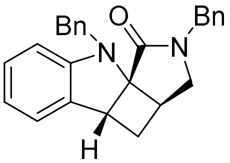

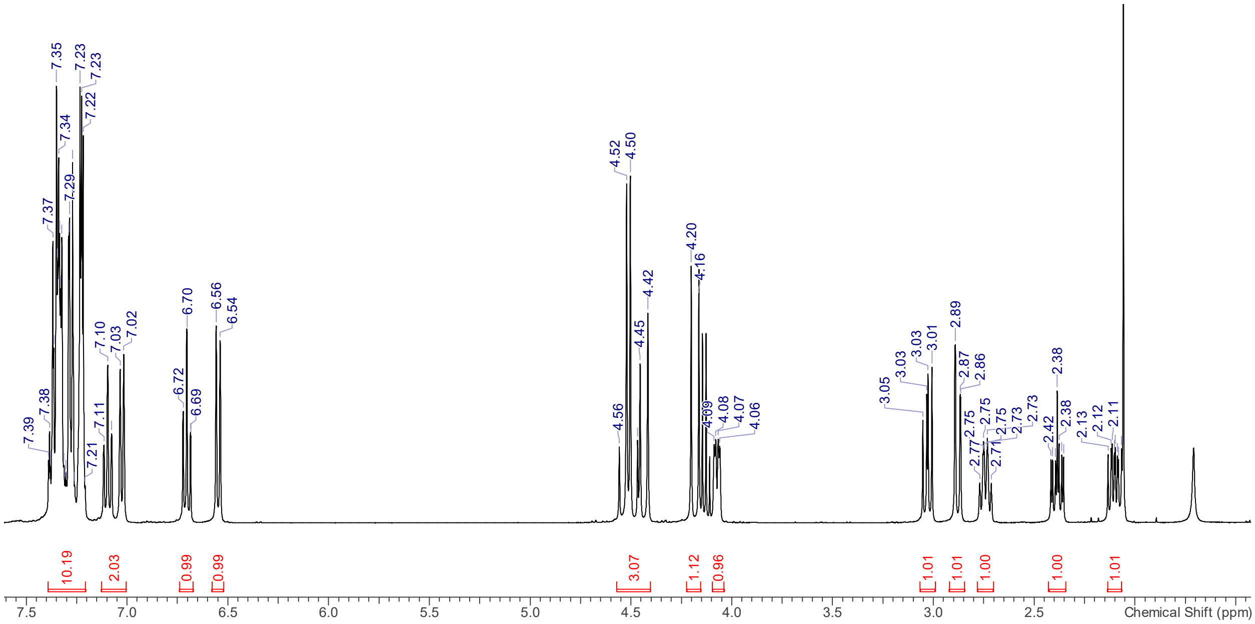

^{13}C-NMR of Spectrum Compound 4

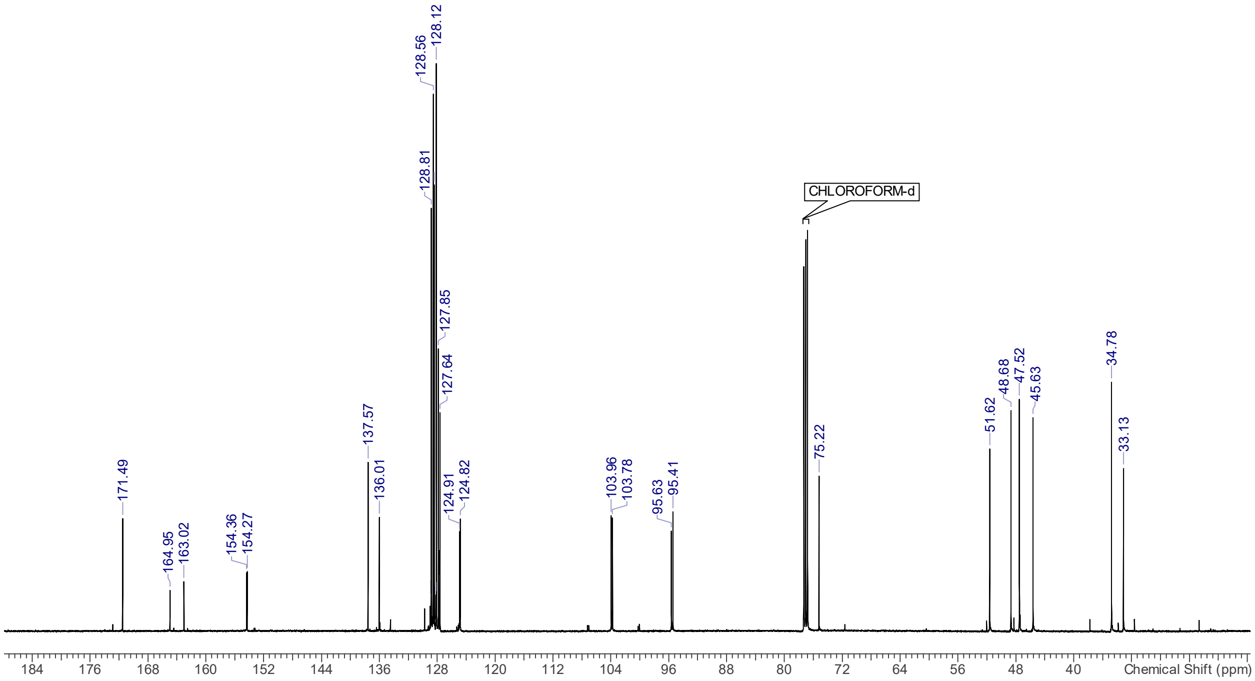

^{1}H-NMR of Spectrum Compound 6a

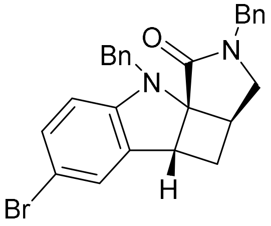

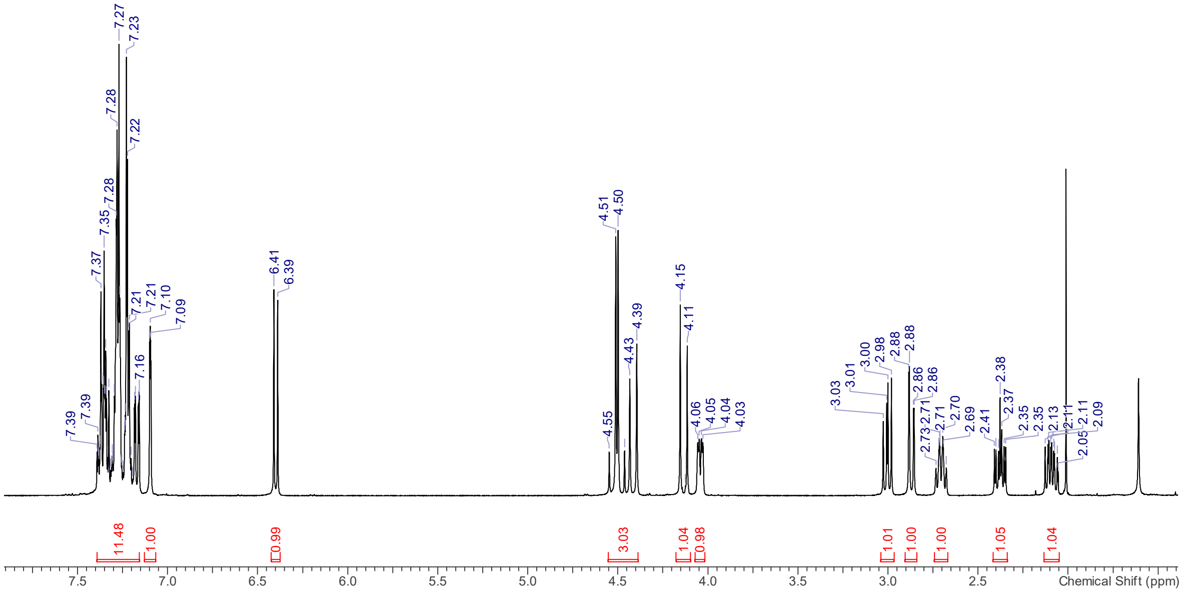

^{13}C-NMR of Spectrum Compound 6a

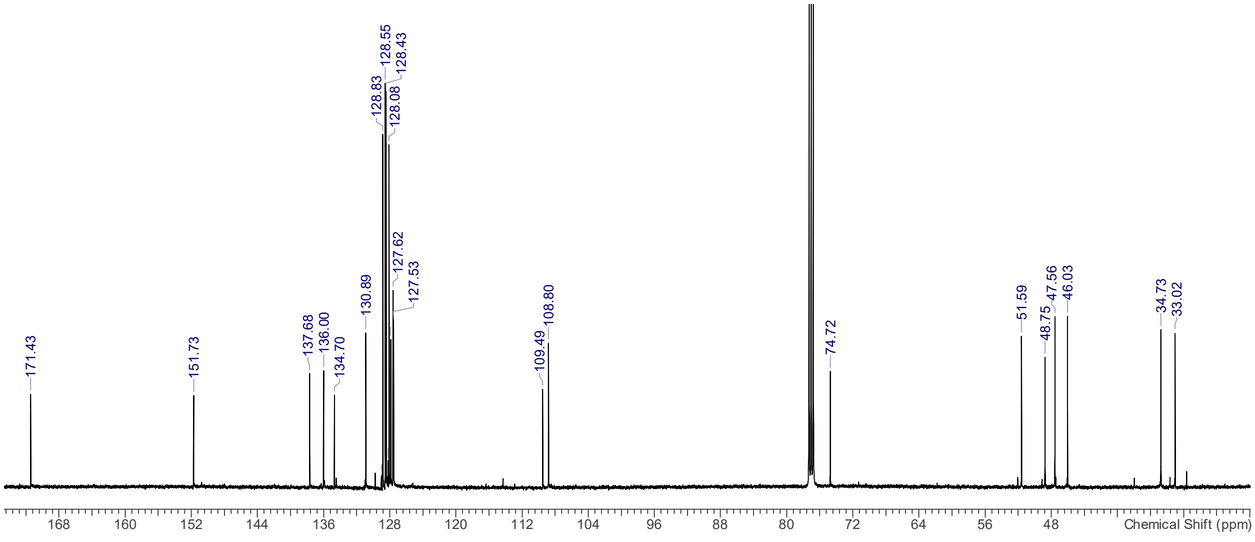

^{1}H-NMR of Spectrum Compound 6b

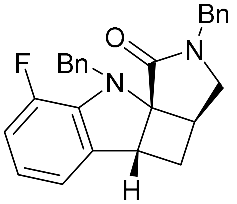

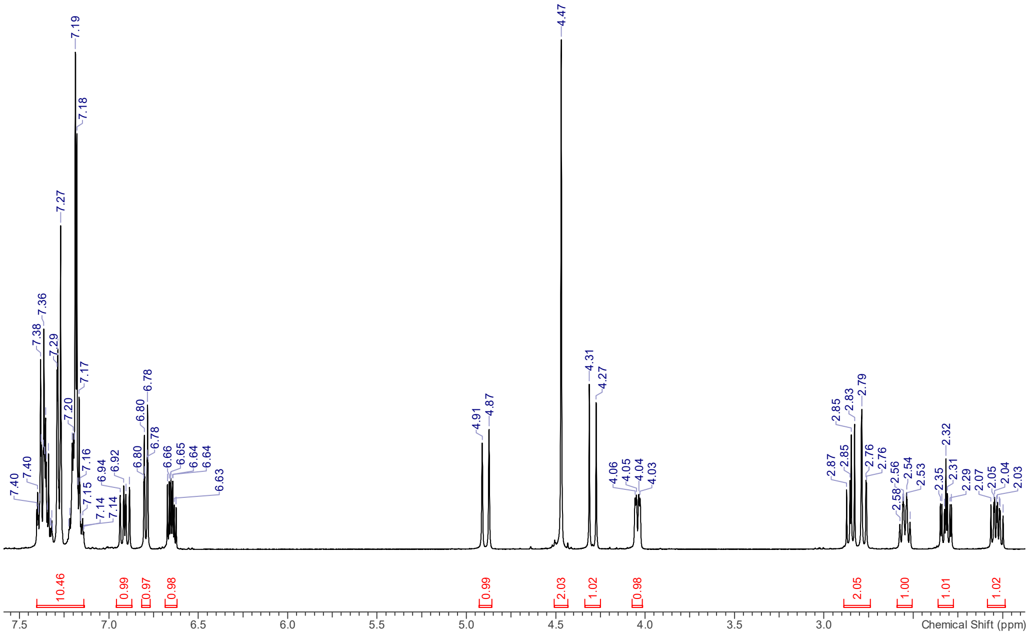

^{13}C-NMR of Spectrum Compound 6b

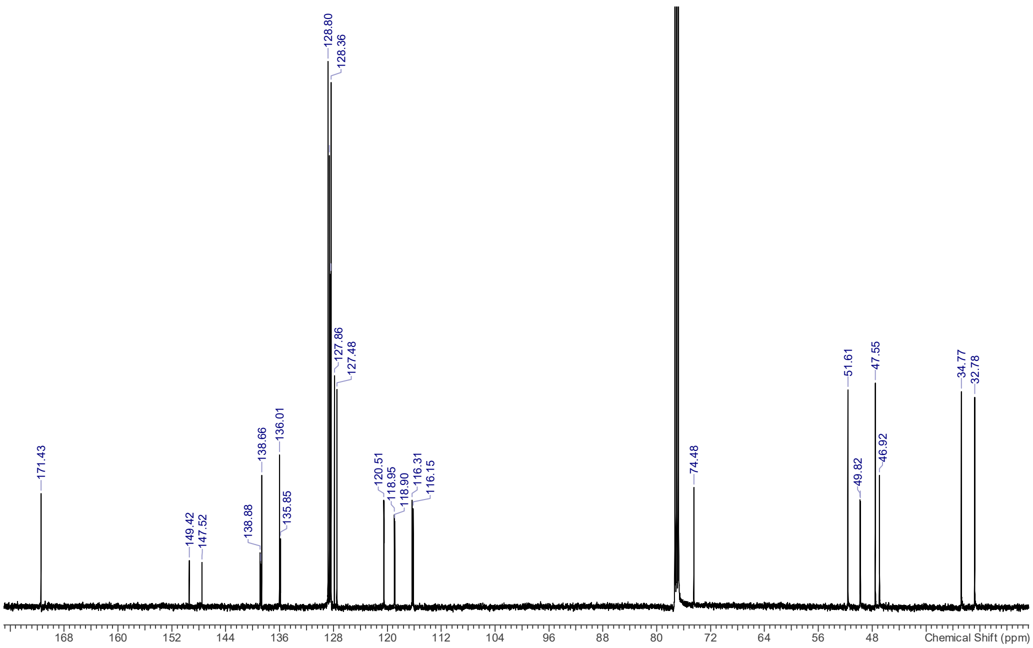

^{19}F NMR Spectrum of 6b

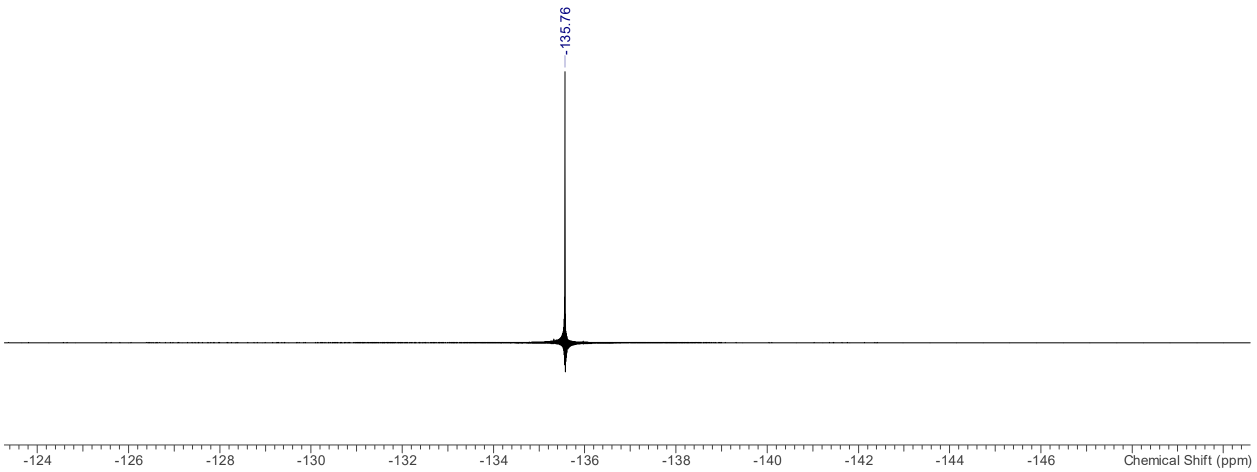

^{1}H-NMR of Spectrum Compound 6c

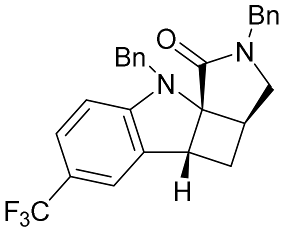

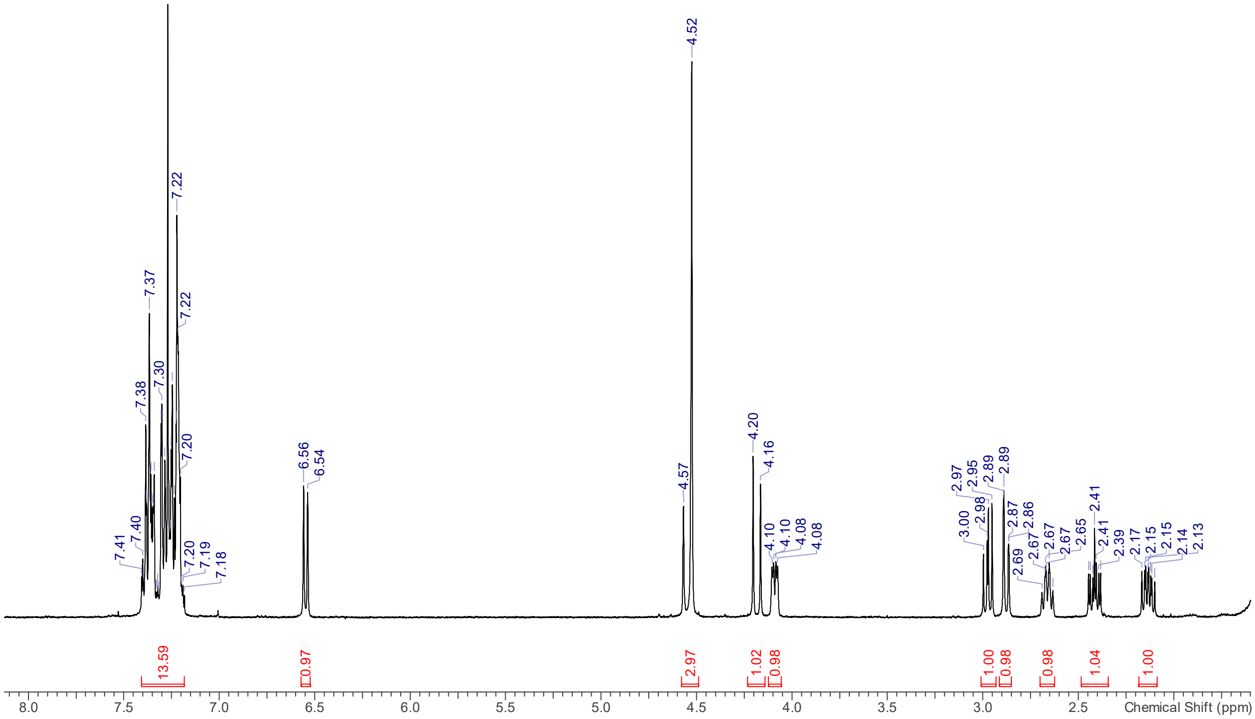

^{13}C-NMR of Spectrum Compound 6c

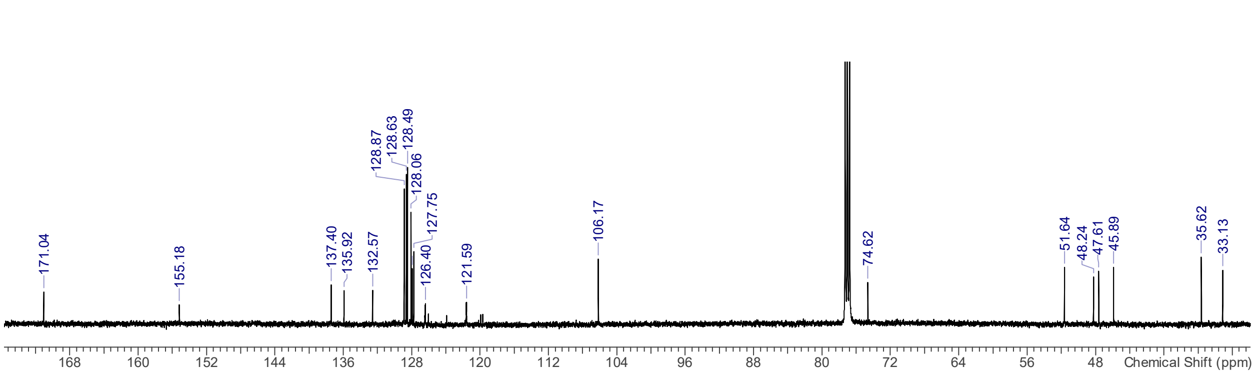

^{19}F-NMR of Spectrum Compound 6c

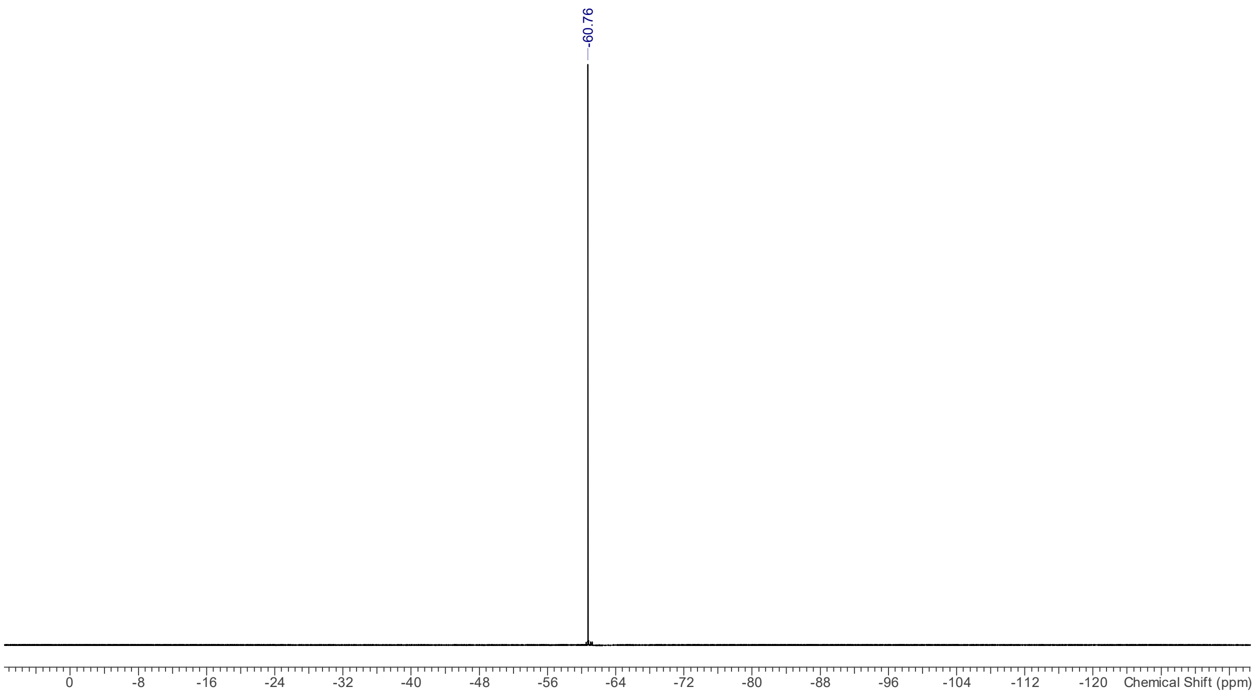

^{1}H-NMR of Spectrum Compound 6d

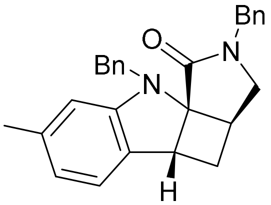

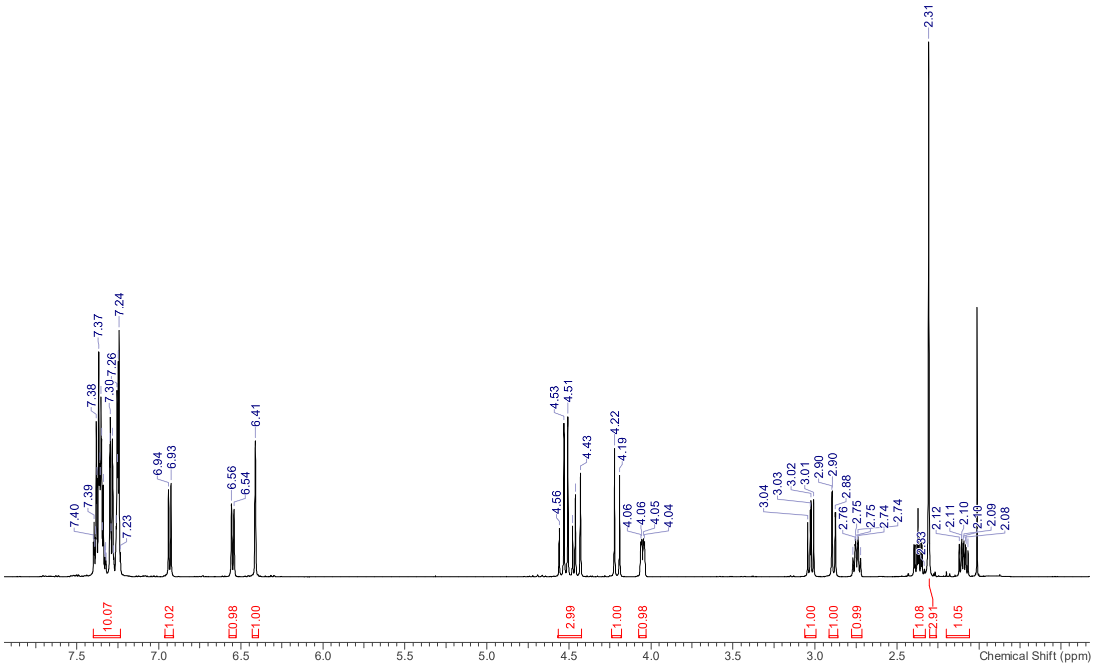

^{13}C-NMR of Spectrum Compound 6d

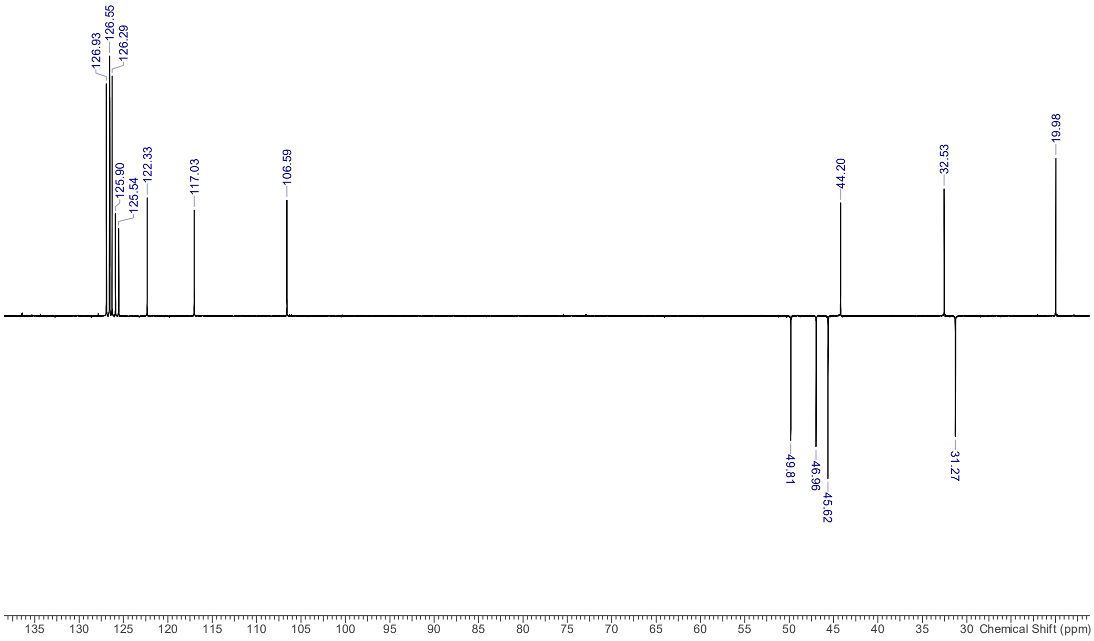

^{1}H-NMR of Spectrum Compound 6e

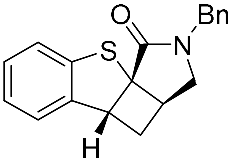

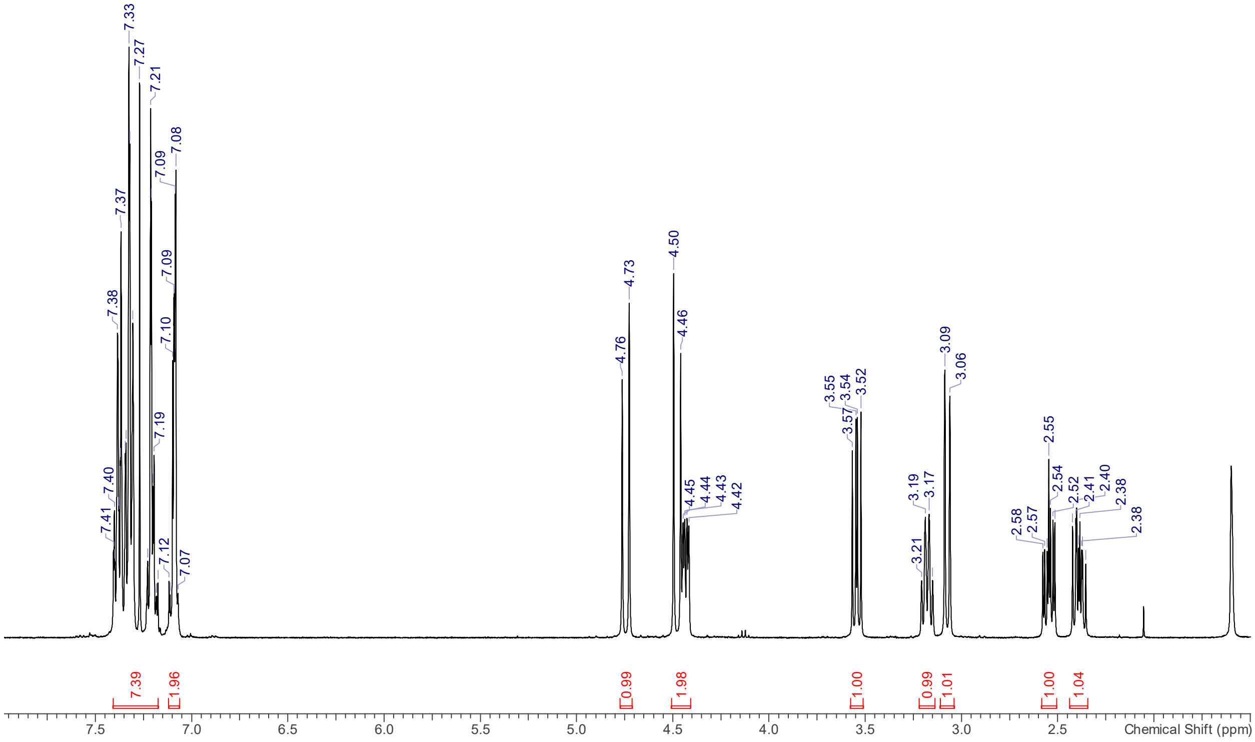

^{13}C-NMR of Spectrum Compound 6e

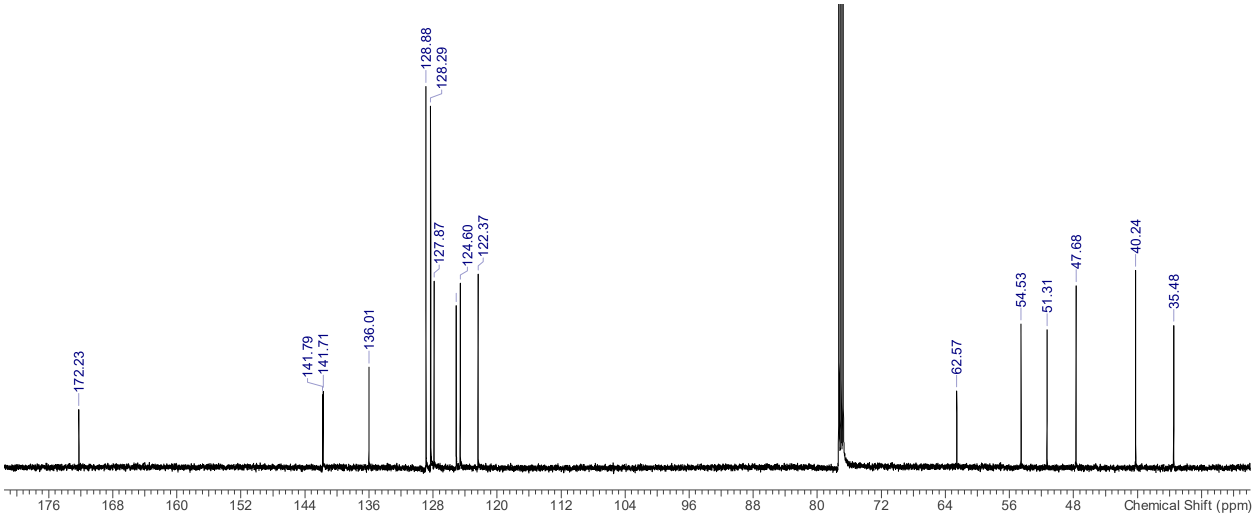

^{1}H-NMR of Spectrum Compound 6f

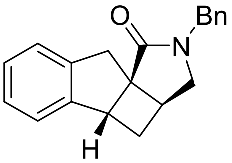

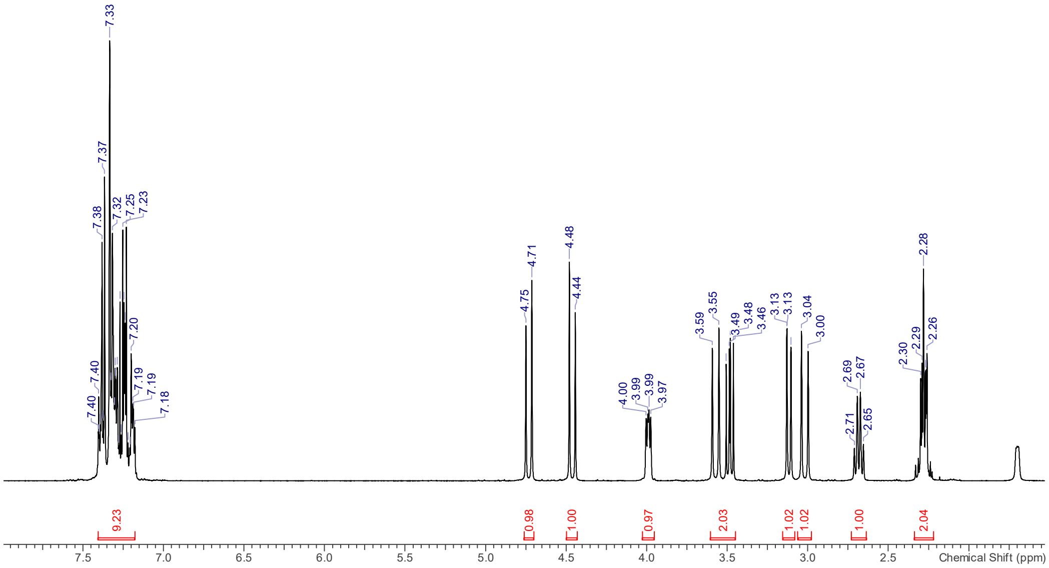

^{13}C-NMR of Spectrum Compound 6f

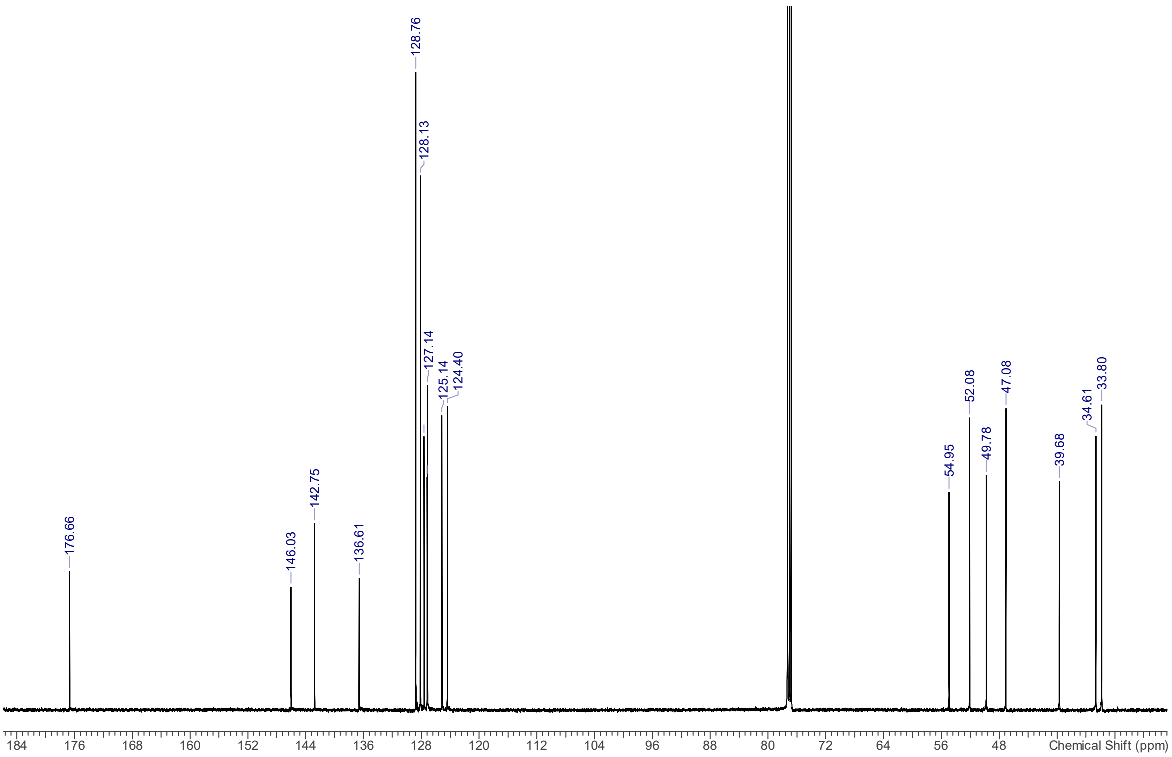

Compound 7

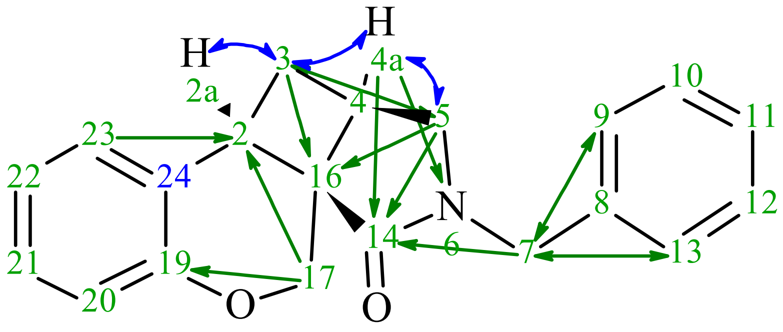

^{1}H-^{1}H COSY

^{1}H-^{13}C HMBC

^{1}H-^{15}N HMBC

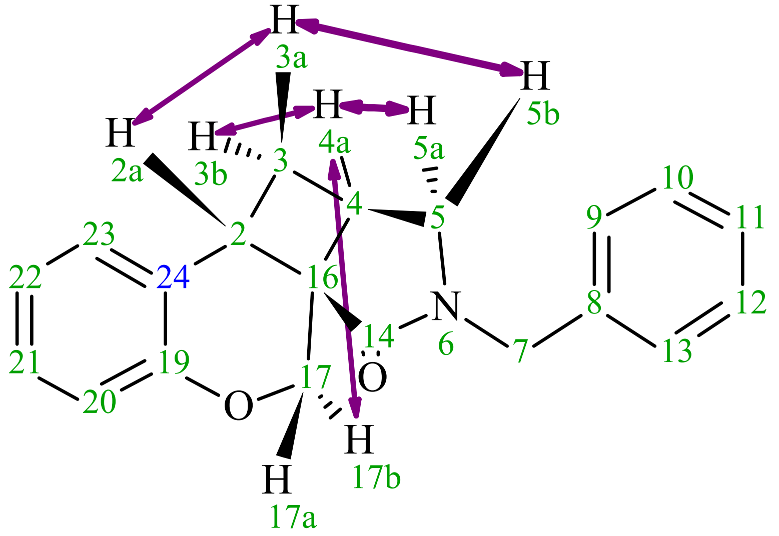

^{1}H-^{1}H NOESY

1D proton spectrum of 7 in $CDCl_3$ at 27 ℃

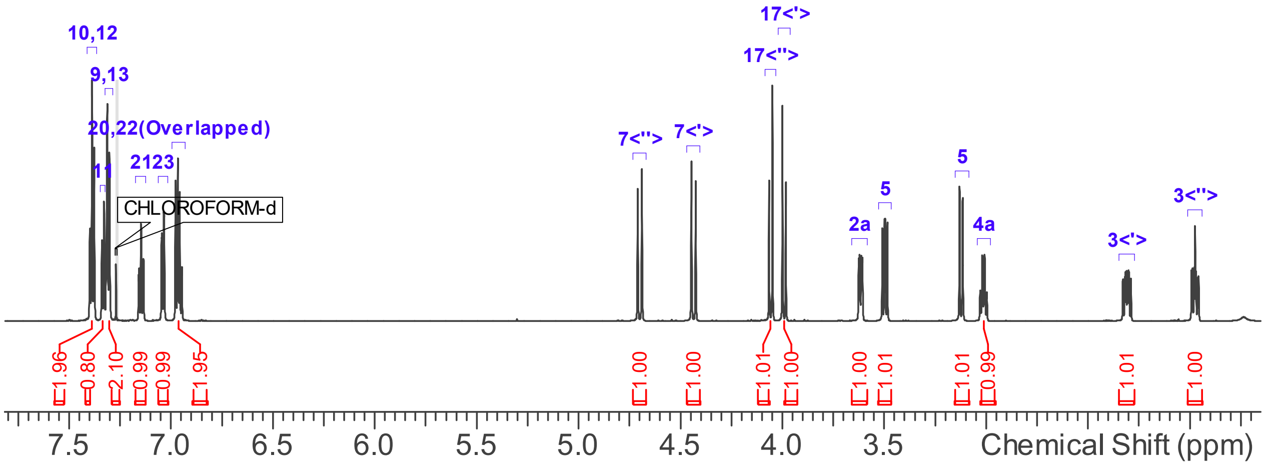

1D carbon spectrum of compound 7 in $CDCl_3$ at 27 ℃

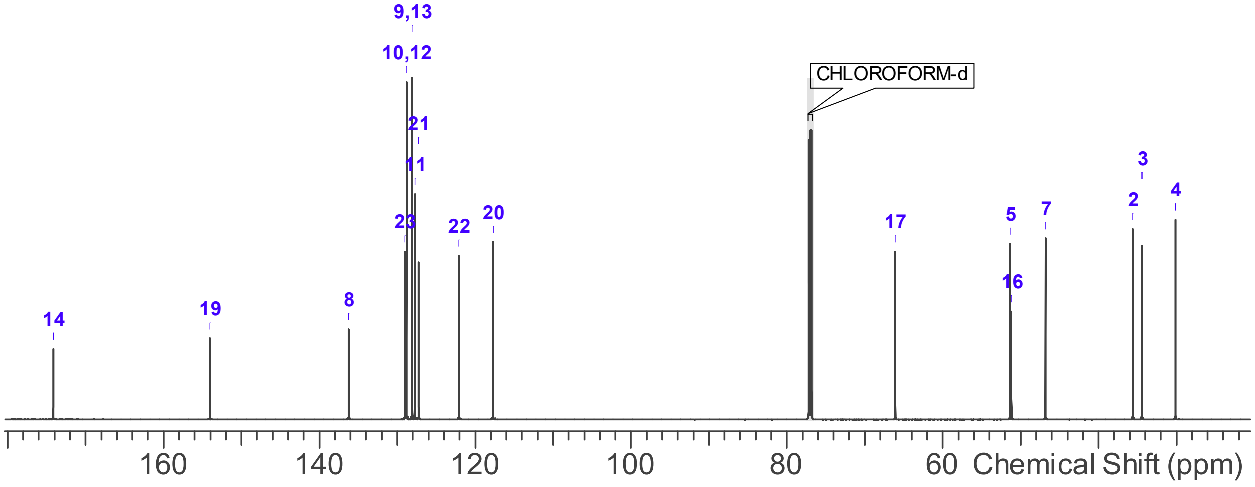

2D ^{13}C-HSQC carbon spectrum of compound 7 in $CDCl_3$ at 27 ℃

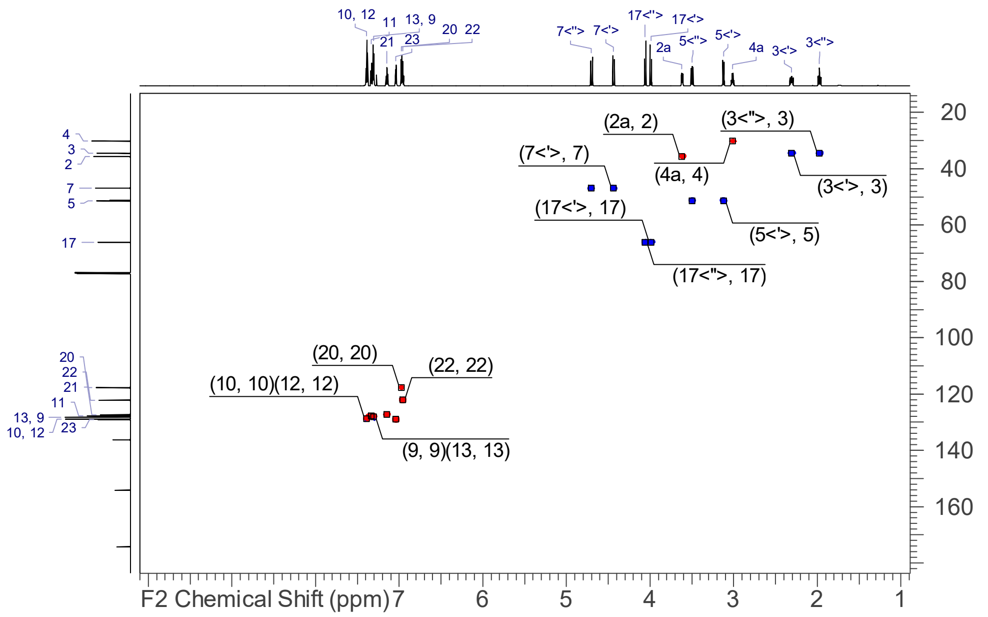

2D ^{1}H-^{1}H-NOESY carbon spectrum of compound 7 in $CDCl_3$ at 27 ℃

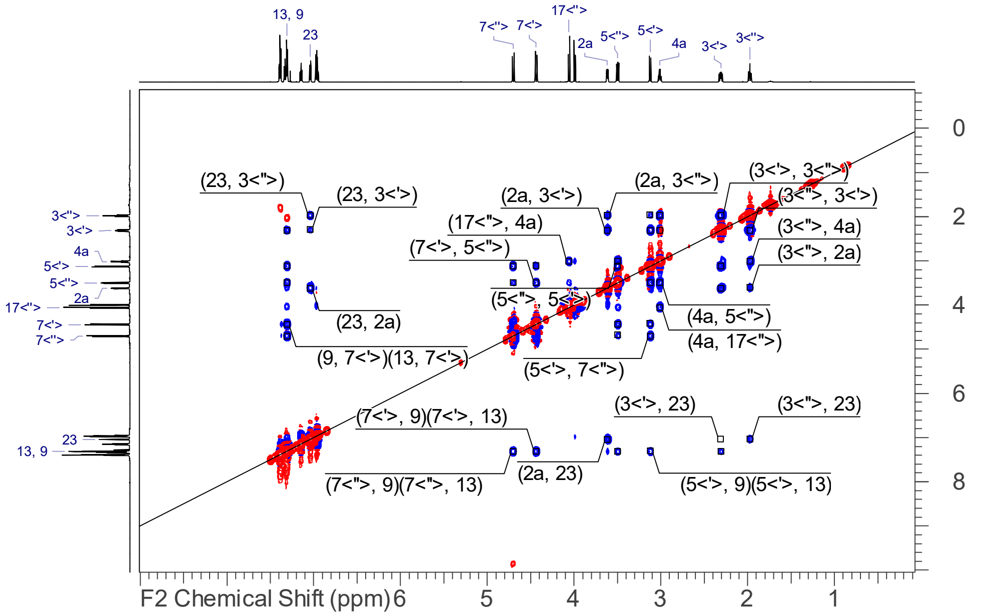

Chemical Shifts* and Coupling Constants of compound 7 in $CDCl_3$ at 27 °C

Atom#	XHn	H Shift	H Multiplicity	C Shift	X Shift
2a	CH	3.62	m (8.90, 3.39)	35.6	
3	CH2	2.31	ddd (12.19, 9.01, 6.78)	34.4	
3	CH2	1.98	ddd (12.29, 8.90, 3.39)	34.4	
4a	CH	3.01	m	30.1	
5	CH2	3.50	dd (10.60, 7.21)	51.3	
5	CH2	3.12	d (10.60)	51.3	
6	N				124.6
7	CH2	4.70	m (14.41)	46.8	
7	CH2	4.44	d (14.83)	46.8	
8	C			136.2	
9, 13	CH	7.31	d (7.21)	128.1	
10, 12	CH	7.39	m	128.8	
11	CH	7.33	d (7.21)	127.7	
14	C			174.1	
16	C			51.3	
16, 5	C, CH2			51.2	
17	CH2	4.06	m (11.45)	66.1	
17	CH2	3.99	d (11.45)	66.1	
19	C			154.0	
20	CH	6.97	m	117.7	
21	CH	7.15	m	127.3	
22	CH	6.96	m	122.1	
23	CH	7.04	dd (7.63, 1.27)	129.0	
24	C			127.7	

NOESY Table for compound 7 in $CDCl_3$ at 27℃.

F2 (ppm)	F1 (ppm)	Abs. Volume	Strength	Assignments
1.97	2.31	6.19	strong	3<">, 3<'>
1.97	3.01	0.55	medium	3<">, 4a
1.98	7.04	0.11	medium	3<">, 23
1.98	3.60	0.14	medium	3<">, 2a
2.30	7.31	0.05	weak	3<'>, 9; 3<'>, 13
2.31	1.97	5.82	strong	3<'>, 3<">
2.31	3.12	0.74	strong	3<'>, 5<'>
2.31	3.62	0.46	medium	3<'>, 2a
2.31	7.03	0.02	weak	3<'>, 23
3.01	1.97	0.84	strong	4a, 3<">
3.01	2.31	0.26	medium	4a, 3<'>
3.01	3.50	1.45	strong	4a, 5<">
3.01	4.06	0.21	medium	4a, 17<">
3.12	2.31	0.83	strong	5<'>, 3<'>
3.12	3.50	5.66	strong	5<'>, 5<">
3.12	4.70	0.26	medium	5<'>, 7<">
3.12	7.31	0.12	medium	5<'>, 9; 5<'>, 13
3.12	4.42	0.14	medium	5<'>, 7<'>
3.12	1.96	0.07	medium	5<'>, 3<">
3.50	3.01	0.78	strong	5<">, 4a
3.50	3.12	4.74	strong	5<">, 5<'>
3.50	4.44	0.32	medium	5<">, 7<'>
3.50	4.68	0.09	medium	5<">, 7<">
3.50	7.31	0.06	weak	5<">, 9; 5<">, 13
3.62	1.97	0.16	medium	2a, 3<">
3.62	2.31	1.02	strong	2a, 3<'>
3.62	7.04	0.63	strong	2a, 23
4.06	3.01	0.12	medium	17<">, 4a
4.44	3.10	0.17	medium	7<'>, 5<'>
4.44	3.50	0.24	medium	7<'>, 5<">
4.44	7.31	0.24	medium	7<'>, 9; 7<'>, 13
4.70	3.49	0.08	medium	7<">, 5<">
4.70	3.12	0.27	medium	7<">, 5<'>
4.70	7.31	0.24	medium	7<">, 9; 7<">, 13
7.04	2.29	0.06	weak	23, 3<'>
7.04	1.97	0.11	medium	23, 3<">
7.04	3.62	0.66	strong	23, 2a
7.31	2.31	0.06	medium	9, 3<'>; 13, 3<'>
7.31	3.12	0.11	medium	9, 5<'>; 13, 5<'>
7.31	3.50	0.11	medium	9, 5<">; 13, 5<">
7.31	4.44	0.61	medium	9, 7<'>; 13, 7<'>
7.31	4.70	0.52	medium	9, 7<">; 13, 7<">

¹H-NMR of Spectrum Compound 9a

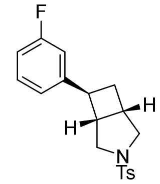

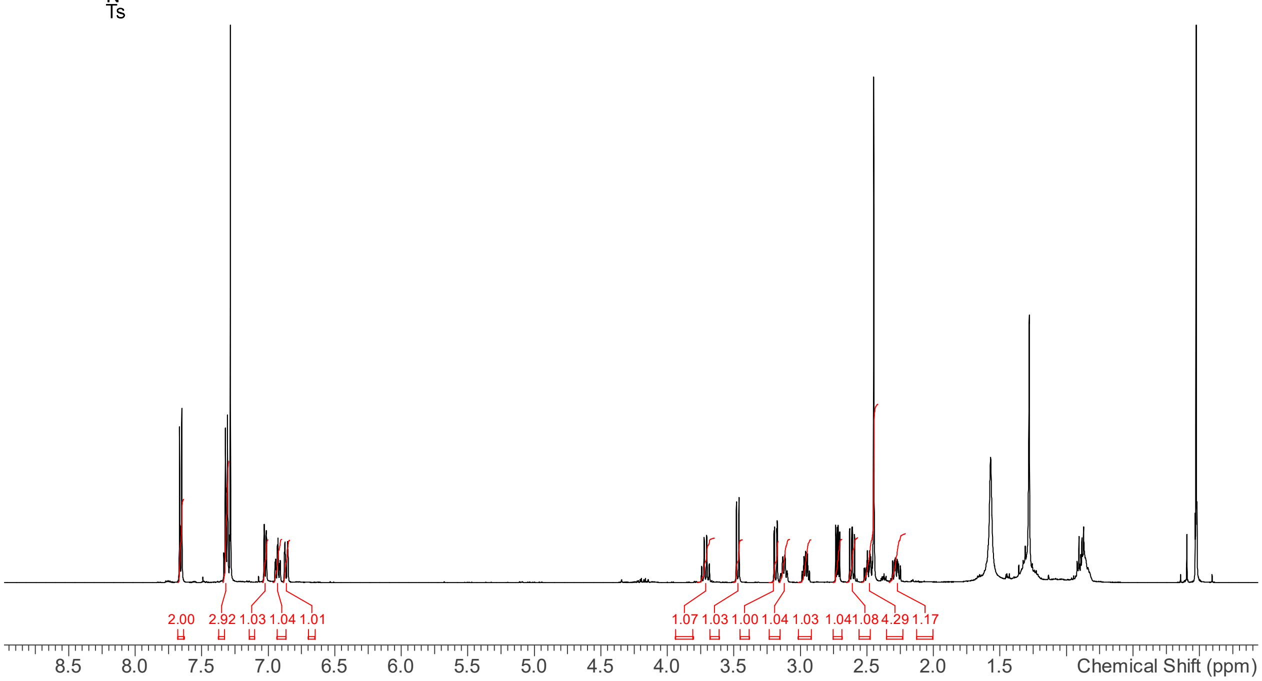

¹³C-NMR of Spectrum Compound 9a

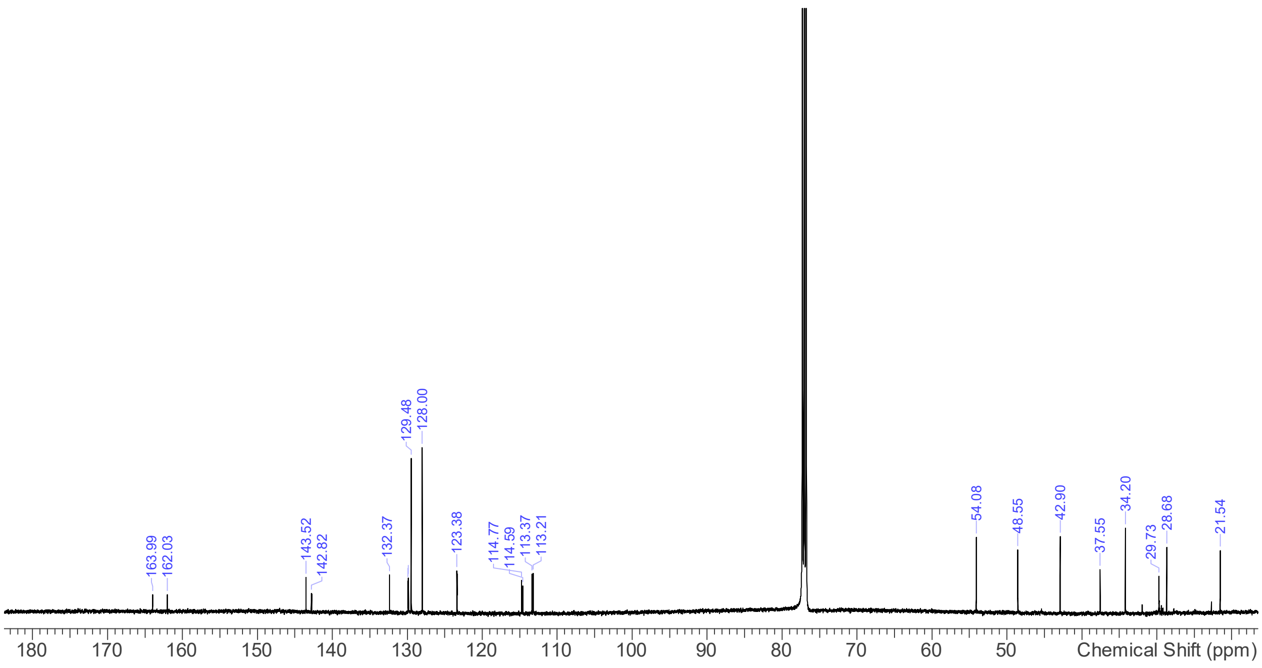

^{1}H-NMR of Spectrum Compound 9b

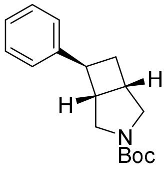

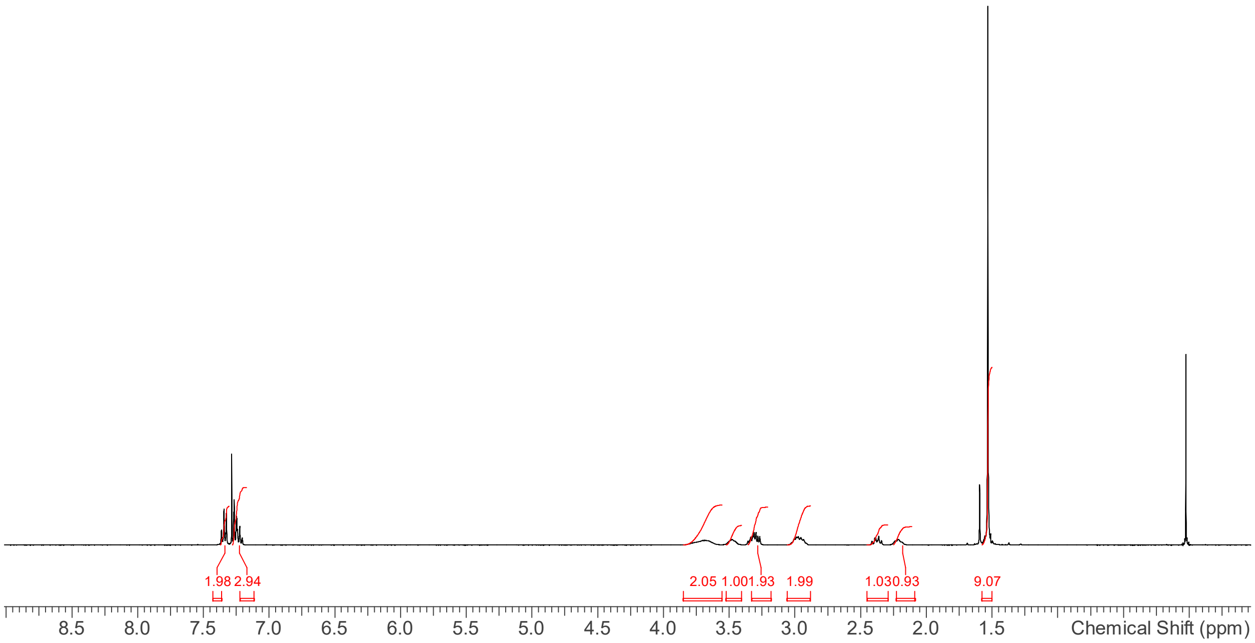

^{13}C-NMR of Spectrum Compound 9b

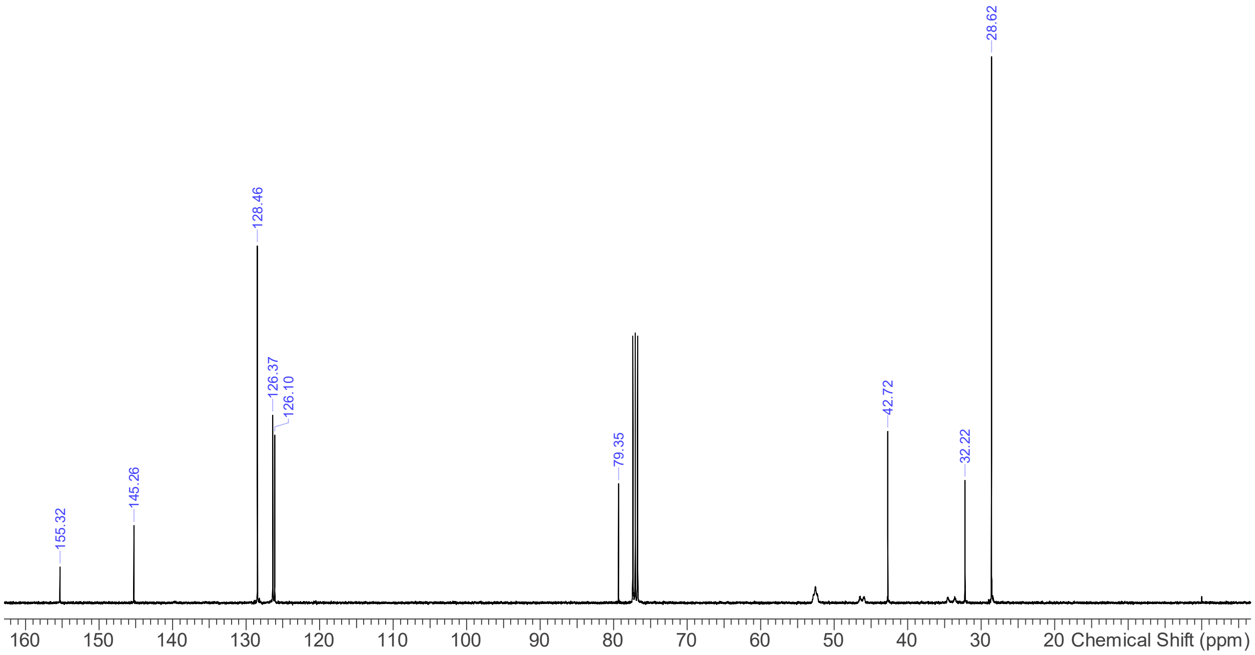

^{1}H-NMR of Spectrum Compound 9c

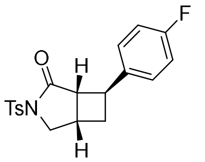

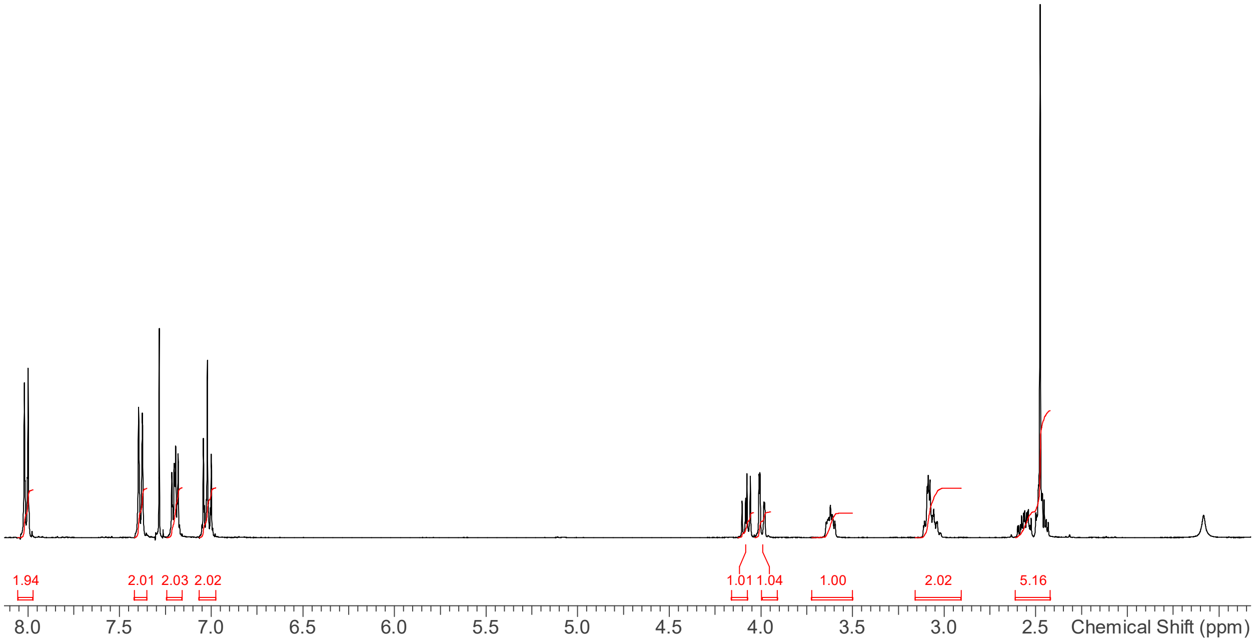

^{13}C-NMR of Spectrum Compound 9c

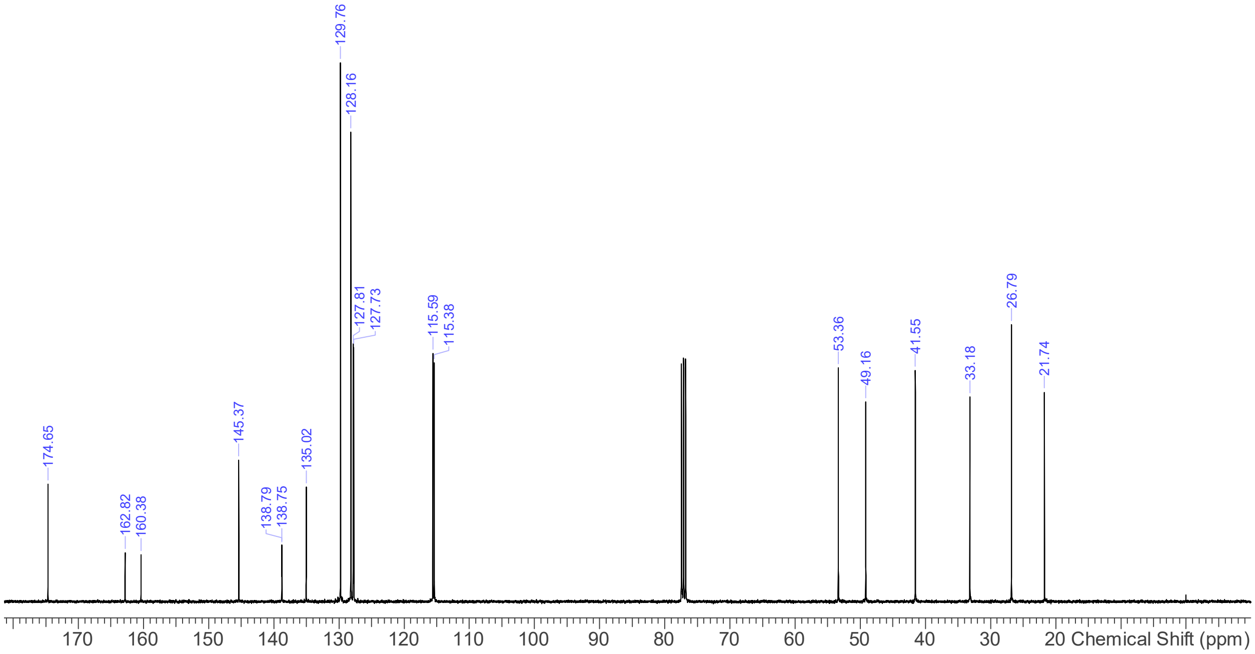

^{1}H-NMR Spectrum of Compound 12a

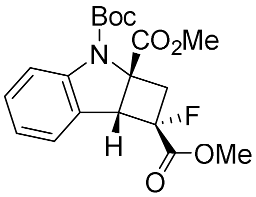

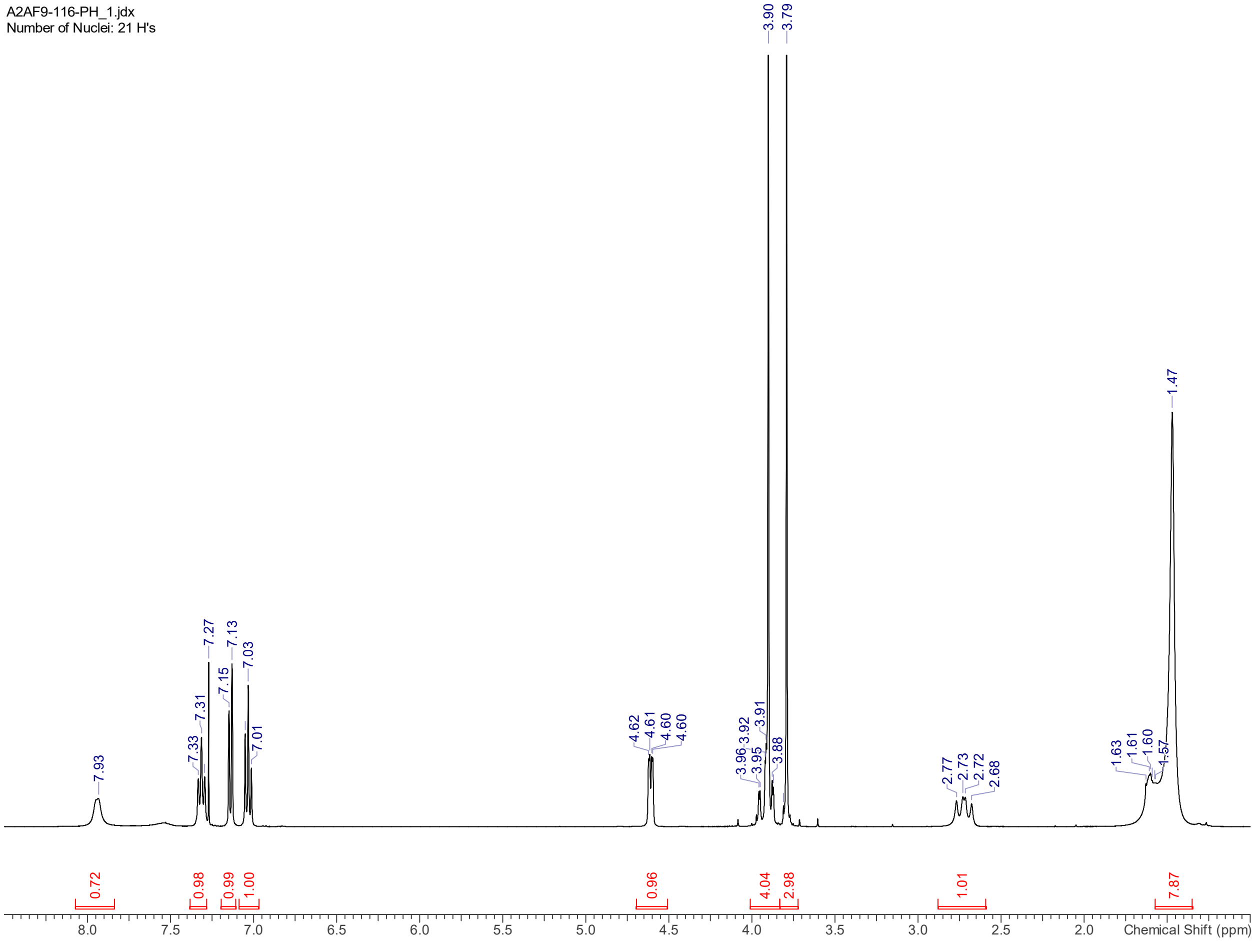

^{13}C-NMR Spectrum of Compound 12a

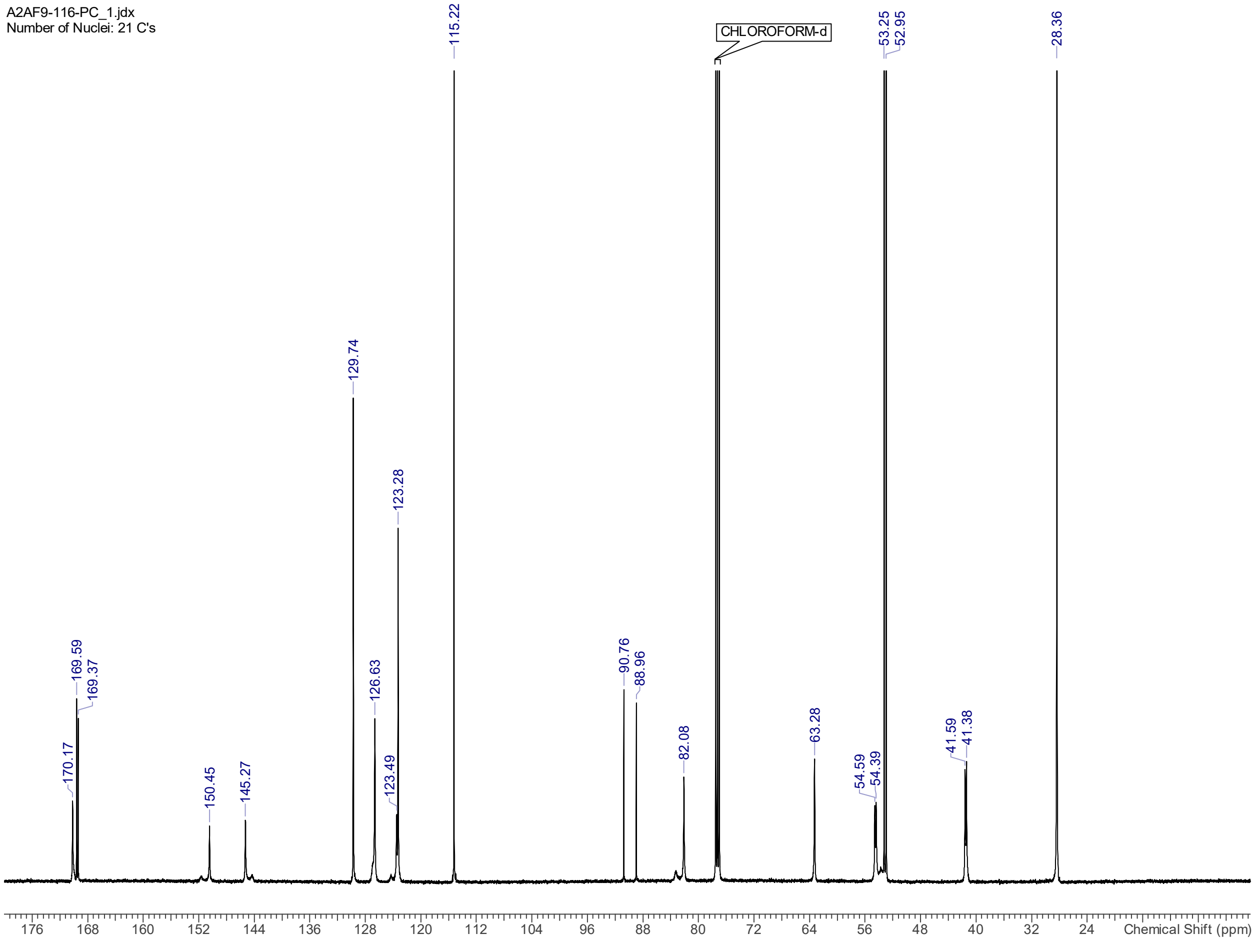

^{19}F-NMR Spectrum of Compound 12a

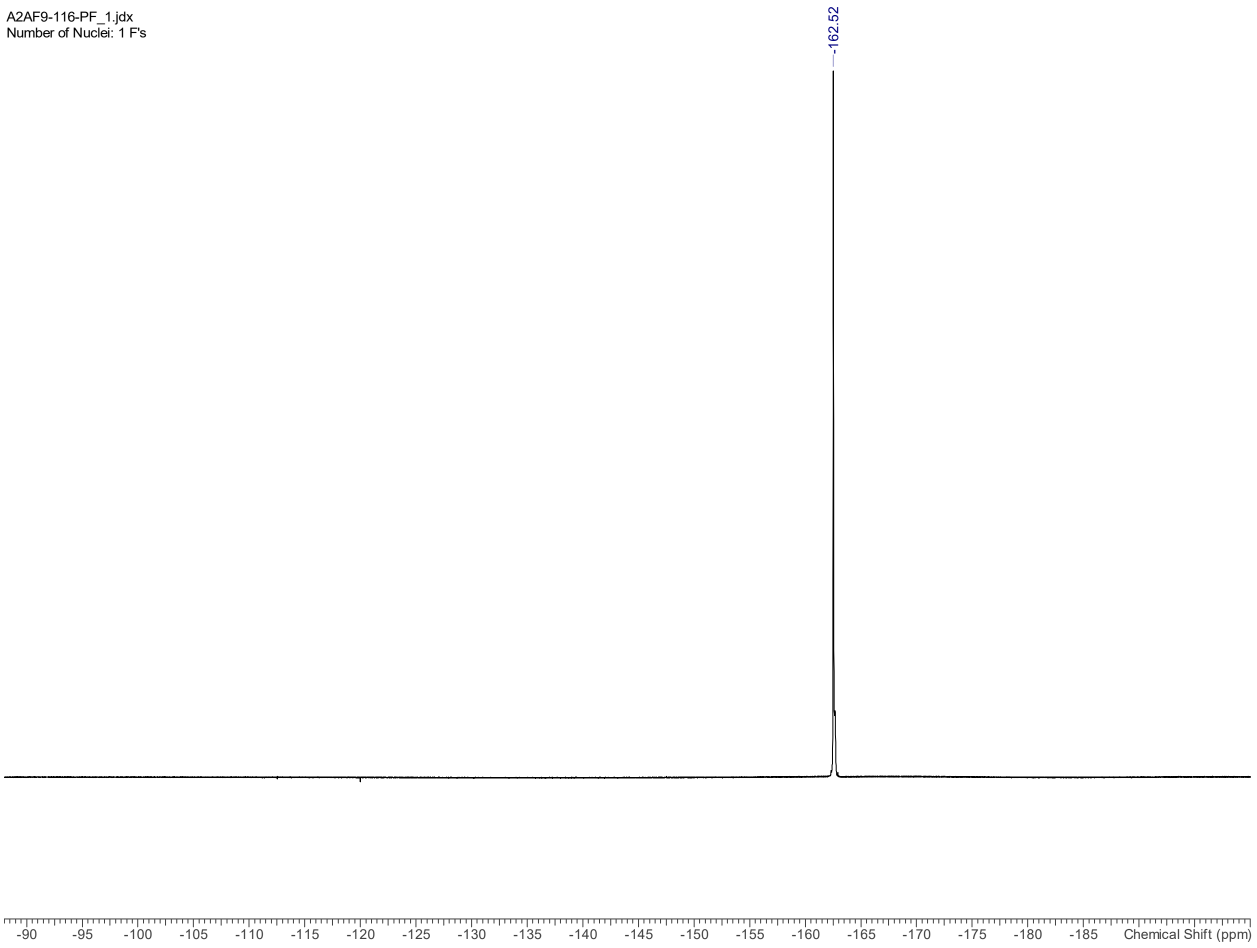

^{1}H-NMR spectrum of 12b

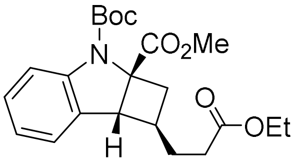

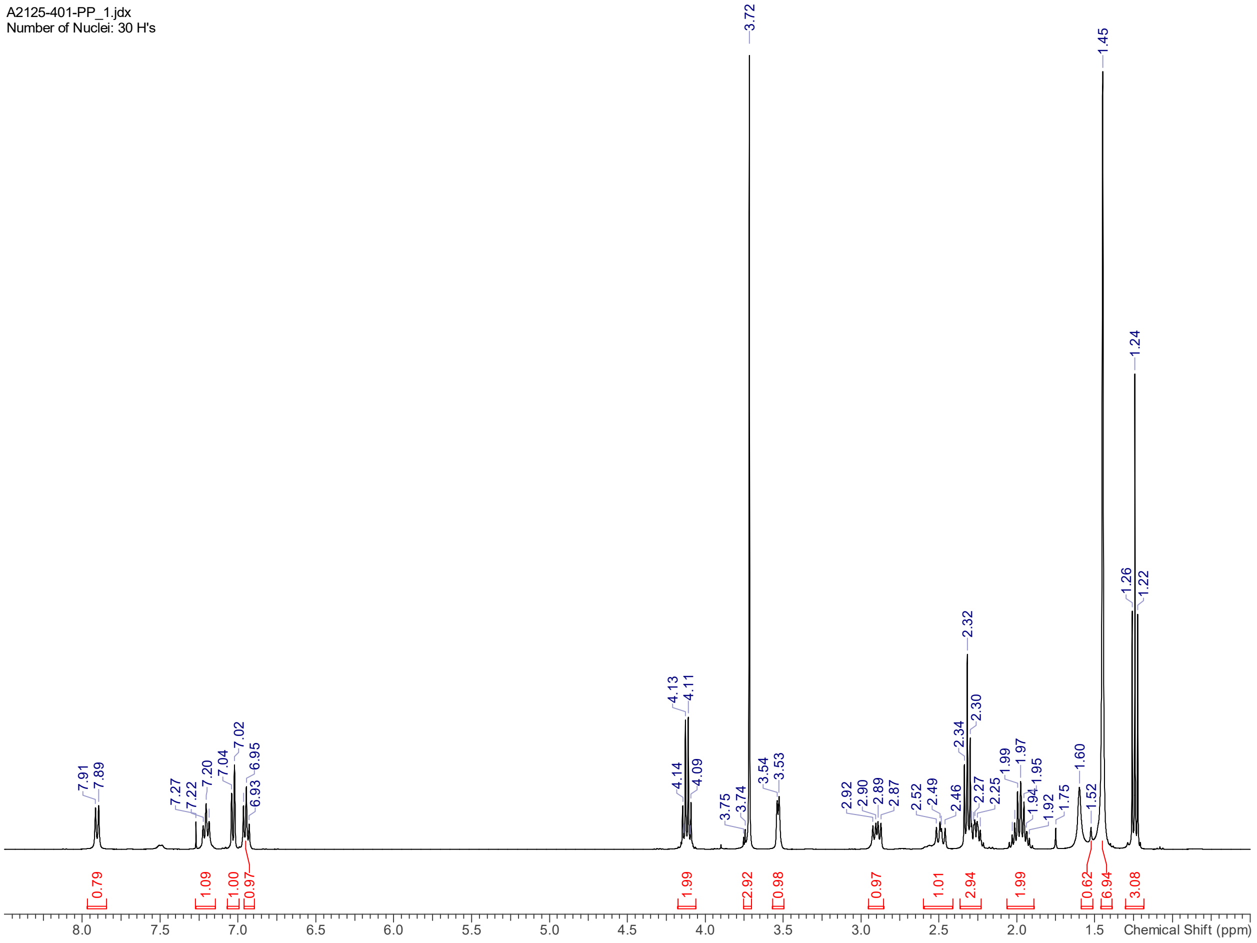

^{13}C-NMR spectrum of 12b

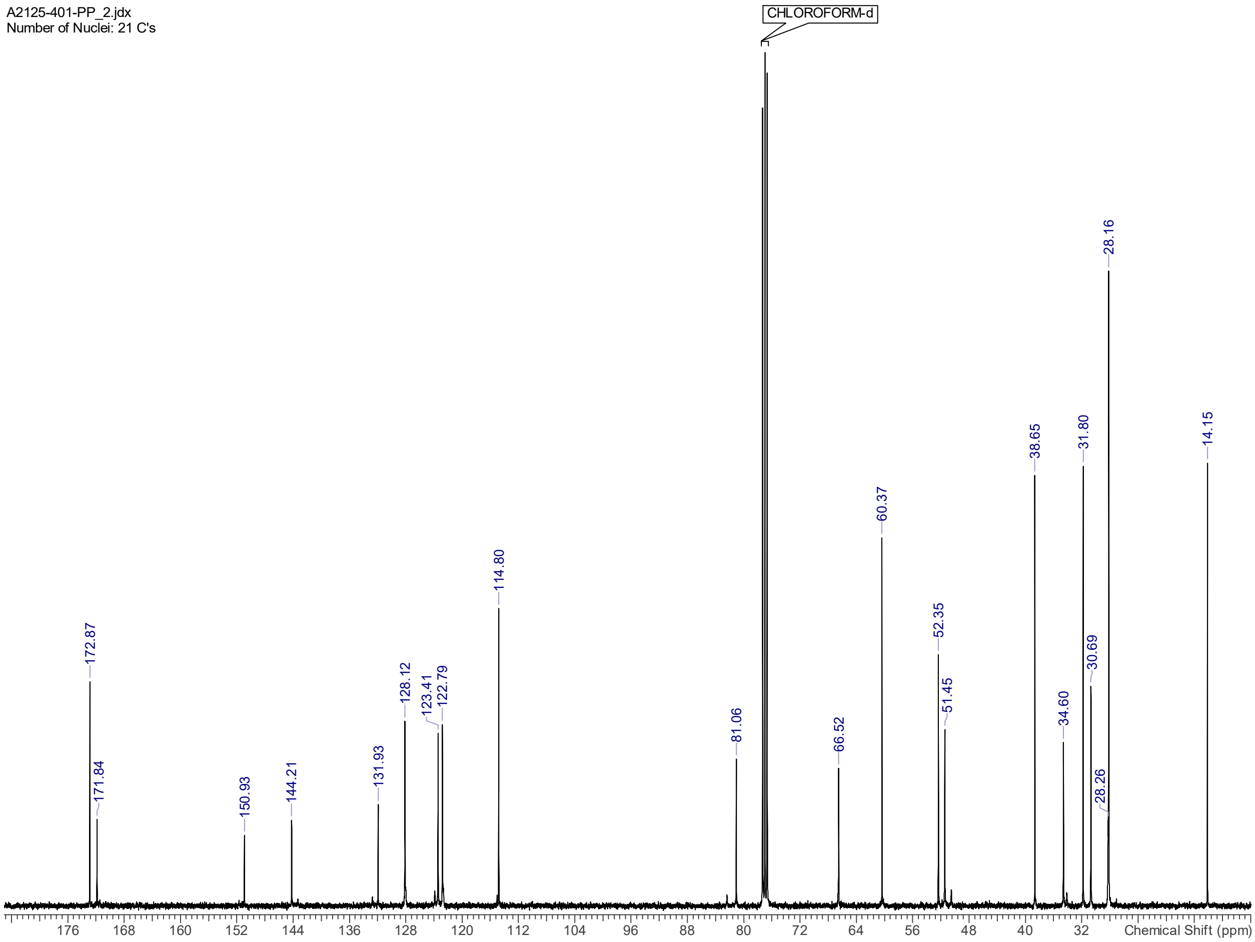

2D Elucidation of Compound 12c

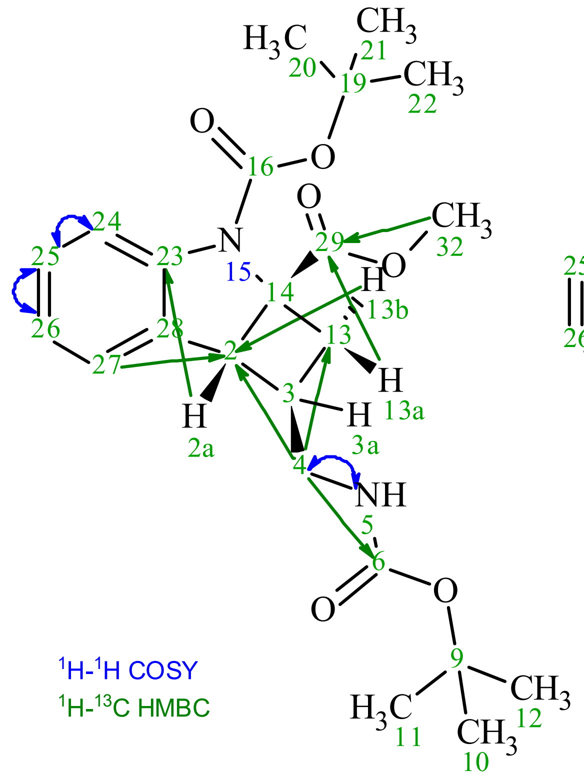

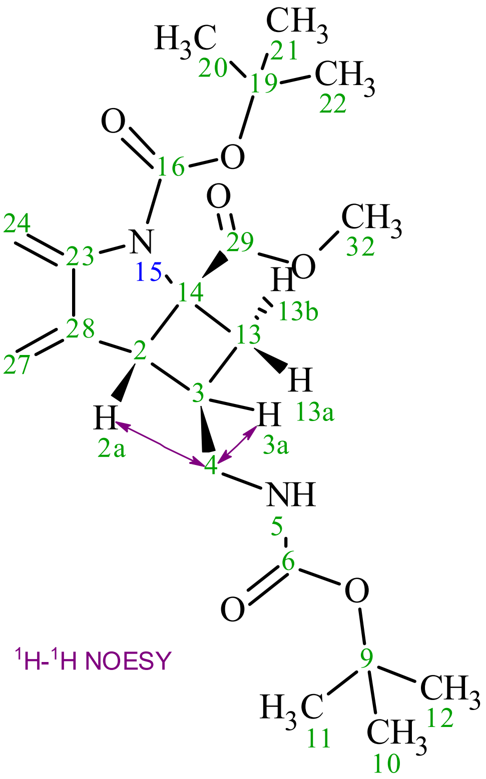

1D proton spectrum of 12c in DMSO-d6 at 27 ℃

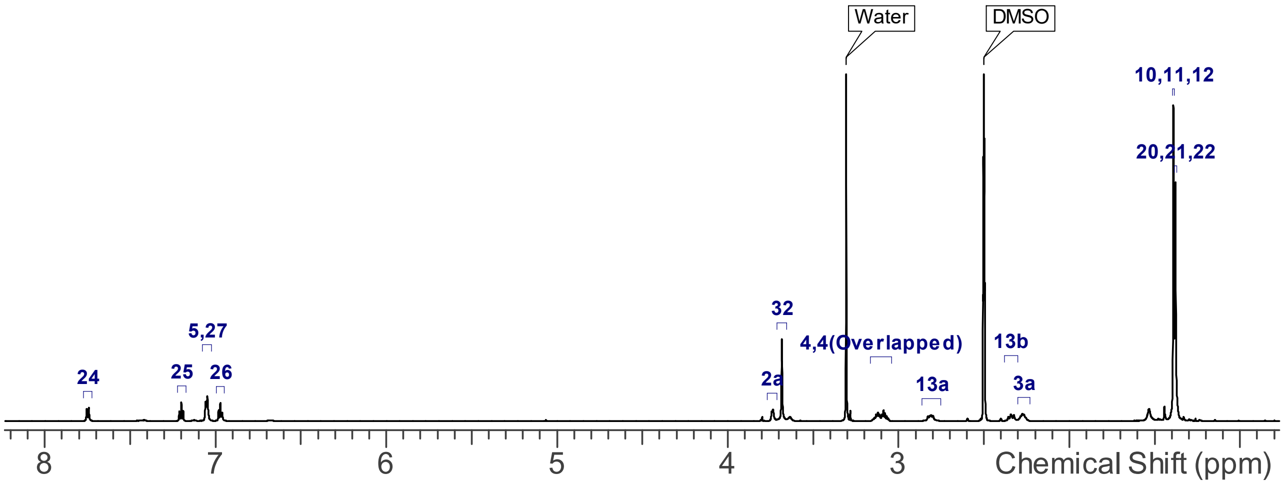

1D carbon spectrum of 12c in DMSO-d6 at 27 ℃

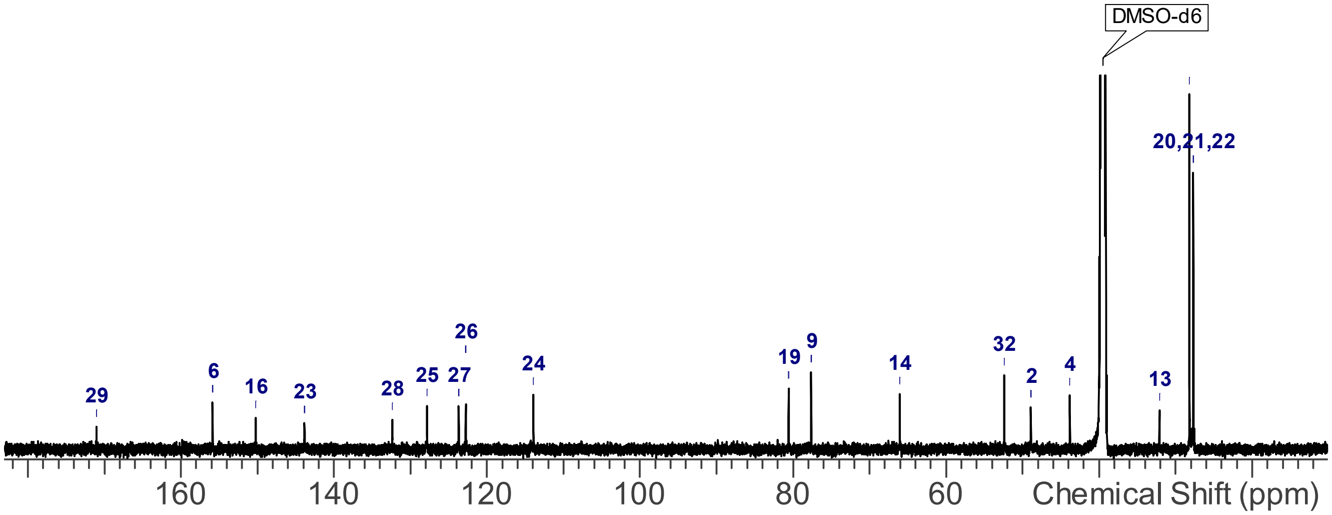

^{1}H-^{13}C DEPT-HSQC spectrum of 12c in DMSO-d6 at 27 ℃

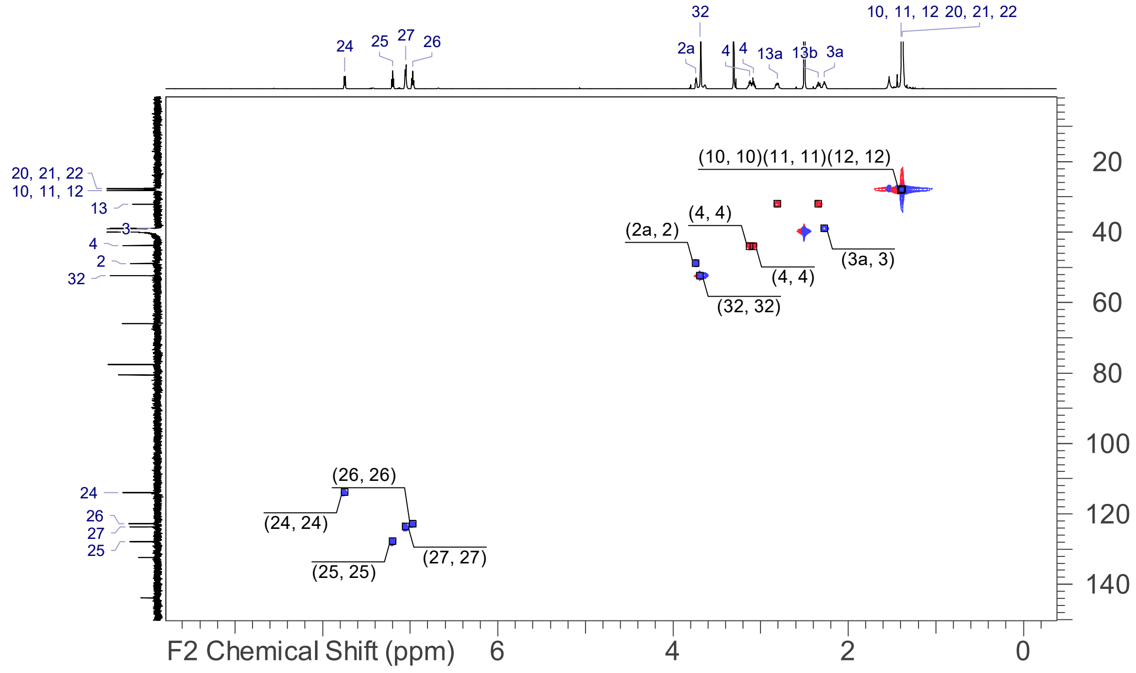

^{1}H-^{1}H NOESY spectrum of 12c in DMSO-d6 at 27 ℃

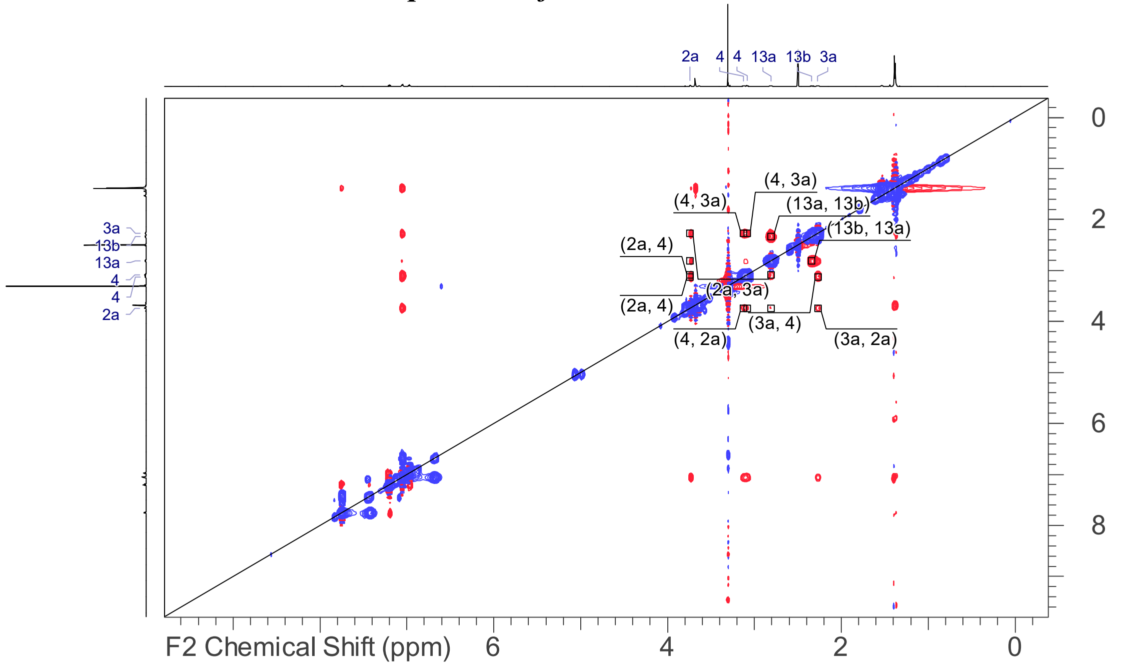

Chemical Shifts and Coupling Constants of 12c in DMSO-d6 at 27 °C*

Atom#	XHn	H Shift	H Multiplicity	C Shift	X Shift
2a	CH	3.74	br d (4.24)	48.925	
3a	CH	2.27	m	38.859	
4	CH2	3.08	m	43.829	
4	CH2	3.13	m	43.829	
5	NH	7.05	br d (7.21)		
5	NH				82.238
6	C			155.865	
9	C			77.632	
10, 11, 12	CH3	1.40	s	28.21	
13a	CH2	2.81	br dd (12.93, 7.42)	32.1	
13b	CH2	2.34	m	32.1	
14	C			66.1	
16	C			150.2	
19	C			80.6	
20, 21, 22	CH3	1.385	s	27.69	
23	C			143.853	
24	CH	7.75	d (8.05)	113.926	
25	CH	7.199	t (7.84, 7.84)	127.83	
26	CH	6.972	t (7.42, 7.42)	122.777	
27	CH	7.051	br d (7.21)	123.695	
28	C			132.387	
29	C			171.036	
32	CH3	3.69	s	52.413	

*Chemical Shift References

The ^{1}H and ^{13}C chemical shifts are referenced to DMSO-d6 at 2.51 and 39.5 ppm, respectively.

NOESY table for 12c in DMSO-d6 at 27 ℃

No.	F2 (ppm)	F1 (ppm)	Abs. Volume	Strength	Assignments
1	2.27	3.12	0.2	medium	3a, 4
2	2.27	3.74	0.1	weak	3a, 2a
3	2.34	2.81	1.1	strong	13b, 13a
4	2.81	2.34	1.0	strong	13a, 13b
5	2.81	3.08	0.1	weak	13a, 4
6	2.81	3.74	0.0	weak	13a, 2a
7	3.08	2.27	0.1	medium	4, 3a
8	3.08	3.74	0.0	weak	4, 2a
9	3.12	2.27	0.2	medium	4, 3a
10	3.12	3.74	0.1	weak	4, 2a
11	3.74	2.27	0.1	weak	2a, 3a
12	3.74	2.81	0.0	weak	2a, 13a
13	3.74	3.08	0.1	weak	2a, 4
14	3.74	3.12	0.1	medium	2a, 4

^{1}H-NMR spectrum of 12d

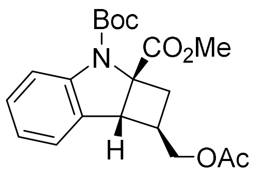

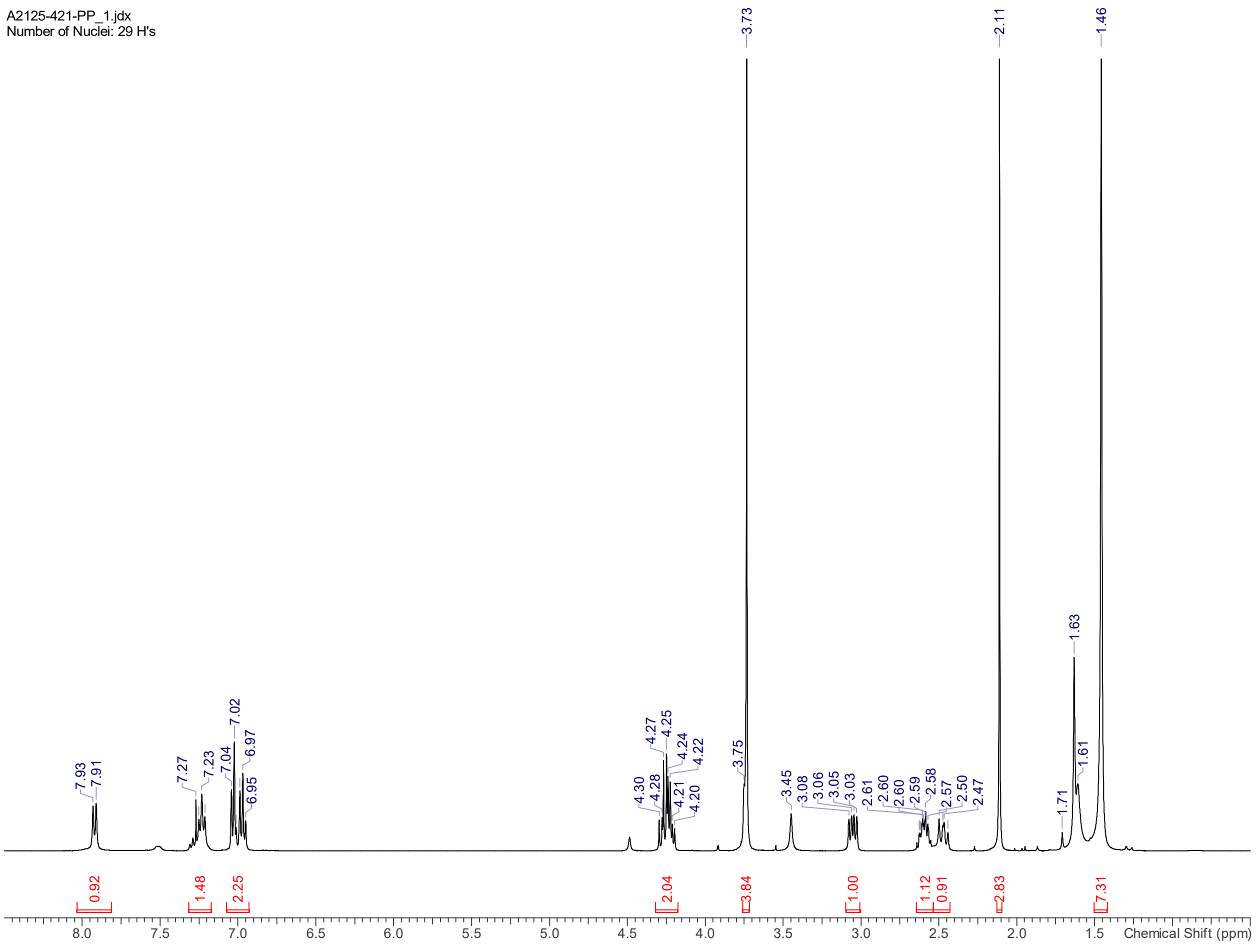

^{13}C-NMR spectrum of 12d

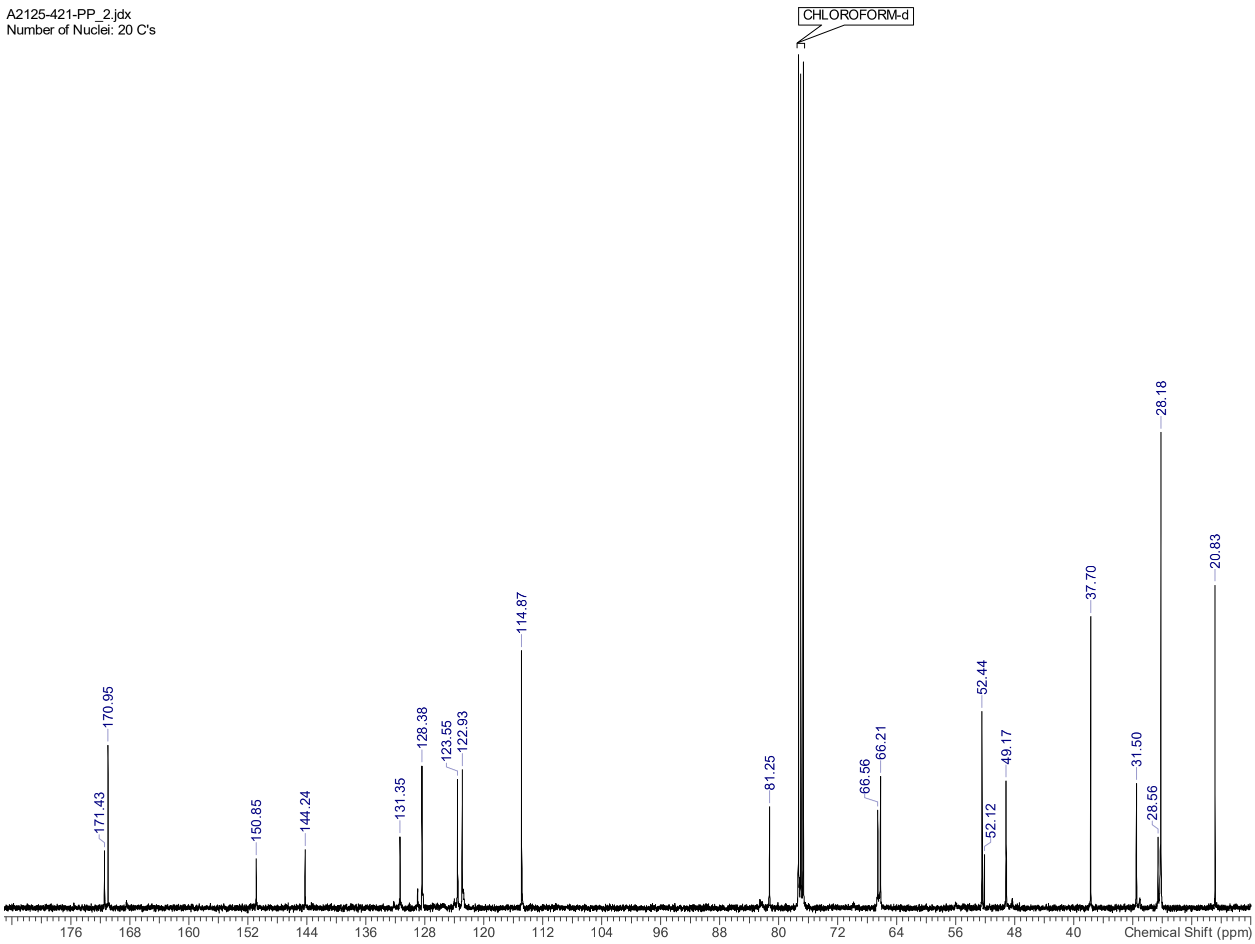

`

^{1}H-NMR spectrum of 12e

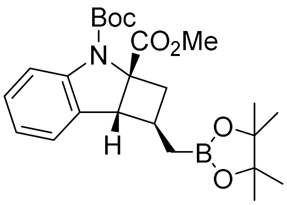

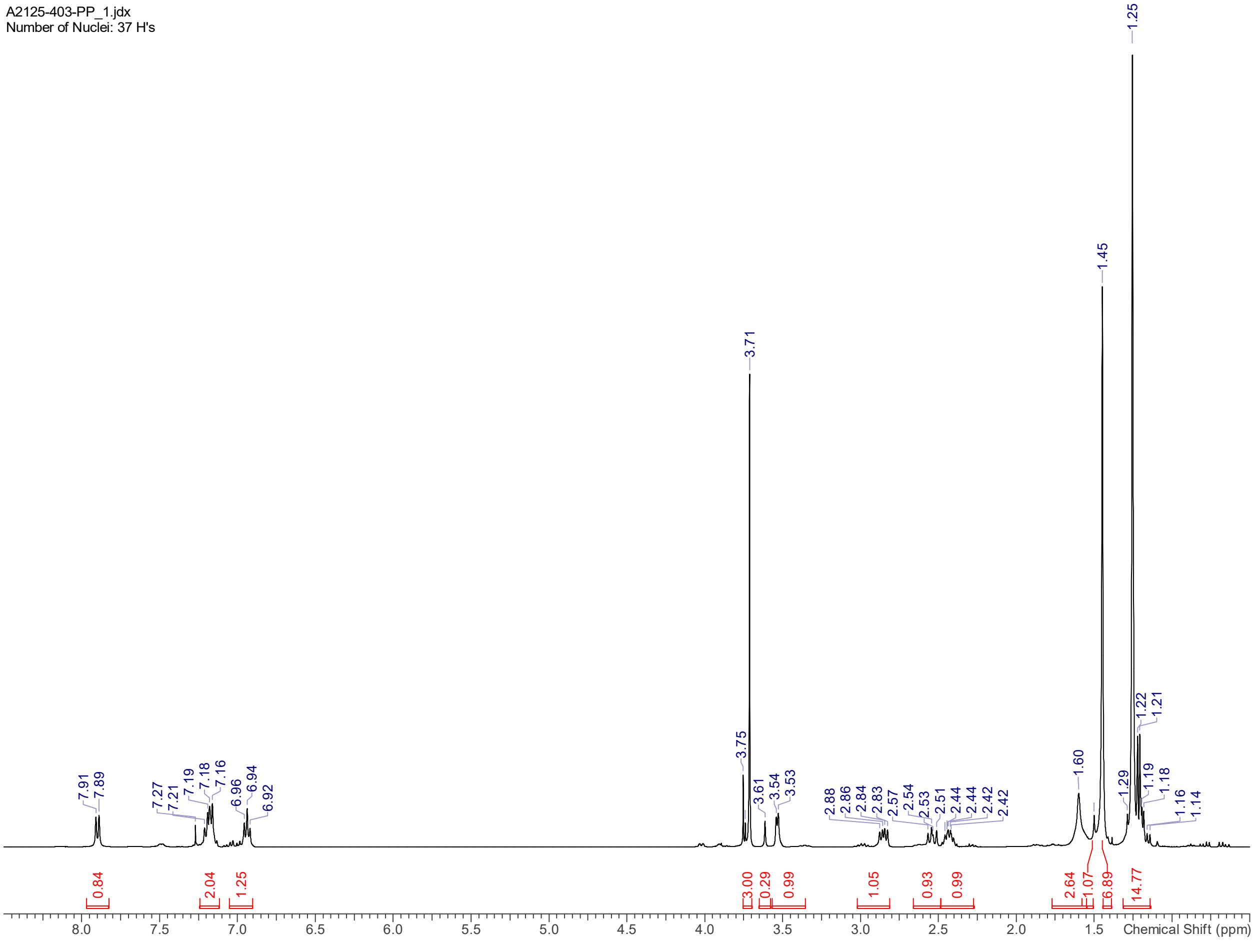

^{13}C-NMR spectrum of 12e

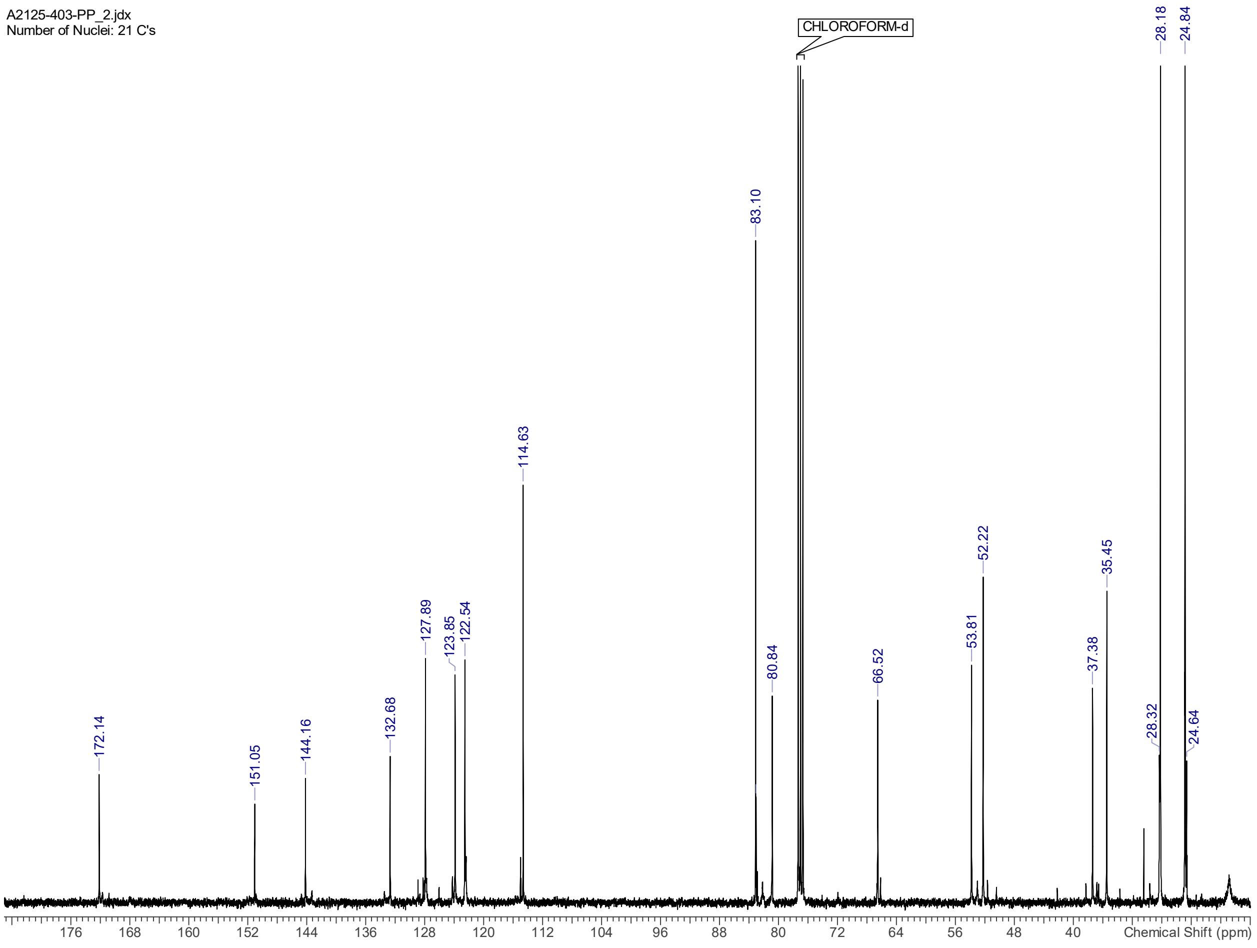

^{1}H-NMR spectrum of 12f

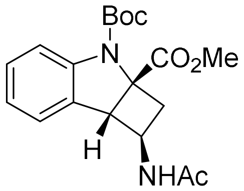

A2AF9-079-02_1.jdx
Number of Nuclei: 28 H's

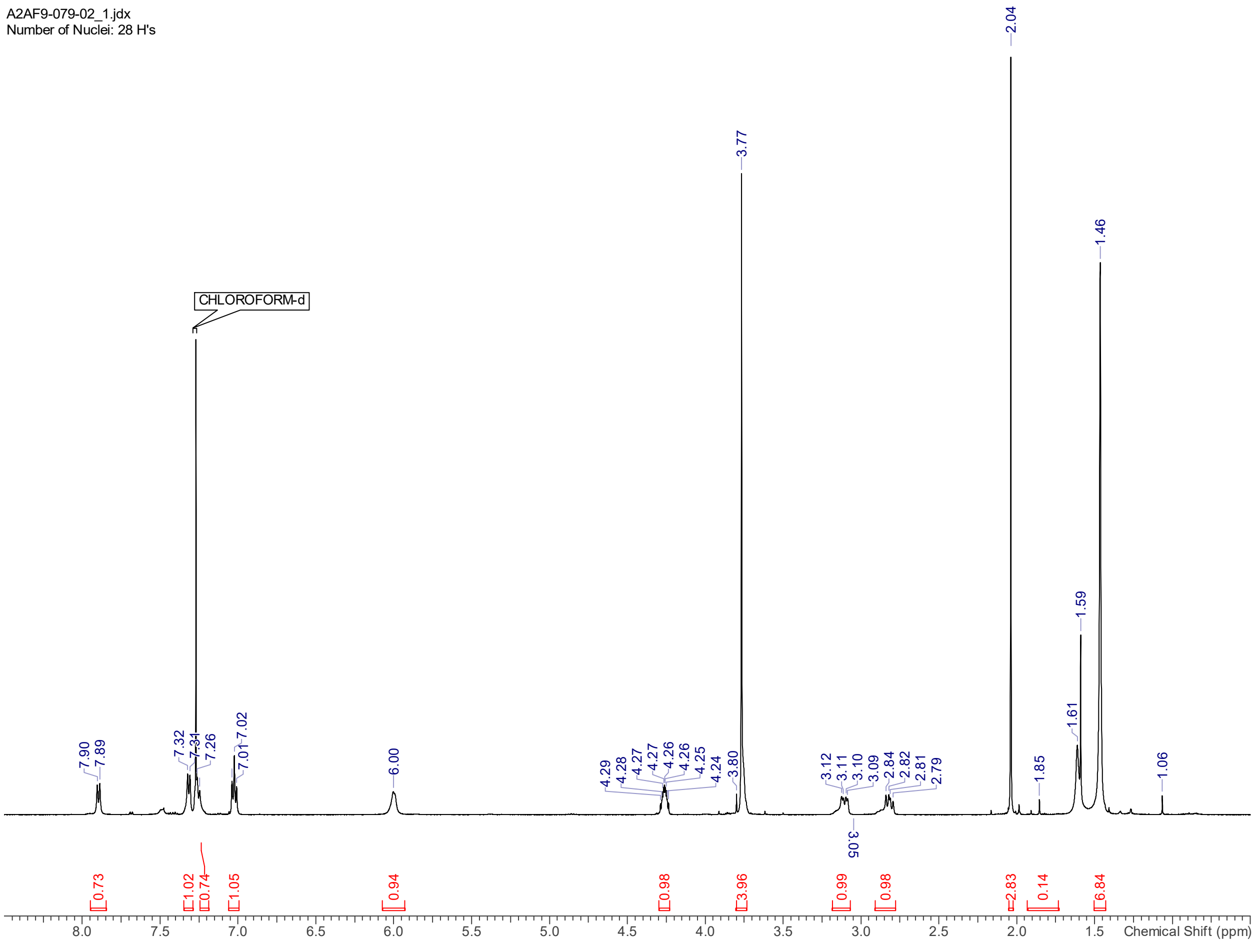

^{13}C-NMR spectrum of 12f

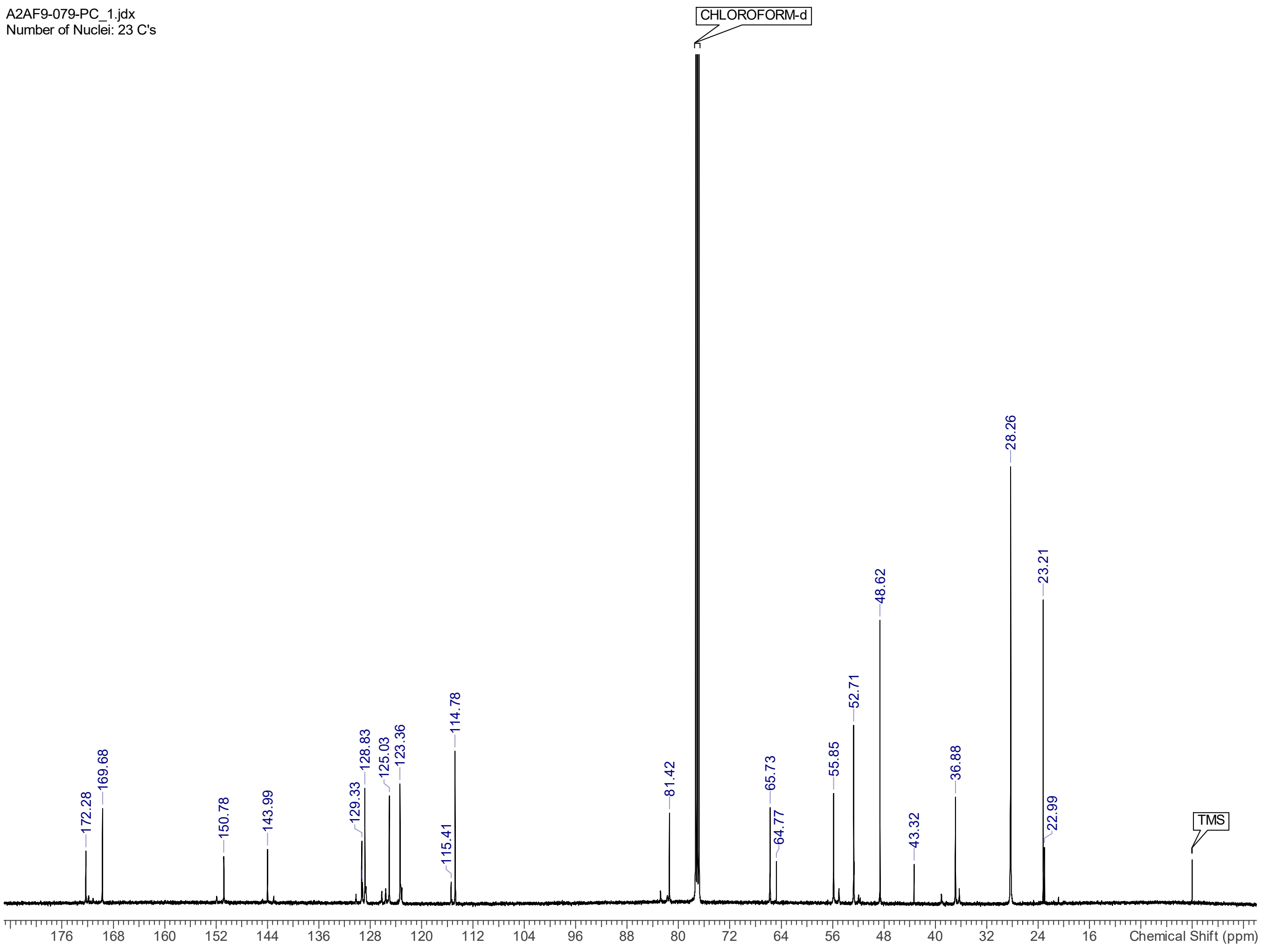

^{1}H-NMR spectrum of 12g

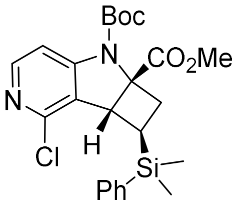

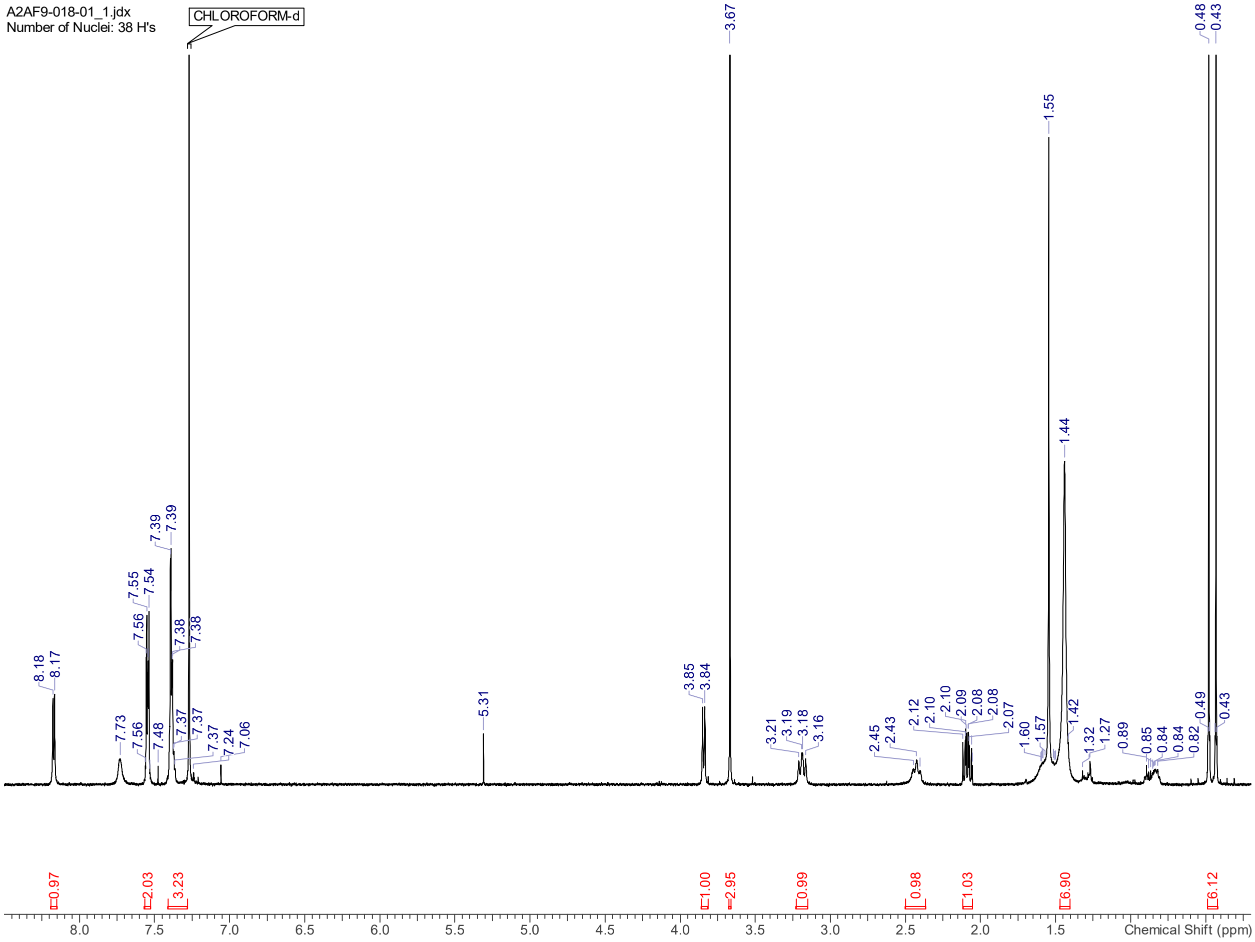

^{13}C-NMR spectrum of 12g

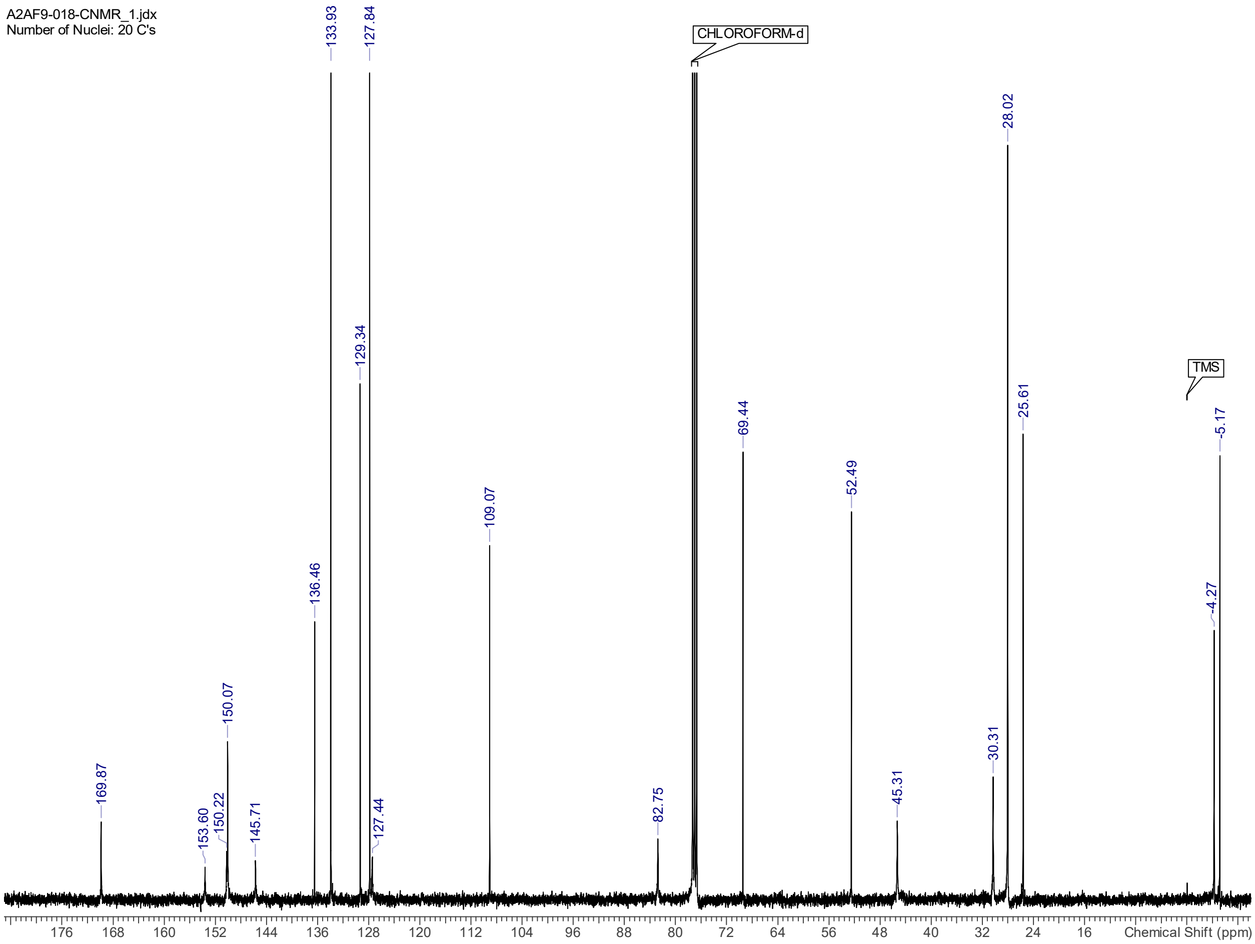

^{1}H-NMR spectrum of 12h

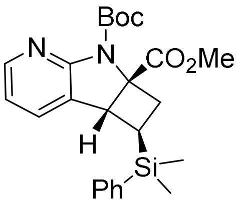

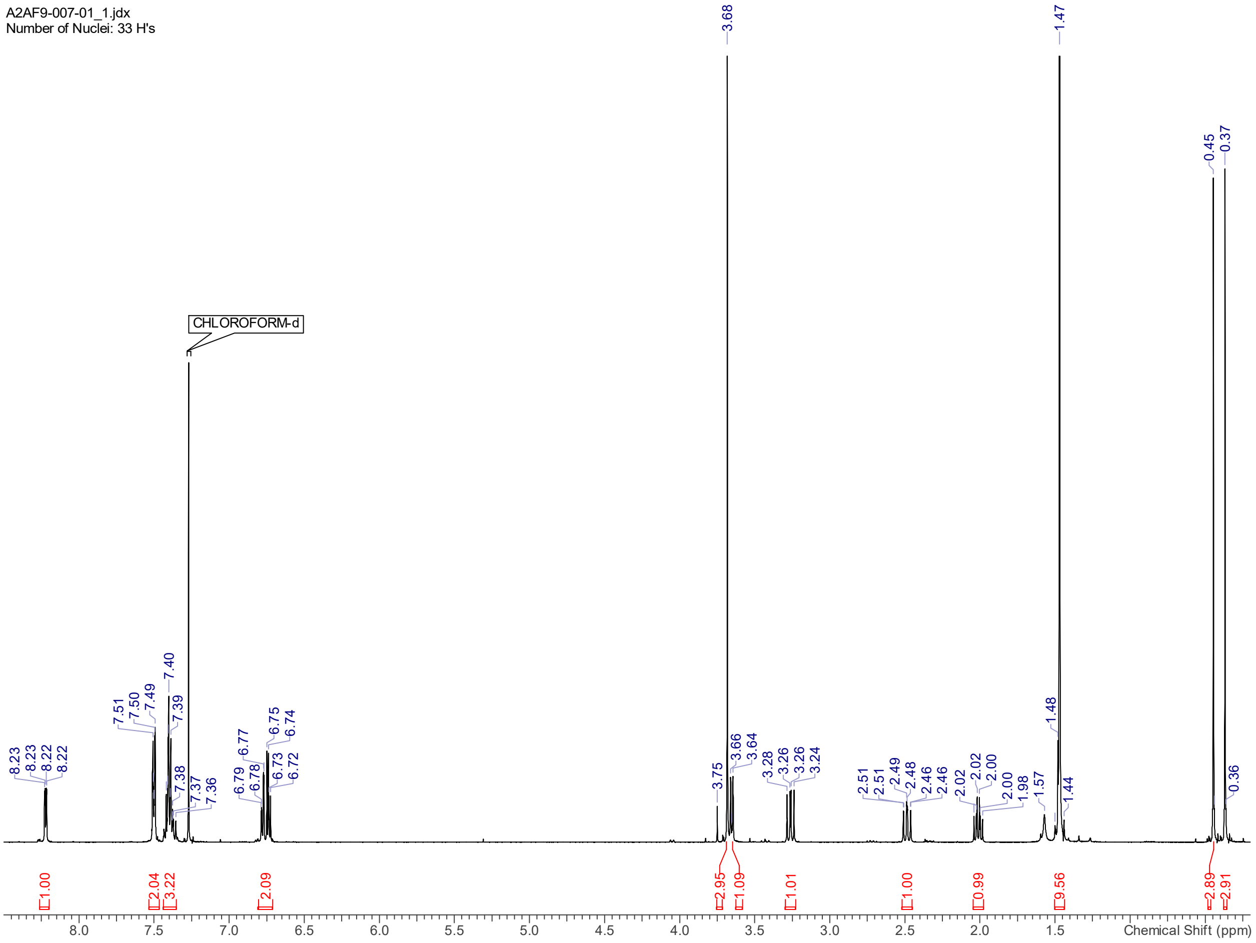

^{13}*C-NMR spectrum of 12h*

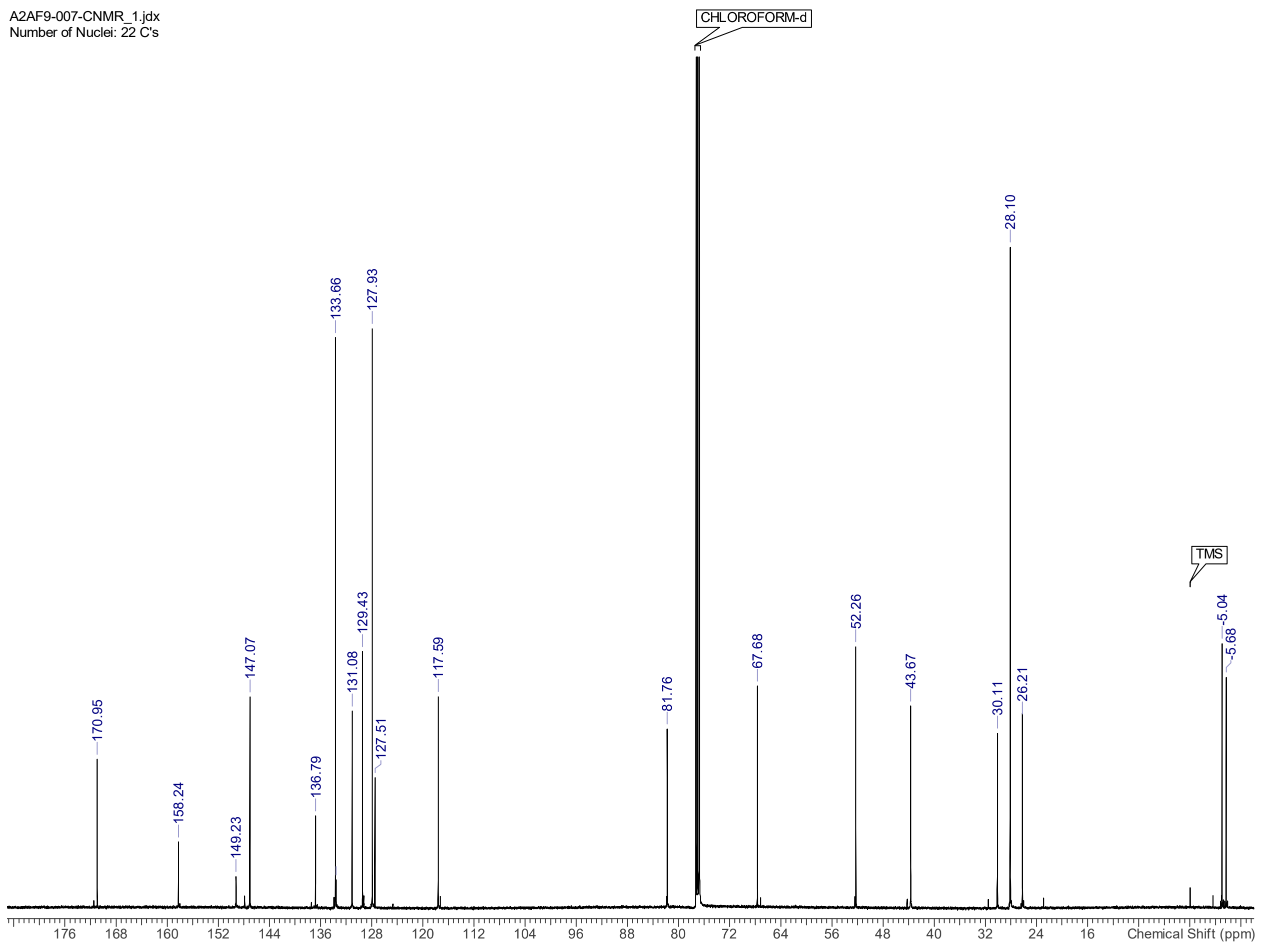

^{1}H-NMR spectrum of 12i

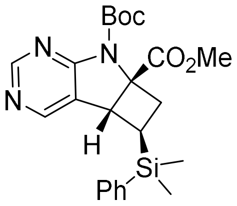

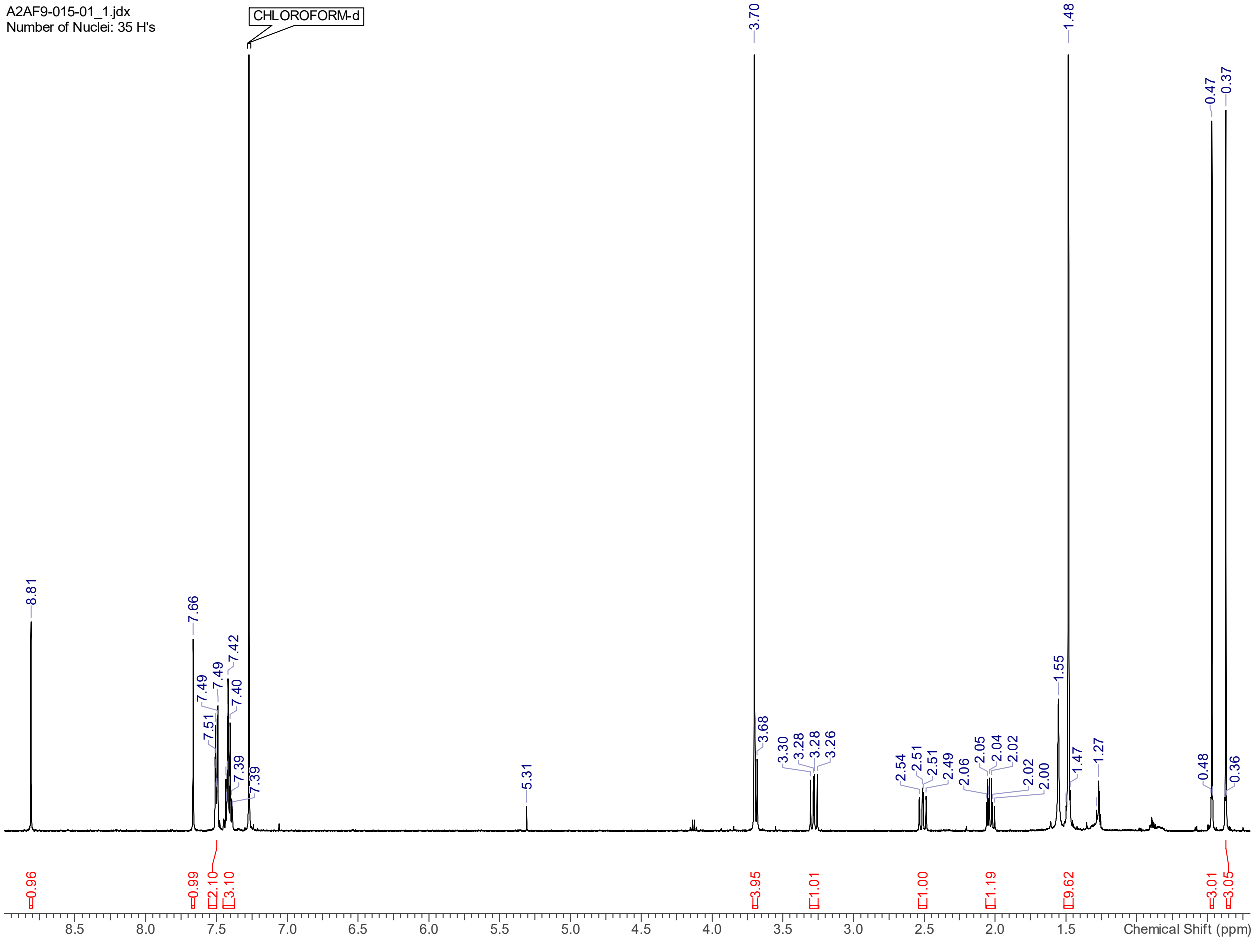

^{13}C-NMR spectrum of 12i

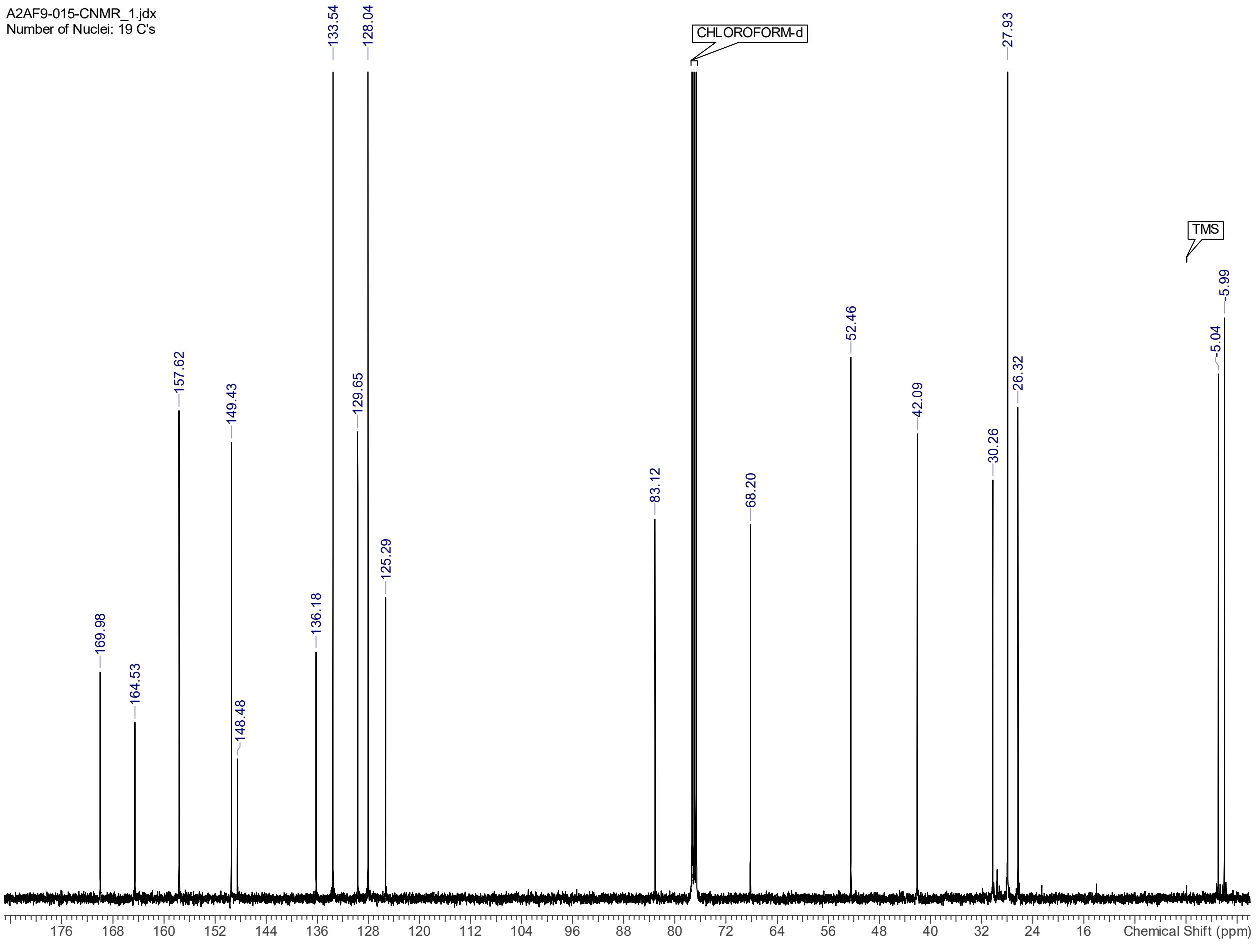

^{1}H-NMR spectrum of 12j

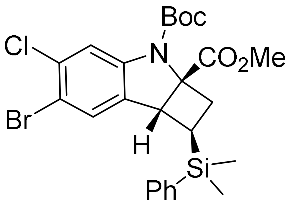

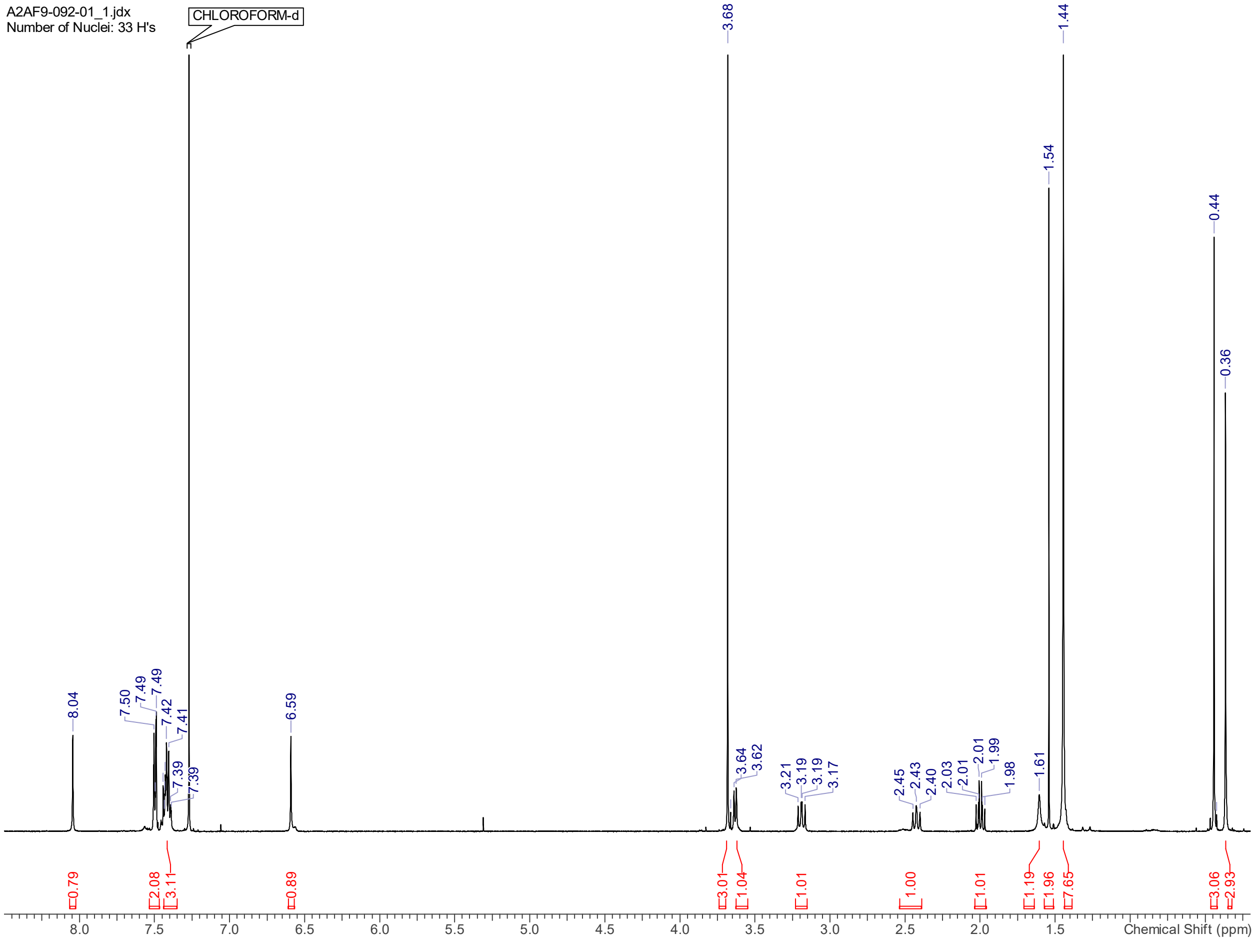

^{13}C-NMR spectrum of 12j

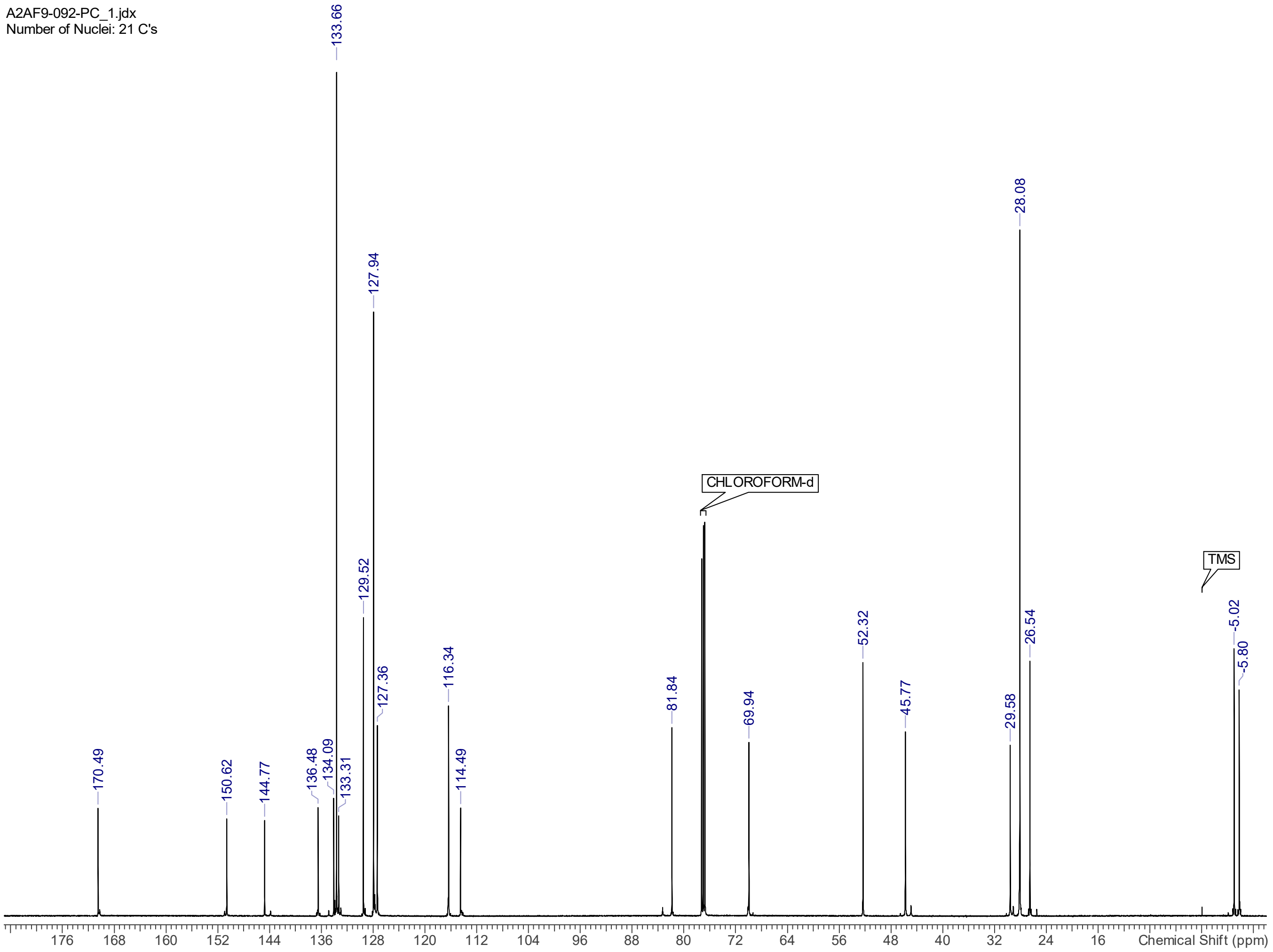

^{1}H-NMR spectrum of 12k

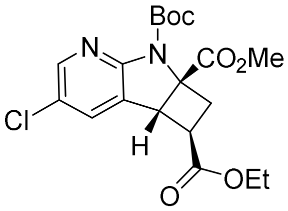

A2AF9-125-PH_1.jdx
Number of Nuclei: 26 H's

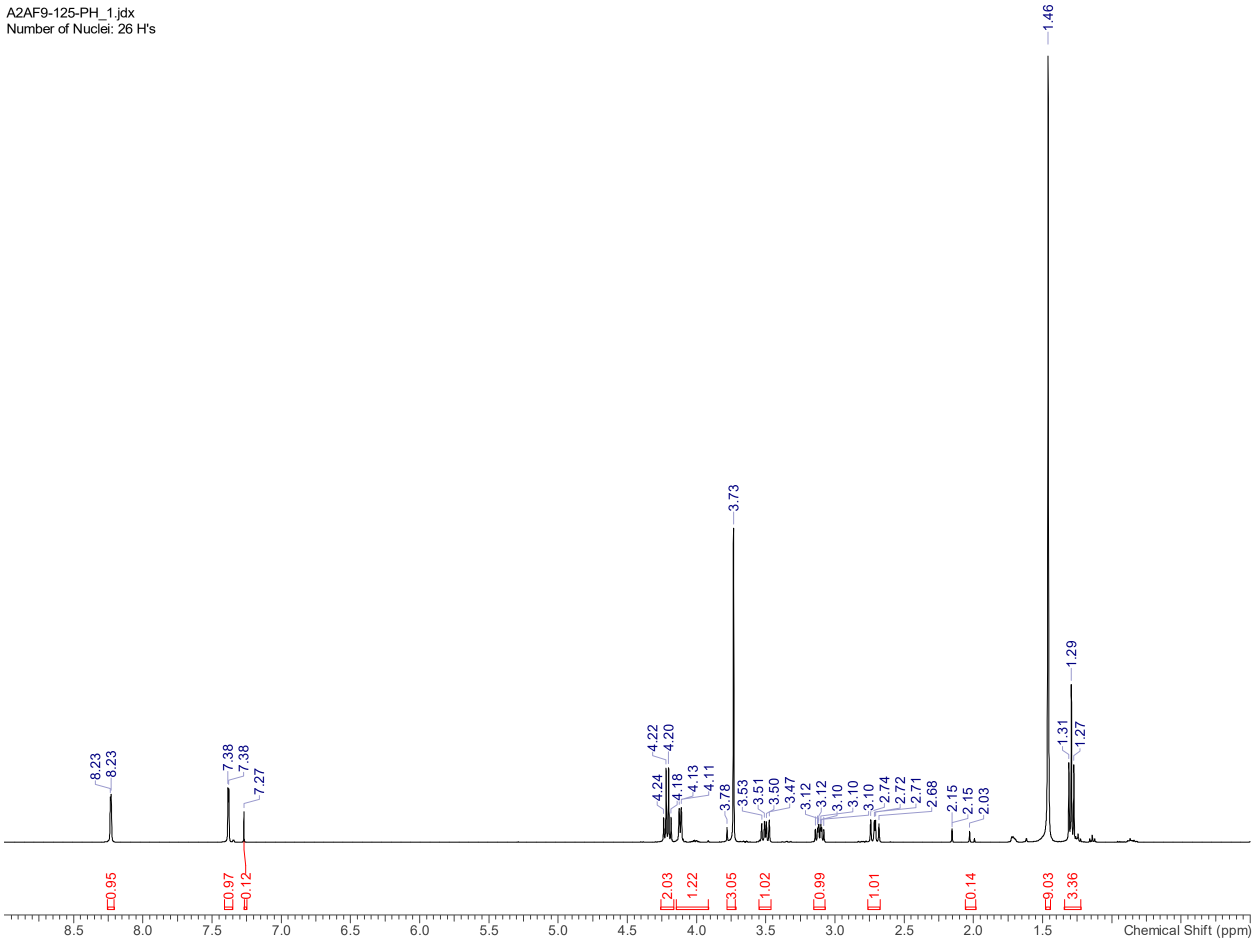

^{13}C-NMR spectrum of 12k

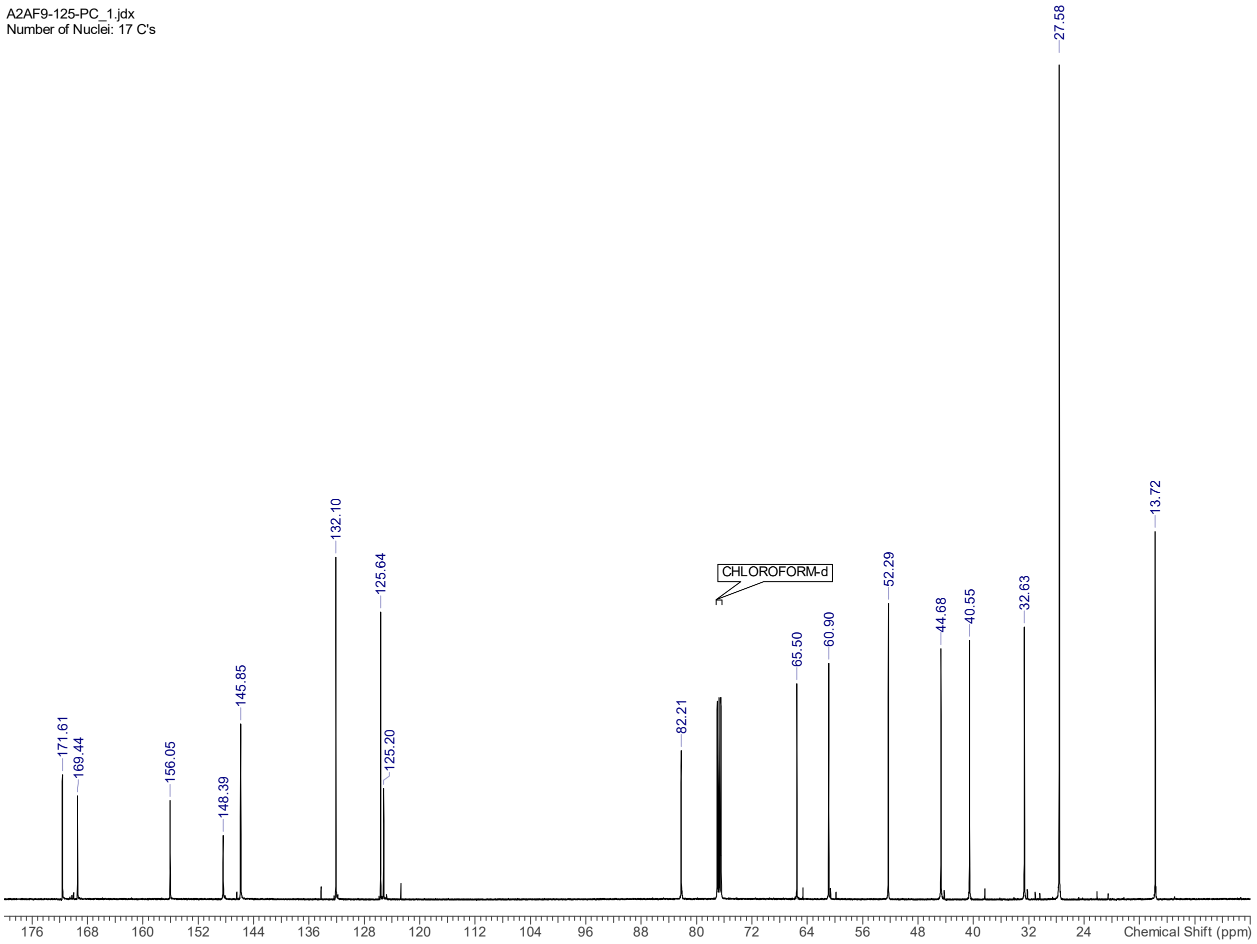

References

1 J. R. Lakowicz, *Principles of Fluorescence Spectroscopy*, Springer US, Boston, MA, 3rd edn., 2006.

2 E. R. Sauvé, J. Paeng, S. Yamaguchi and Z. M. Hudson, *J. Org. Chem.*, 2020, **85**, 108–117.

3 B. Vigante, K. Leitonas, D. Volyniuk, V. Andruleviciene, J. Simokaitiene, A. Ivanova, A. Bucinskas, J. V Grazulevicius and P. Arsenyan, *Chem. – Eur. J.*, 2019, **25**, 3325–3336.
